# Autonomous Activation of a Gated Chemiluminescent Photosensitizer Enables Targeted Photodynamic Therapy in Tumor Cells

**DOI:** 10.1021/jacs.5c06761

**Published:** 2025-07-23

**Authors:** Wenwu Peng, Tianjiao Zhou, Lifan Hu, Vivien Vankann, Toszka Bohn, Tobias Bopp, Seah Ling Kuan, Tanja Weil

**Affiliations:** † 28308 Max Planck Institute for Polymer Research, 55128 Mainz, Germany; ‡ State Key Laboratory of Natural Medicines, Department of Pharmaceutics, China Pharmaceutical University, Nanjing 210009, China; § Institute of Immunology of the Research Center for Immunotherapy (FZI), University Medical Center, 9182 Johannes Gutenberg University Mainz, 55131 Mainz, Germany; ∥ German Cancer Consortium (DKTK), 69120 Heidelberg, Germany; ⊥ University Cancer Center (UCT) Mainz, 39068 University Medical Center Mainz, 55131 Mainz, Germany; # Institute for Quantitative and Computational Biosciences (IQCB), 55128 Mainz, Germany; ∇ Centre for Healthy Ageing, Johannes Gutenberg-University Mainz, 55128 Mainz, Germany

## Abstract

Chemiluminescence-based photodynamic therapy (CLPDT) offers a promising solution to the light penetration limits of traditional PDT. However, it lacks spatiotemporal control. Intracellularly activated, self-luminescent PDT agents *via* a molecular logic gate switch may address this key limitation. We report the synthesis of the self-activating, chemiluminescent photosensitizer (PS) that enables tumor microenvironment-controlled PDT applications. This system integrates a dioxetane-based (Diox) chemiluminescent scaffold with a ruthenium-based (Ru) PS through an oxidation and pH-sensitive linker to enable an AND-gated activation mechanism. The Diox@Ru conjugate is selectively activated by elevated intracellular reactive oxygen species (ROS), characteristic of aggressive cancer phenotypes arising from altered cell metabolism. Upon exposure to ROS (in this case, hydrogen peroxide), the boronic acid ester protecting group of the dioxetane is cleaved, initiating localized chemiluminescence that directly excites the Ru­(II) PS to generate cytotoxic singlet oxygen (^1^O_2_). Importantly, Diox@Ru remains inert under physiological conditions (neutral pH, low ROS) as well as in the acidic, ROS-rich extracellular tumor milieu (slightly acidic, high ROS). Its activation is confined to the intracellular space of glycolytic cancer cells with mildly alkaline, ROS-rich cytoplasm; and proceeds autonomously, without the need for external light irradiation. In both two-dimensional (2D) monolayer cultures and three-dimensional (3D) tumor spheroid models, Diox@Ru exhibits robust luminescence and efficient ^1^O_2_ production, resulting in potent cytotoxic effects. These findings present a versatile platform for autonomous activation of self-luminescent PDT agents and highlight the promise of logic-gated chemiluminescence for spatially controlled therapy in complex biological settings.

## Graphical abstract

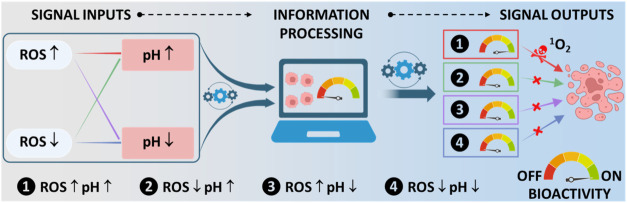

## Introduction

Photodynamic therapy (PDT) has emerged as a clinically validated, minimally invasive cancer treatment that targets tumor tissue with high precision.^1–5^ It induces apoptosis (controlled cell death) in cancer cells using a photosensitizer (PS) by light-controlled generation of singlet oxygen (^1^O_2_).^4–6^ This approach leads to enhanced ^1^O_2_-mediated toxicity with high spatial control through the precise positioning of an external light source, thereby resulting in low systemic adverse effects.^3,7^ However, the low efficiency of many PS as well as the low penetration depth of the external light pose a major challenge that greatly limits the applicability of PDT. Until now, PDT has been a standard of care for the treatment of skin cancer but therapeutic efficacy in deep-seated solid tumors remains challenging.^3,8^

Recent efforts to advance PDT have focused on developing more efficient and chemically robust PSs.^3,4,6,7,9,10^ Polypyridyl ruthenium (Ru­(II)) complexes represent a promising class of inorganic PS capable of generating ^1^O_2_ with high quantum yields and exhibiting superior photostability^2,5,11^ compared to conventional organic PS, which are often limited by rapid photobleaching.^12,13^ Importantly, these complexes offer mechanistic versatility in photodynamic applications,^5,14^ as their activity can be tuned to proceed *via* either a Type II mechanism, relying on molecular oxygen to produce ^1^O_2_, or a Type I pathway, involving electron or hydrogen atom transfer. The latter enables ROS generation under low-oxygen conditions, making Type I PDT particularly advantageous for treating hypoxic tumors or poorly vascularized tissues. However, their activation typically requires excitation in the 400–600 nm range, which does not circumvent the limitation of poor tissue penetration.^2,5,9,15^ Alternative strategies, such as the use of near-infrared (NIR)-responsive osmium-complexes^16^ or upconversion nanoparticles^17^ have extended the optical window of PDT. Yet, these systems still rely on externally applied light and thus face translational challenges.^1,3,7,10^

To overcome the reliance on external light, chemiluminescence-based photodynamic therapy (CLPDT) has emerged as a compelling strategy that exploits endogenous chemical stimuli to generate light *in situ* that activates the PS to produce ^1^O_2_.^18–20^ However, the spatial control inherent to externally applied light in conventional PDT is lost in CLPDT, making selective activation in diseased tissue a central design challenge. Thus, the chemiluminescent system has to be responsive to tumor-cell specific signals, while remaining quiescent in healthy tissue.

The tumor microenvironment (TME) and intracellular environment offers several unique signals that can be harnessed for conditional activation, including elevated levels of ROS and aberrant pH profiles.^18–20^ For instance, fast-growing, glycolytic tumor cells often display an abnormal metabolism and they exhibit high intracellular ROS levels, particularly higher rate of generation of hydrogen peroxide (10–50 μM, up to 0.5 nmol/10^4^ cells/h),^21–24^ coupled with a slightly alkaline cytosolic pH (7.3–7.6), in contrast to the acidic extracellular space (pH ∼6.5, H_2_O_2_, 10–50 μM) and physiological intracellular conditions (pH 7.0–7.2, H_2_O_2_, ∼100 nM).^21,23–29^ These characteristics provide an opportunity to implement molecular logic gates, such as AND gates that require two distinct stimuli for activation, enhancing cancer-cell specificity and minimizing off-target effects.

While luminol^30,31^ - and peroxyoxalate^8,20^ -based systems have been explored for CLPDT, their utility is constrained by requirements for higher alkaline pH (pH > 8), their poor stability in aqueous media and nonselective activation.^18,32–35^ Schaap’s adamantylidene-1,2-dioxetanes offer a promising alternative due to their exceptional stability and controlled activation by deprotection of phenolic groups *via* enzyme or ROS-mediated triggers.^36–38^ In particular, phenylboronic acid esters are cleaved selectively by H_2_O_2_, and their oxidation kinetics can be modulated by pH, providing an opportunity for dual-stimulus control in biologically relevant environments.^39^ Although Schaap’s dioxetanes have been extensively studied in chemical sensing, their application in CLPDT remains underexplored, with only one prior example using an organic PS for dark dynamic therapy with a single stimulus nitroreductase-mediated release kinetics over 72 h.^40^

Here, we report the rational design of the self-activating CLPDT agent, Diox@Ru, that integrates a Schaap’s dioxetane chemiluminescent donor with a Ru­(II)-based PS *via* a boronic ester-based AND gate linker, which is responsive to the intracellular microenvironment of cancer cells (Figure 1). The Diox@Ru conjugate remains inert under physiological conditions (physiological pH, low ROS) and within the acidic extracellular tumor milieu (low pH, high ROS),^41^ but becomes selectively activated in cancer cell cytoplasm, where elevated ROS levels and mildly basic pH coexist. This unique activation profile enables precise, autonomous generation of ^1^O_2_ *in situ*, without the need for external light. We demonstrate the efficacy of Diox@Ru in both two-dimensional (2D) and three-dimensional (3D) models of lung carcinoma cells, as well as triple-negative breast cancer (TNBC), which is a highly aggressive and therapeutically challenging cancer type characterized by enhanced glycolytic metabolism and ROS-mediated stress.^42,43^ This work highlights a new paradigm for spatially controlled PDT through molecularly gated chemiluminescence, and sets the stage for developing next-generation, self-luminescent and autonomous therapeutic systems that function selectively in the complex biochemical landscapes of solid tumors.

**Figure 1:**
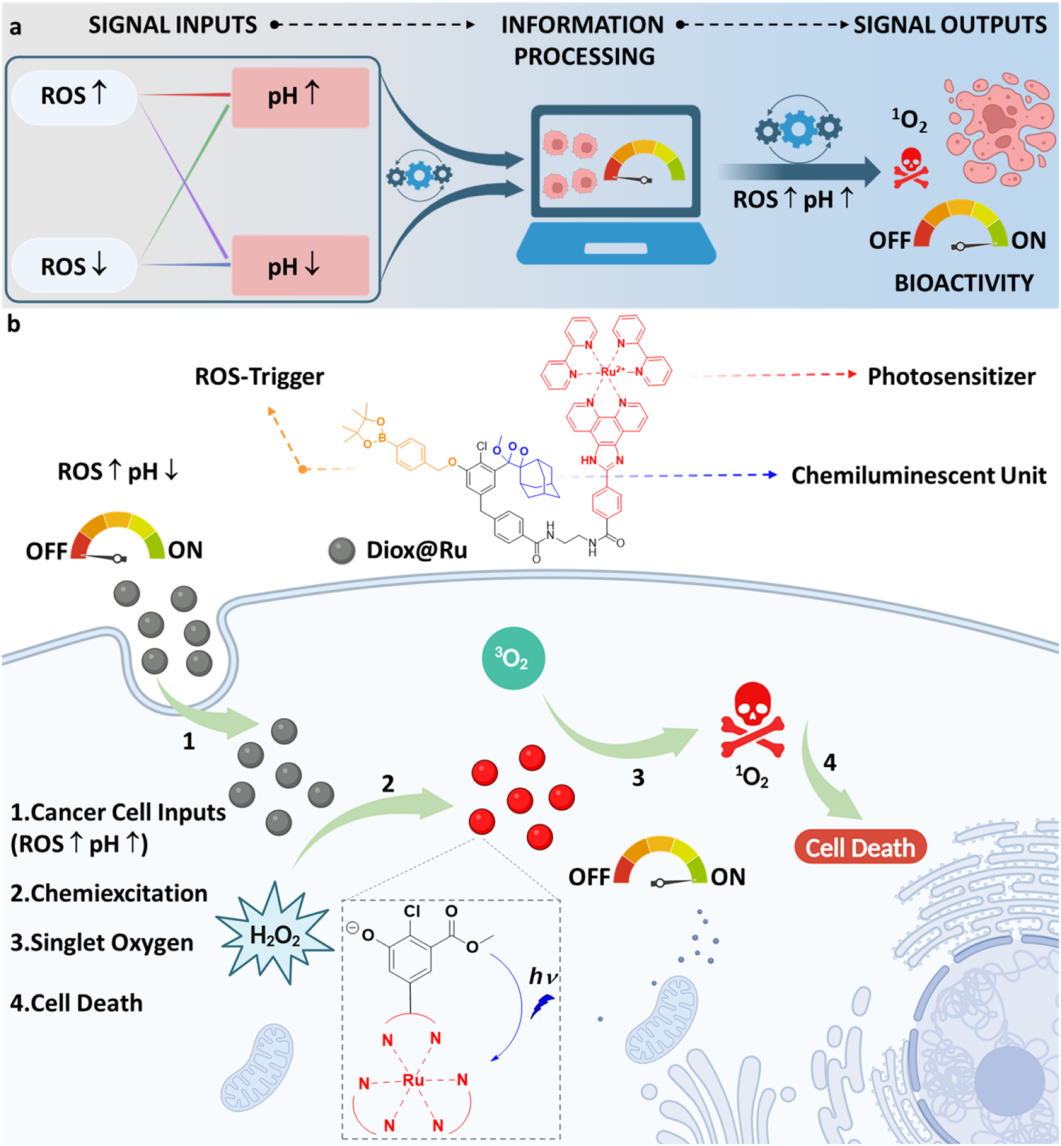
Design of the self-activating “AND” gated chemiluminescent photosensitizer for controlled chemiluminescent photodynamic applications in glycolytic tumor cells. Figure is created with Biorender.com.

## Results and Discussion

### Design, Synthesis and Characterization of Diox@Ru

The design concept of Diox@Ru is depicted in Figure 1. Diox@Ru consists of three distinct molecular components: (1) the boronic acid ester activator group (ROS and pH *input signals*), (2) the chemiluminescent unit (the light-emitting group that *transforms the signal*) and (3) the photosensitizer (^1^O_2_-generation as *output signal*). Diox@Ru preserves the dormant (“***OFF***”) state until it is activated by high H_2_O_2_ concentrations at cytosolic pH inside cancer cells (pH 7.3–7.6), which initiate cleavage of the phenylboronic acid ester that protects the phenol group (“***ON***”).

The synthesis scheme of Diox@Ru (**12**) is shown in Figure 2a, and the preparation of all compounds as well as their characterizations are given in the Supporting Information. First, the caged chemiluminescent molecule (**10**) bearing the stimulus-responsive boronic acid ester group and the Ru-based photosensitizer (**11**) were synthesized, respectively. Compound **10** was prepared by Williamson ether synthesis, featuring an *N*-hydroxysuccinimide-activated carboxyl group. Purification was achieved using flash silicon column chromatography, which preserves structure integrity, yielding **10** as a white solid in 85% yield (Supporting Information). Concurrently, the Ru-based PS (**11**) was prepared by coordinating commercial Ru­(bpy)_2_Cl_2_ with a functional ligand (**11–3**, Figure 2b) that provided a free amine group for subsequent amidation. **11** was purified by preparative high-performance liquid chromatography (HPLC) and isolated as red solid in 60% yield (Supporting Information). Prior to molecular conjugation, preoxidation was implemented by forming the peroxide bond in compound **10**, a critical modification to prevent PS-accelerated decomposition of the final Diox@Ru complex under irradiation.^37^ Finally, the target compound Diox@Ru (**12**) was successfully synthesized through an amidation reaction between the chemiluminescent group and the functionalized photosensitizer. However, the inherent acid lability of the boronic acid ester group, which readily converts to boronic acid under acidic conditions,^44,45^ combined with the high polarity imparted by the polypyridyl Ru­(II) complex in Dixo@Ru, renders conventional purification methods such as column chromatography or HPLC impractical. To maximize reaction efficiency, a stoichiometric excess of the chemiluminescent moiety was used during coupling with the Ru-based photosensitizer. Unreacted chemiluminescent educts were subsequently removed by selective diethyl ether washing, allowing the final Diox@Ru (**12**) conjugate to be isolated as a red solid *via* ether precipitation in 88% yield. As controls to compare the physicochemical properties and the biological effects of compound **12**, the chemiluminescent unit (Diox, compound **16**) was prepared (Figure 2c). All compounds have been characterized by nuclear magnetic resonance spectroscopy (NMR) and mass spectrometry (MS) in the Supporting Information.

**Figure 2:**
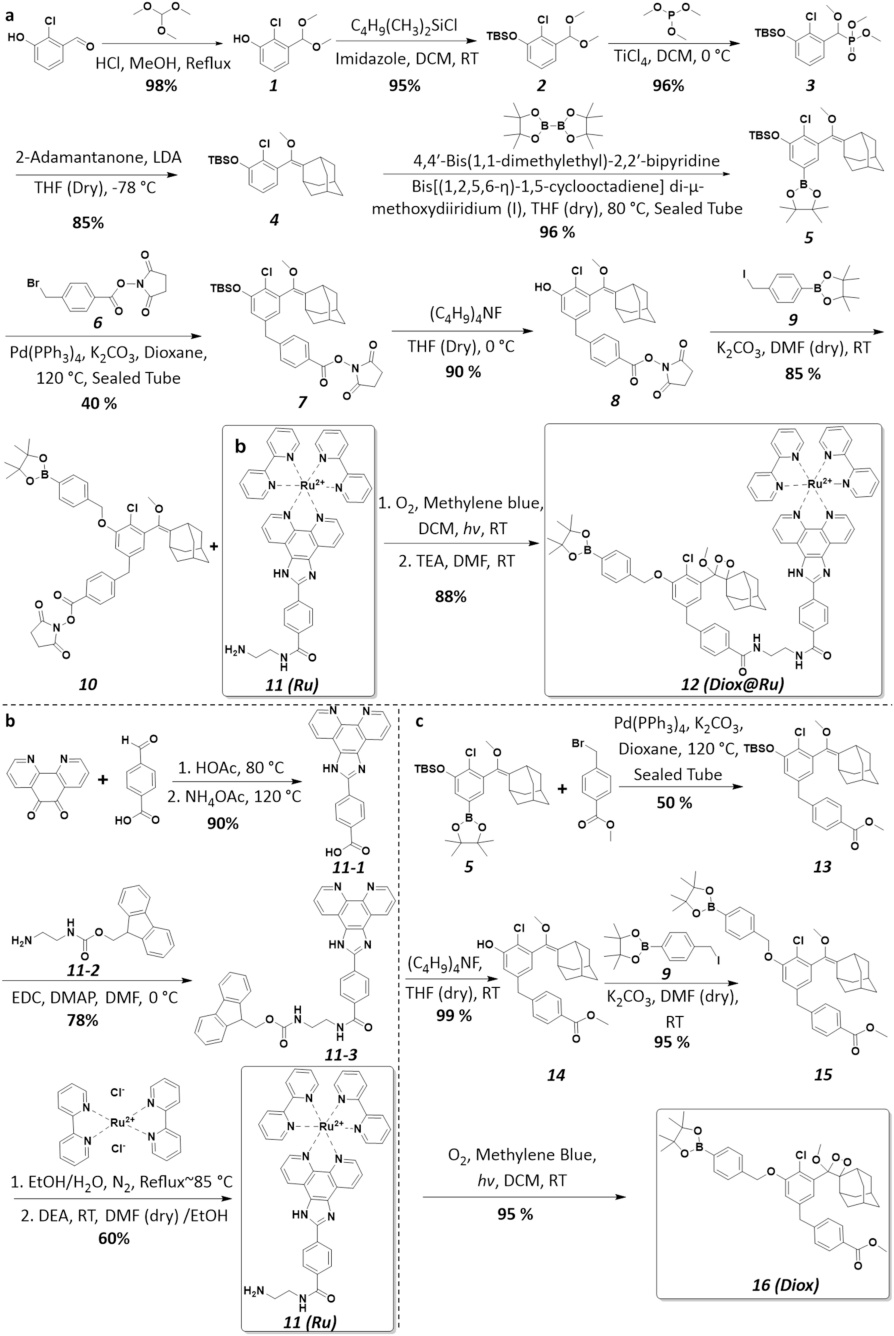
Synthesis scheme of (a) Diox@Ru, (b) Ru and (c) Diox.

Diox@Ru (**12)** was characterized by high-performance liquid chromatography–mass spectrometry (HPLC-MS, Figure S1b), demonstrating a primary chromatographic peak at 7.5 min with corresponding mass signals at *m*/*z* 1463.7 ([M–H^+^–2Cl^–^]^+^, calc. 1463.1) and 732.3 ([M–2H^+^–2Cl^–^]^2+^, calc. 731.5), confirming its molecular identity. A second peak observed at 6.5 min (*m*/*z* 1381.6 and 690.8) was attributed to hydrolysis of the phenylboronic ester moiety to phenylboronic acid, as boronic acid ester is known to transform to boronic acid under acidic conditions, which is used in HPLC chromatography.^44,45^ These results underscore the importance of using ether precipitation for purification, as conventional chromatographic techniques were found to accelerate decomposition. The molecular structure of Diox@Ru (**12**) was further corroborated by ^1^H NMR analysis (Figure S1c), which revealed characteristic signals for both molecular components: The singlet at δ 14.66 ppm (1H) corresponds to the ruthenium polypyridyl complex, while the multiplet spanning δ 2.23–1.41 ppm (14H) and the singlet at δ 1.29 ppm (12H) are assigned to the adamantane moiety and boronic acid ester functionality, respectively. This chemical shift differentiation provides direct evidence of successful conjugation between the stimulus-responsive chemiluminescent group (bearing the boronic acid ester) and the Ru-based photosensitizer and no unreacted educts were detected in the spectrum.

High-resolution ESI (HR-ESI) (Figure S2) reveals a distinct signal at *m*/*z* 731.5164 ([M–2H^+^–2Cl^–^]^2+^, calc. 731.5172), further confirming the successful synthesis and molecular identity of Diox@Ru (**12**). The ultraviolet–visible (UV–vis) spectral analysis shown in Figure S3a,b displays the characteristic absorption (λ_max_ = 459 nm) and emission (λ_em_ = 616 nm) profiles of the ruthenium-based photosensitizer. Diox@Ru exhibits identical photophysical features, as illustrated in Figure S3c,d. This spectral overlap provides strong evidence for the successful coordination of the ruthenium center to the dioxetane scaffold, confirming the formation of the ruthenium-functionalized chemiluminescent conjugate (Diox@Ru).

To assess the stability of Diox@Ru in solution, LCMS analysis was performed under physiological conditions (DPBS buffer at pH 7.4) in both dark and ambient light environments. Identical reaction solutions (400 μM) were prepared for both Diox@Ru and its nongated control compound Me-Diox@Ru (bearing a methyl-substituted trigger group; see **1.3.20 Compound 17 in** Supporting Information), and monitored over time based on the relative integration of LC peaks at 254 nm, normalized to their initial intensity (*t* = 0). As shown in Figure 3a,b, Diox@Ru remained stable in the dark for at least 48 h, with >99% of the compound intact. The only observed change was hydrolysis of the boronic acid ester to the boronic acid, evidenced by a shift in the LC retention time from 7.5 to 6.5 min and confirmed by MS analysis. Under ambient light, however, a new LC peak emerged after 2h (retention time = 5.8 min), corresponding to decomposition of the dioxetane moiety, as verified by mass spectrometry. After 48 h, substantial degradation of the dioxetane core, essential for chemiluminescence, was observed, whereas the molecular structure of Diox@Ru remained largely intact. In comparison, Me-Diox@Ru, revealed excellent dark stability (<1% degradation) and only slowed photodecomposition under ambient light (Figure 3c,d), consistent with the absence of a reactive benzoboronate trigger. Semiquantitative analysis (Figure 3e) revealed that both compounds retain >90% integrity after 2 h of light exposure and >99% in the dark over 48 h. Furthermore, Diox@Ru stored as a solid in the dark remained chemically stable for at least one year. These results demonstrate that Diox@Ru exhibits sufficient stability for long-term solid-state storage and can be reliably used in solution for at least 48 h under dark conditions, supporting its suitability for practical applications.

**Figure 3:**
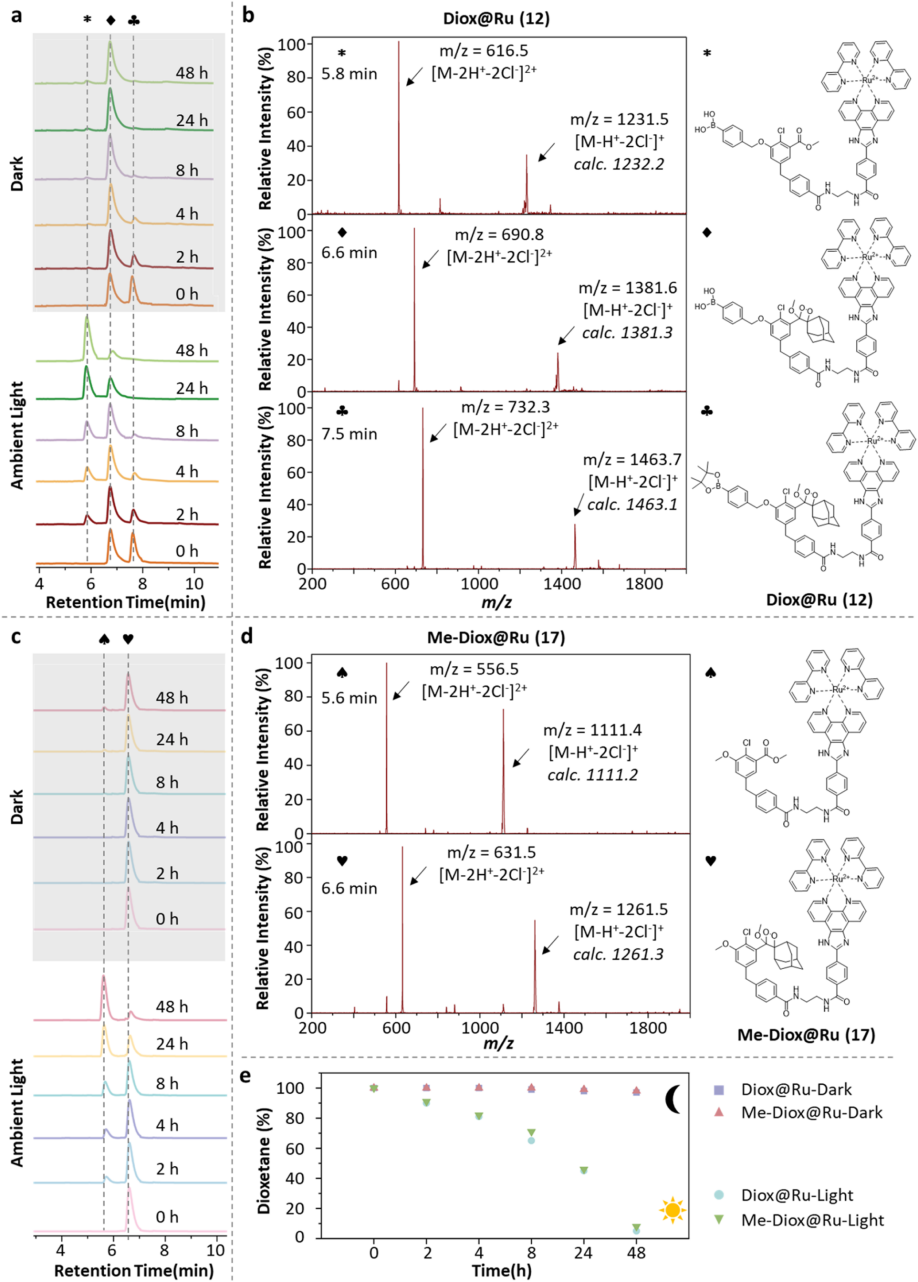
Light-induced decomposition studies of Diox@Ru (**12**, 400 μM in DPBS, pH = 7.4) and Me-Diox@Ru (**17**, 400 μM in DPBS, pH = 7.4) at ambient temperature in darkness and ambient light. Time profile of liquid chromatogram and MS spectra from the LC-MS analysis for (a, b) Diox@Ru and (c, d) Me-Diox@Ru. (e) Decomposition of the dioxetane unit in Diox@Ru and Me-Diox@Ru. Full LC spectra of Figure (a, c) could be found in Figure S4.

### Diox@Ru Generates Chemiluminescence and Singlet Oxygen

Next, the photophysical properties of Diox@Ru (**12**) are compared to the individual functional entities Ru-PS (**11**) and Diox (**16**). The chemiluminescence of Diox (250 μM) was evaluated, revealing that it is selectively activated by H_2_O_2_ (250 μM), producing sustained luminescence (>12 h), whereas negligible emission is observed in the absence of H_2_O_2_ (Figure S5a). Upon activation, the emission of Diox at 480 nm facilitates energy transfer to the Ru complex, resulting in a secondary emission at 608 nm. Notably, Diox@Ru (250 μM), featuring a covalent linkage between Diox and Ru, exhibits markedly enhanced energy transfer efficiency under H_2_O_2_ (250 μM) stimulation (Figures S6a,b and S7), attributed to an intramolecular mechanism, as compared to the weaker intermolecular energy transfer observed in the mixture of individual Diox and Ru (250 μM, Figure S5b–d). As shown in the chemiluminescence kinetics and emission spectra (Figures S6a,b and S7), H_2_O_2_ serves as a critical trigger for luminescence generation. Importantly, the dominant emission peak of Diox@Ru aligns with the spectral characteristics of Ru, confirming that the chemiexcitation of H_2_O_2_-activated Diox effectively excites the Ru photosensitizer (Figure 4a). In addition, the significantly higher chemiluminescence intensity of Diox@Ru compared to Diox in aqueous solutions indicates that Diox@Ru confers resistance to aqueous quenching, unlike pure Diox, whose emission is subject to rapid quenching in this medium, precluding reliable detection.^36,46^ Further analysis of varying Diox@Ru: H_2_O_2_ ratios (1:10, 1:5, 1:1, Figure S6c,d) demonstrates that both chemiluminescent intensity and emission kinetics are strongly influenced by the H_2_O_2_ concentration. Consistently, Figures 4b and S8a,c show a positive correlation between increasing ROS levels and chemiluminescence enhancement. Our experimental results show that Diox@Ru (50 μM) undergoes chemiluminescent activation in the presence of H_2_O_2_ concentrations as low as 25 μM. This threshold is consistent with previous studies reporting activation at ∼12.5 μM for structurally related dioxetane-based compounds,^47^ and aligns with endogenous H_2_O_2_ levels typically found in tumor cells (10–50 μM, up to 0.5 nmol/10^4^ cells/h).^21–24^ While H_2_O_2_ production rates in tumors are often elevated, local accumulation can vary significantly across tumor types, and in some cases, may fall below this threshold.^23,24^ Nonetheless, our findings demonstrate that Diox@Ru functions as a H_2_O_2_-responsive chemiluminescent photosensitizer, with its photodynamic activation tightly regulated by the local oxidative environment.

**Figure 4:**
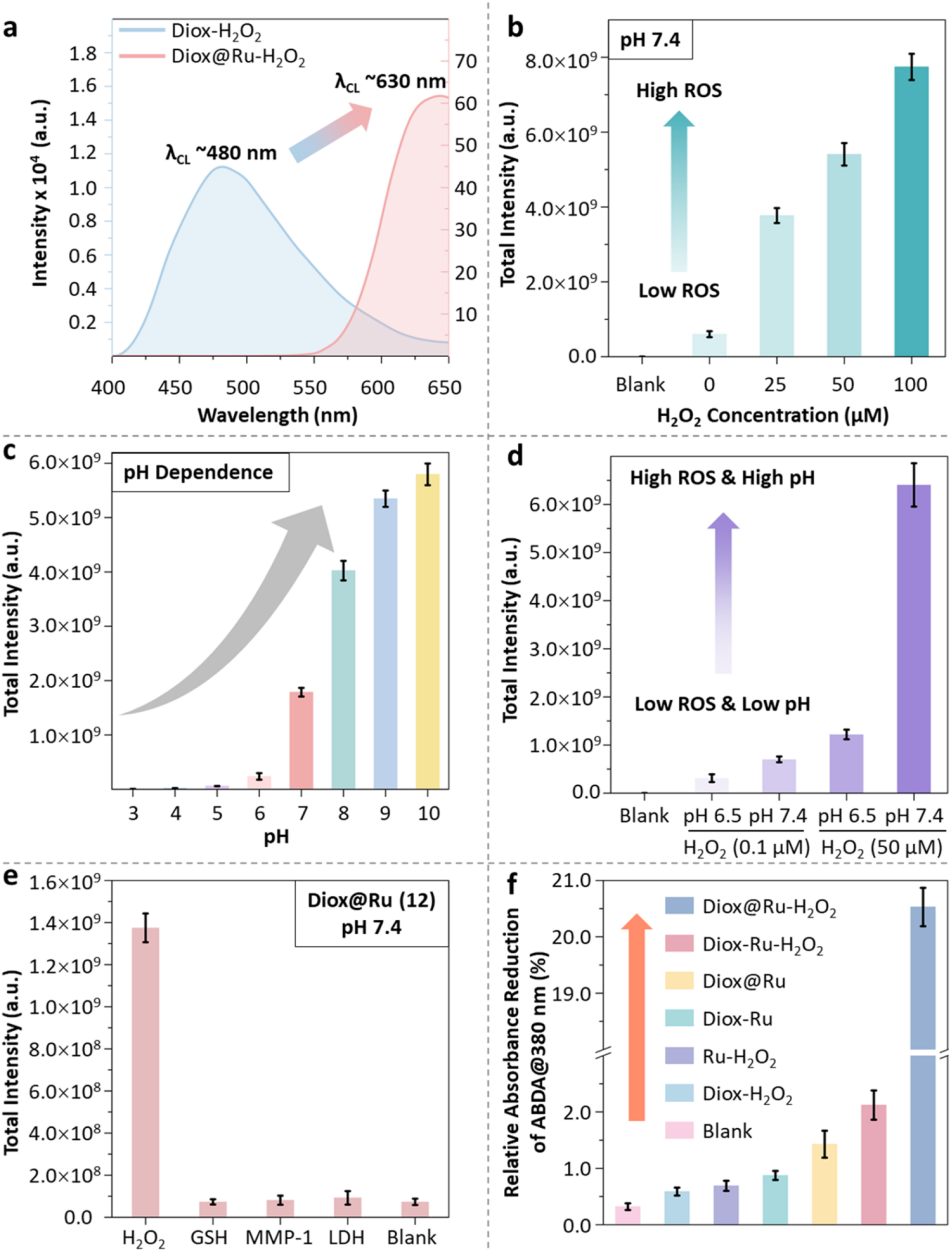
(a) Chemiluminescence (CL) emission spectra of Diox and Diox@Ru (250 μM Diox or Diox@Ru with 250 μM H_2_O_2_, in DPBS, pH = 7.4, after 2h incubation) (b) Total chemiluminescence of Diox@Ru (**12**, 50 μM, DPBS, pH = 7.4) for varying H_2_O_2_ concentrations (0, 25, 50, 100 μM, after 12 h incubation). Data presented as S.E.M, *n* = 3. (c) pH stability of Diox@Ru (250 μM) in Tris–HCl solution (50 mM, after 2h incubation)). (d) Comparison of total chemiluminescence of Diox@Ru (50 μM, DPBS, with 0.1 or 50 μM ROS at pH 6.5 and pH 7.4, after 12h incubation). (e) Chemiluminescence response of Diox@Ru (50 μM, DPBS, pH = 7.4, after 2h incubation) to various biomarkers in cancer cells. Hydrogen peroxide (H_2_O_2_, 50 μM), l-glutathione (GSH, 10 mM), lactate dehydrogenase from porcine heart (LDH, 0.5 units/mL) and matrix metalloproteinase-1 (MMP-1, 500 ng/mL). The blank group was evaluated under identical experimental conditions without the addition of biomarkers. (f) Comparison of ^1^O_2_ generation of Diox@Ru (**12**, 50 μM) with coincubated Diox (**16**) and Ru (**11**) (Diox-Ru, 50 μM) (9,10-anthracenediyl-bis­(methylene)­dimalonic acid (ABDA, 50 μM) in the presence of 250 μM H_2_O_2_ in DPBS (pH = 7.4), after 2 h incubation). Data presented as S.E.M, *n* = 3.

Next, we evaluated the chemiluminescence of Diox@Ru at different pH (pH 3–10). As demonstrated in Figure 4c, the pH-dependent stability profile of Diox@Ru was quantified by measuring the chemiluminescence intensity in tris–HCl solution. Notably, an inverse correlation was observed between the total luminescent output and Diox@Ru stability, wherein elevated emission intensities correspond to progressive destabilization of the Diox@Ru complex. Diox@Ru exhibits markedly enhanced stability under acidic conditions compared to alkaline environments, a characteristic that facilitates the maintenance of its dormant state within the acidic extracellular microenvironment of glycolytic tumors.^42,43^ The observed pH-dependent luminescence could be attributed to a base-catalyzed hydrolysis mechanisms, where hydroxide ions directly cleave the peroxide bonds in Diox@Ru through a nucleophilic attack,^48,49^ representing a fundamentally distinct activation pathway from the H_2_O_2_-mediated chemiluminescent mechanism illustrated in Figure 1.

Having evaluated the chemiluminescence properties in the presence of varying ROS at different pH, we proceeded to investigate the activation of Diox@Ru in various combinations of pH and ROS that are relevant to the extracellular and intracellular environment of glycolytic cancer cells such as TNBC. As these cancer cells have a lower extracellular pH of ∼6.5 ^28^ and a higher intracellular pH of 7.3–7.6,^25,26^ we determined the chemiluminescent properties of Diox@Ru with low *versus* high ROS and pH in tris-HCl solution. The experimental data presented in Figures S8b–d and 4d reveal that Diox@Ru achieves maximum chemiluminescence under concurrent conditions of elevated ROS levels and alkaline conditions (high ROS & high pH). However, under acidic conditions, even with equimolar ROS concentrations, the system demonstrates a 5-fold reduction in chemiluminescent output, retaining merely ∼20% of its optimal value. These findings support a foundational framework for an AND logic gate of Diox@Ru, with its selective mechanism of activation that aligns with the distinct pathophysiological microenvironment of cancer cells, *i.e.*, both intracellular high ROS and mildly alkaline pH.

To assess the specificity of Diox@Ru activation toward H_2_O_2_, we coincubated the compound with other biomarkers commonly overexpressed in the tumor microenvironment, including glutathione (GSH, ∼10 mM intracellular),^50^ matrix metalloproteinase-1 (MMP-1, ∼500 ng/mL extracellular)^51^ and lactate dehydrogenase (LDH, ∼0.5 units/mL intracellular).^52^ Activation was quantified by measuring total chemiluminescence intensity, which directly correlates with the proportion of activated Diox@Ru. As shown in Figure 4e, robust chemiluminescence was observed only upon incubation with H_2_O_2_ for 2 h. In contrast, treatment with GSH, MMP-1, or LDH resulted in negligible activation, with signal levels comparable to the negative control, confirming the selective responsiveness of Diox@Ru to H_2_O_2_ at intracellular pH.

Having established the specificity of the chemiluminescence transfer in Diox@Ru in the presence of only the combination of ROS and pH, we next evaluated the ^1^O_2_-generation of Diox@Ru without an external light source, as this is a decisive factor for dark dynamic toxicity in cancer cells, as well as the broader applicability of our design concept for photocatalytic drugs. The singlet oxygen sensor 9,10-anthracenediyl-bis­(methylene)­dimalonic acid (ABDA) forms an endoperoxide of ABDA in the presence of ^1^O_2_, decreasing ABDA absorption and providing a valuable means of direct monitoring ^1^O_2_ production.^2,5,11^ Thus, we determined the ^1^O_2_-production of Diox@Ru by detecting the absorbance decrease of ABDA at 380 nm wavelength (Figure 4f). Notably, under dark conditions, Diox@Ru activated by H_2_O_2_ efficiently generates singlet oxygen, as evidenced by a 20% decrease in ABDA absorption at 380 nm. This reduction is approximately 10-fold greater than that observed with a physical mixture of Diox and Ru (2.1% decrease under identical conditions, Figure 4f), demonstrating the superior ^1^O_2_ generation capability of the Diox@Ru complex in the absence of light. The positive control, Ru shows higher decrease in ABDA absorption at 380 nm (65%) but only under light irradiation (470 nm LED lamp, Figure S9). Notably, Diox@Ru reveals negligible ^1^O_2_ production under H_2_O_2_-free conditions, confirming the essential role of H_2_O_2_ for ^1^O_2_-generation. Taken together, these results demonstrate that chemiluminescence is only activated in the presence of H_2_O_2_ and there is more efficient chemiluminescent energy transfer in the covalently bound Diox@Ru to achieve a higher amount of ^1^O_2_ without external excitation, compared to a mixture of Ru and Diox.

### Diox@Ru Activation in Cancer Cells and Controlled Cell Death through Singlet Oxygen Generation

Next, we evaluated whether the intracellular environment of cancer cells can autonomously activate Diox@Ru independent of an external light source. First, the cellular uptake of Diox@Ru was qualitatively evaluated in TNBC 4T1 (Figure S10) and lung carcinoma A549 cell lines (Figure S20) by applying confocal laser scanning microscopy analysis using red fluorescence of Ru, without applying supplementary fluorescent dyes. This experimental approach leverages the intrinsic photoluminescent properties of the ruthenium complex, which exhibits autofluorescence upon photoexcitation.^5,53^ The detection was performed at the characteristic emission wavelength of 615 nm. The distinct red fluorescence signals observed in the microscopic images reveal intracellular localization of Diox@Ru in both TNBC 4T1 and lung carcinoma A549 cell lines (Figures S10 and S20). Control experiments confirmed cellular uptake of both Ru and Me-Diox@Ru in TNBC 4T1 (Figure S10). Ru-associated red fluorescence verified the internalization of Ru, Diox@Ru, and Me-Diox@Ru, whereas Diox alone, lacking the intrinsic fluorescence, could not be directly visualized. To determine the subcellular localization of Diox@Ru, we performed colocalization analysis with mitochondrial and nuclear markers of 4T1 cells. As shown in Figure 5a, Diox@Ru (red) exhibits strong colocalization with mitochondria (yellow), while nuclei are stained in blue. Quantitative analysis revealed a Pearson’s correlation coefficient of 0.73 for Diox@Ru and mitochondria, indicating substantial overlap. In contrast, the correlation between Diox@Ru and nuclear staining was minimal (Pearson’s *R* ≈ 0.01), suggesting negligible nuclear accumulation. This preferential mitochondrial localization is consistent with previous studies on Ru polypyridyl complexes and supports their utility in targeting subcellular compartments for photodynamic applications.^2,54,55^

**Figure 5:**
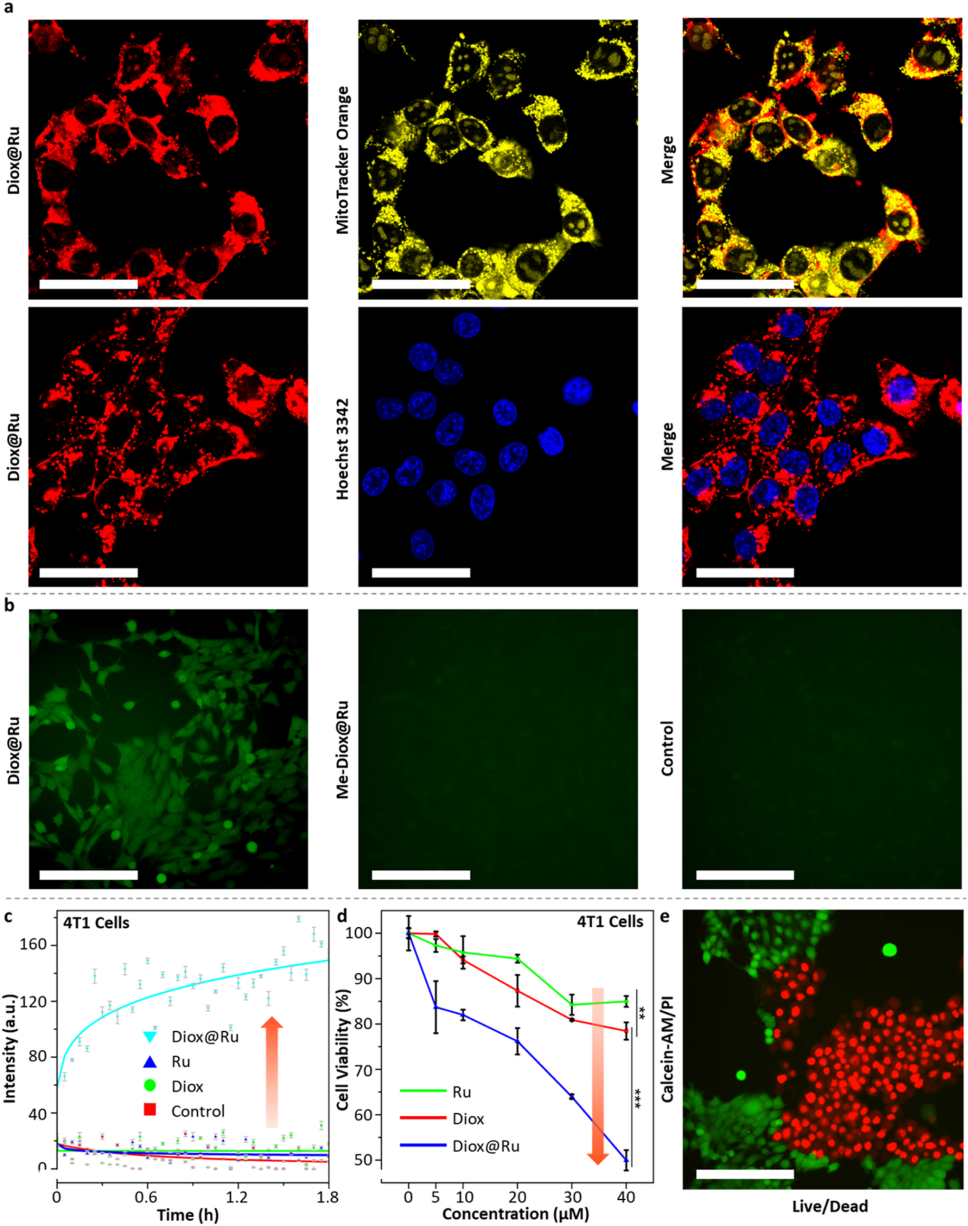
(a) Intracellular colocalization of Diox@Ru (**12**, 50 μM, incubation time 8 h) with mitochondria (Pearson’s *R* = 0.73) and nucleus (Pearson’s *R* = 0.01) in 4T1 cells. Red color corresponds to the fluorescence of Diox@Ru, yellow color reflects the MitoTracker Orange CMTMRos as mitochondria indicator and blue color reflects the Hoechst 33342 as nucleus indicator. Scale bar: 50 μm. (b) ROS production of Diox@Ru (**12**) and Me-Diox@Ru (50 μM, 8 h) in 4T1 cells. Green color reflects the 2′,7′-dichlorofluorescein diacetate as ROS indicator, Scale bar: 200 μm. Independent experiments (×3, Figure S13) (c) Intracellular chemiluminescence after incubation of 50 μM Diox@Ru (**12**), Ru (**11**) or Diox (**16**) in 4T1 cells (4 h, spectra obtained over 580–700 nm. The fitting line is shown as a solid line. Data presented as S.E.M, *n* = 3). (d) Cell viability of 4T1 cells (24 h, CellTiterGlo luminescent cell viability assay as an indicator). Data presented as S.E.M, *n* = 5. Statistical significance was calculated by ANOVA with a Tukey post hoc test. *p < 0.05, **p < 0.01, ***p < 0.001. (e) Live/dead cell viability of Diox@Ru (**12**, 50 μM, incubation time 8 h) in 4T1 cells. Green color reflects the Calcein O,O′-diacetate tetrakis­(acetoxymethyl) ester as live cells indicator and red color reflects the propidium iodide as dead cells indicator. Scale bar: 200 μm. Diox@Ru, Diox, and Ru were coincubated with cells containing <2% DMSO. *n* = technical replicates.

Next, intracellular ROS generation was assessed using Keyence microscope with the fluorogenic probe 2′,7′-dichlorodihydrofluorescein diacetate (DCFH-DA). Mechanistically, cell-permeable DCFH-DA undergoes passive diffusion into cells, where it is enzymatically hydrolyzed by endogenous esterases to yield a nonfluorescent intermediate. Subsequent oxidation of this intermediate by intracellular ROS converts it into the fluorescent derivative 2′,7′-dichlorofluorescein (DCF), which exhibits green fluorescence emission (λ_em_ = 525 nm) following excitation at 488 nm.^56^ This wavelength-specific fluorescence signal serves as a direct indicator of ROS production and activity within the cellular microenvironment. Thus, we investigated the intracellular ROS generation in TNBC 4T1 (Figures 5b and S12) and lung carcinoma A549 cells (Figure S21) by Diox@Ru, with Diox, Ru and Me-Diox@Ru as controls. Me-Diox@Ru serves as a control dioxetane of Diox@Ru where no chemiluminescence can be activated. The fluorescence images obtained using Keyence microscope show that Diox@Ru could produces ROS, whereas the control compounds Diox, Ru and Me-Diox@Ru are inactive (Figures S12 and S21). The increase of intracellular ROS in Diox@Ru, but not in Me-Diox@Ru and Ru, further corroborates the autonomous activation by H_2_O_2_ *in cellulo.* To gain deeper insight into the intracellular activation of Diox@Ru, we monitored the chemiluminescence kinetics within two distinct emission windows—460–500 nm corresponding to activated Diox, and 580–700 nm indicative of Ru emission. The presence of intracellular chemiluminescence in the 580–700 nm range, coupled with the disappearance of emission between 460 and 500 nm (Figures 5c, S14 and S21), clearly demonstrates that Diox@Ru is autonomously activated within 4T1 and A549 cancer cells, without the addition of exogenous H_2_O_2_, induction of ROS production, or reliance on external light stimulation.

Finally, the cytotoxic effects of Diox@Ru were evaluated in 4T1 and A549 cancer cell lines using a combination of viability assays, live/dead staining, and apoptosis profiling. Cell viability was quantified using the CellTiter-Glo luminescent assay, which measures intracellular ATP as a surrogate for metabolically active cells.^57^ Notably, the weak intrinsic chemiluminescence of Diox@Ru was several orders of magnitude lower than the ATP-driven signal of the assay, thereby ensuring specificity and accuracy. A concentration-dependent decrease in viability was observed, with IC_50_ values of approximately 40 μM for 4T1 cells (Figures 5d, and S14–16) and 30 μM for A549 cells (Figures S22 and S23). Statistical analyses confirmed a significantly higher cytotoxic effect for Diox@Ru compared to its individual components (*p* < 0.01 and 0.001), underscoring the enhanced efficacy of the conjugate.

To qualitatively assess cell viability and the underlying cytotoxicity mechanism, Calcein O, O′-diacetate tetrakis­(acetoxymethyl) ester (Calcein-AM)/propidium iodide (PI) dual staining was employed. This assay distinguishes live cells *via* intracellular esterase activity converting Calcein-AM into green-fluorescent calcein, while PI intercalates into DNA only upon loss of membrane integrity, marking controlled cell death by red fluorescence.^58^ Annexin V-FITC staining was used to detect early apoptotic cells by targeting phosphatidylserine exposed on the cell surface.^59^ Live/dead and cell apoptosis imaging confirmed that Diox@Ru induces cytotoxicity in both 4T1 (Figures 5e, S17 and S19) and A549 (Figures S25 and S26) cell lines. Importantly, these effects occurred in the absence of external light, indicating that Diox@Ru triggers controlled cell death through autonomous biochemical activation.

Next, we evaluated the effect of Diox@Ru on human keratinocyte cell lines derived from histologically normal skin cells (HaCaT cells). Cell viability assay after 24h incubation using CellTiter-Glo luminescent assay reveals that Diox@Ru exhibits negligible cytotoxicity toward HaCaT cells (Figure S27). Notably, a high cell viability at ∼80% was observed even at a high concentration of 100 μM, similar to the control Me-Diox@Ru. This result further corroborates the selective activation of Diox@Ru only in the unique intracellular chemical environment of cancer cells, *i.e.*, weakly alkaline pH and high ROS.

While 2D monolayer cultures are widely used for initial screening, they fail to replicate the complex architecture and signal gradients in the microenvironment of solid tumors.^60^ To better assess the application potential of Diox@Ru, we investigated its activity in 3D tumor spheroids derived from 4T1 cells. Confocal microscopy revealed effective cellular internalization of Diox@Ru and pronounced intracellular ROS generation, substantially greater than that observed for Diox or the Ru PS alone (Figure 6). To quantify cellular uptake of Diox@Ru, Diox, and Ru as well as associated ROS production in 3D tumor spheroids, we performed mean gray value analysis (total fluorescence intensity/area) on confocal images using ImageJ.^61–66^ Although light penetration limits absolute signal quantification in spheroids, all samples were imaged under identical conditions and processed using the same normalization protocols to ensure valid comparative analysis. This semiquantitative approach revealed that both Diox@Ru and Ru were efficiently internalized by cells, with Diox@Ru exhibiting markedly higher dark cytoxicity relative to Ru or Diox alone (Figure S28). Cell viability assays in 4T1 tumor spheroids (Figure S29) showed concentration-dependent cytotoxicity, with Diox@Ru reducing viability to ∼50% at 50 μM, supporting its enhanced therapeutic potential under physiologically relevant 3D conditions.

**Figure 6:**
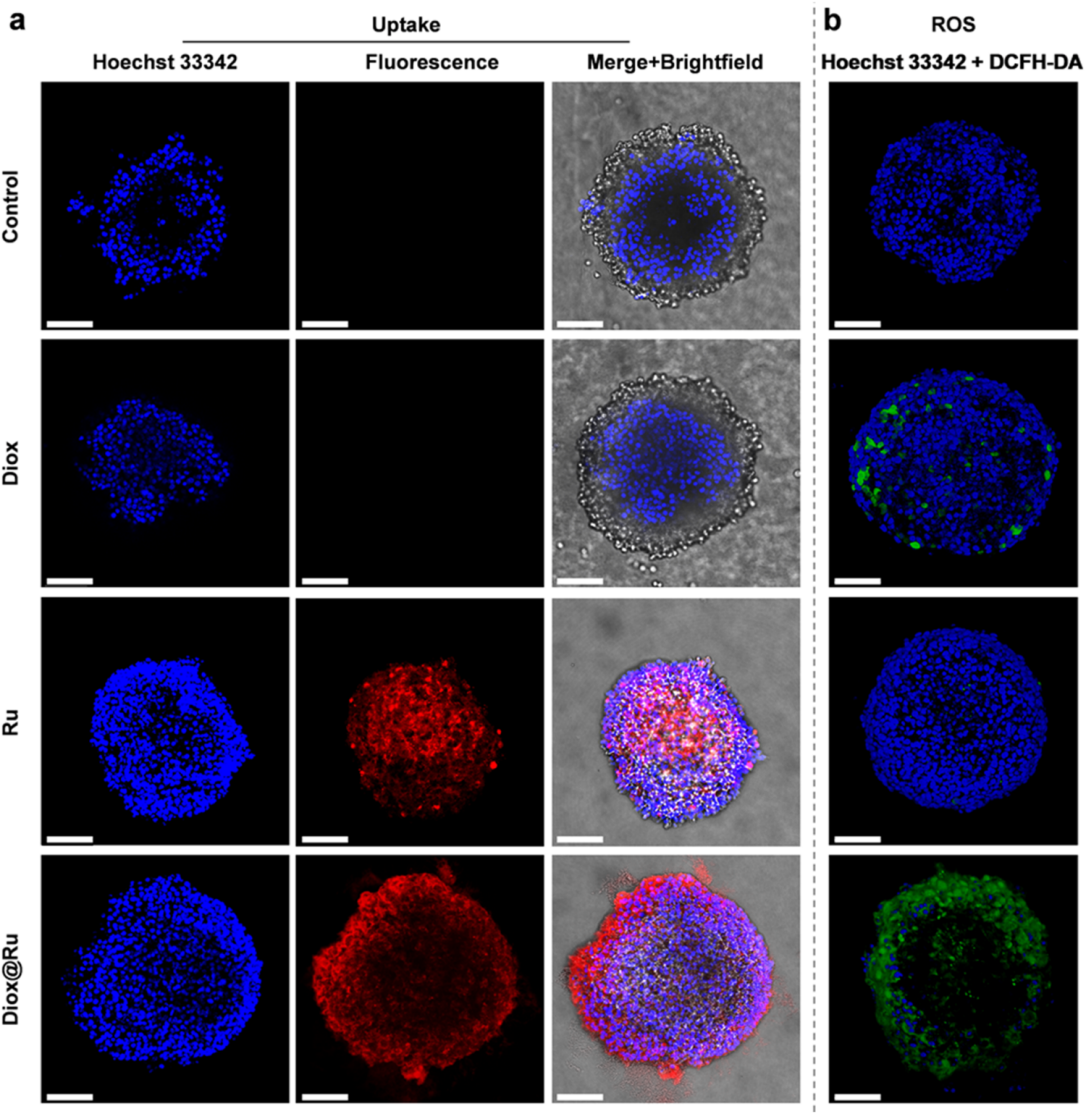
Cellular uptake and ROS production in 3D tumor spheroids of 4T1 cells. (a) Internalization and (b) ROS generation of Diox@Ru (**12**), Diox (**16**) and Ru (**11**) (50 μM, 1% DMSO, 24 h incubation, Hoechst 3342 as nuclear stain (blue color, λ_ex_ = 405 nm), red color represents the fluorescence of ruthenium (λ_ex_ = 460 nm), 2′, 7′-dichlorofluorescein diacetate as ROS indicator (green color, λ_ex_ = 488 nm)). Scale bar: 100 μm. The control group was evaluated under identical experimental conditions without the addition of chemical agents, *i.e.*, Diox@Ru, Diox or Ru. Independent experiments (×3, Figure S30).

These findings confirm that Diox@Ru retains photodynamic activity in the absence of an external light sources, even within the more physiologically relevant and diffusion-limited environment of 3D tumor spheroids. By enabling light-independent ROS production, Diox@Ru holds promise for overcoming the inherent limitations of conventional photodynamic therapy, particularly for the treatment of deep-seated tumors.

## Conclusions

In conclusion, we have developed a self-activating chemiluminescence-based photodynamic therapy (CLPDT) system, Diox@Ru, that integrates a Schaap’s dioxetane chemiluminescent scaffold with a boronic acid ester trigger and a ruthenium polypyridyl photosensitizer through direct covalent linkage. This molecular design constitutes an AND logic gate, ensuring precise activation exclusively under intracellular conditions characteristic of aggressive cancer phenotypes—namely, elevated reactive oxygen species and physiological pH. Upon selective activation, Diox@Ru undergoes intramolecular chemiexcitation, resulting in efficient energy transfer to the Ru photosensitizer and subsequent generation of cytotoxic singlet oxygen (^1^O_2_), thereby inducing apoptosis in 4T1 and A549 cancer cells without the need for external light irradiation.

The efficient intramolecular energy transfer, while the system remains inert under extracellular or nontumorigenic conditions, thereby minimizing off-target activation. Although future optimization of singlet oxygen yield may further enhance therapeutic efficacy under dark conditions, the current design, characterized by molecular simplicity, robust photophysical performance and selective intracellular ^1^O_2_ generation, highlights the potential of Diox@Ru as a robust platform for deep-tissue chemiluminescence-activated photodynamic therapy. More broadly, this work exemplifies the power of chemically encoded logic gates for achieving spatial control over molecular transformations in complex biological environments, offering a promising strategy for the development of tumor microenvironment-responsive therapeutic systems.

## Supplementary Material



## Notes

Details of instruments, materials, and methods (NMR, LCMS, HPLC, UV–vis), compounds synthesis, cell assays, sample preparation, workflow for microscopy, microscopy parameters, setup, and analysis, supporting figures and spectra of compounds (PDF)
