# Supplementary material for: Autonomous Activation of a Gated Chemiluminescent Photosensitizer Enables Targeted Photodynamic Therapy in Tumor Cells
Source: J Am Chem Soc. 2025 Jul 23;147(31):27822–34. doi: 10.1021/jacs.5c06761 (PMC12333359; doi:10.1021/jacs.5c06761)
Supplement: Supplementary file 1 [file ja5c06761_si_001.pdf]

Autonomous Activation of a Gated Chemiluminescent Photosensitizer Enables Targeted Photodynamic Therapy in Tumor Cells

Wenwu Peng [a], Tianjiao Zhou [a,b], Lifan Hu [a], Vivien Vankann [c], Toszka Bohn [c,d], Tobias Bopp [c,d,e,f,g], Seah Ling Kuan [a]* and Tanja Weil [a]*

[a] Max Planck Institute for Polymer Research, 55128 Mainz, Germany

[b] State Key Laboratory of Natural Medicines, Department of Pharmaceutics, China Pharmaceutical University, Nanjing 210009, China

[c] Institute of Immunology of the Research Center for Immunotherapy (FZI), University Medical Center, Johannes Gutenberg University Mainz, 55131 Mainz, Germany

[d] German Cancer Consortium (DKTK), 69120 Heidelberg, Germany

[e] University Cancer Center (UCT) Mainz, University Medical Center Mainz, 55131 Mainz, Germany

[f] Institute for Quantitative and Computational Biosciences (IQCB), 55128 Mainz, Germany

[g] Centre for Healthy Ageing, Johannes Gutenberg-University Mainz, 55128 Mainz, Germany

*Corresponding Author Email: weil@mpip-mainz.mpg.de (Tanja Weil); kuan@mpip-mainz.mpg.de (Seah Ling Kuan)

Supporting Information

Contents

1. Synthetic Schemes and Experimental Procedures 4

1.1 General Methods 4

1.2 Abbreviations 5

1.3 Synthesis of Diox, Ru and Diox@Ru 5

1.3.1 Compound 1[1] 5

1.3.2 Compound 2[1] 5

1.3.3 Compound 3[1] 6

1.3.4 Compound 4[1] 6

1.3.5 Compound 5 7

1.3.6 Compound 6[1] 8

1.3.7 Compound 7 8

1.3.8 Compound 8 9

1.3.9 Compound 9[2] 10

1.3.10 Compound 10 10

1.3.11 Compound 11-1[3] 11

1.3.12 Compound 11-2[4] 11

1.3.13 Compound 11-3 12

1.3.14 Compound 11 (Ru) 13

1.3.15 Compound 12 (Diox@Ru) 13

1.3.16 Compound 13 14

1.3.17 Compound 14 15

1.3.18 Compound 15 16

1.3.19 Compound 16 (Diox) 17

1.3.20 Compound 17 (Me-Diox@Ru) 17

1.4 Chemiluminescence Studies 19

1.5 $^1O_2$ Detection - ABDA 19

1.6 pH-Stability Studies 19

1.7 Light-Induced Decomposition Studies........20
1.8 Tumor Biomarker Specificity........20
1.9 Cell Culture........20
1.10 Cellular Internalization Assay........21
1.11 Intracellular Colocalization Assay........21
1.12 Intracellular Chemiluminescence Assay........22
1.13 Cell Viability - CellTiterGlo Luminescent Cell Viability Assay........22
1.14 Live/Dead Cell Viability Assay - Calcein-AM/PI........22
1.15 Intracellular ROS Assay - DCFH-DA........23
1.16 Cell Apoptosis Assay - Annexin V-FITC........23
1.17 Tumor Spheroids........24
1.18 Cell Viability of HaCaT Cells........24
1.19 Statistical Analysis of Cellular Experiments........24

2. Supporting Figures........26

3. Spectra of Compounds (^{1}H-NMR, ^{13}C-NMR, LC-MS)........44

4. Author contribution........62

5. References:........63

1. Synthetic Schemes and Experimental Procedures

1.1 General Methods

All chemicals and solvents were purchased from commercial sources and used without further purification. Thin layer chromatography (TLC) was used for visualizing and monitoring compounds by irradiation with UV light: 0.2 mm silica gel with fluorescent indicator (MACHEREY-NAGEL ALUGRAM SIL G/UV254). Column chromatography with silica gel (MACHEREY-NAGEL silica 60 M, 0.04-0.063 mm) as a stationary phase was used for purification. High-performance liquid chromatography - mass spectrometry (HPLC-MS) was used for reaction monitoring and mass analysis: Shimadzu LC-MS 2020, Phenomenex Kinetex 2.6 µm EVO C18 100 Å LC 50 × 2.1 mm as the column, H_2O and ACN with 0.1% formic acid as mobile phase. Preparative high-performance liquid chromatography (HPLC) was used for purification: Shimadzu Nexera, Phenomenex Kinetex 5µm EVO C18 100 Å, 30 × 150 mm as the column, 25 mL/min flowrate, H_2O and ACN with 0.1% trifluoroacetic acid as mobile phase. The ESI-HRMS spectra were recorded using a SYNAPT G2-Si mass spectrometer (Waters Corp., Manchester, UK, calibrated by clusters of sodium iodide in the mass range of 100-2000 m/z). The measurements were carried out at a capillary voltage of 2-3 kV, sampling cone 20 V and source offset 50 V, and a source temperature of 100 °C. Nitrogen was used as the desolvation gas at a total flow of 500 L h^{-1}. Absorbance, fluorescence and chemiluminescence spectra were recorded on Tecan Spark 20M microplate reader. Nuclear Magnetic Resonance Spectroscopy (NMR): AVANCE NEO 400 MHz NMR spectrometer for ^{1}H-NMR and AVANCE NEO 101 MHz NMR spectrometer for ^{13}C-NMR at 298 K. Images were recorded on Confocal Laser Scanning Microscopy (STELLARIS 8 Leica DMi8 microscope) and Keyence Microscope BZX800.

1.2 Abbreviations

EA: ethyl acetate, Hex: hexane, TLC: thin layer chromatography, DCM: dichloromethane, THF: tetrahydrofuran, ACN: acetonitrile, DMF: dimethylformamide, ABDA: 9,10-anthracenediyl-bis(methylene)dimalonic acid, PI: propidium iodide, TFA: trifluoroacetic acid, FBS: fetal bovine serum, ROS: reactive oxygen species, PFA: paraformaldehyde, DCFH-DA: 2', 7'-dichlorofluorescein diacetate, Calcein-AM: calcein O, O'-diacetate tetrakis(acetoxymethyl) ester.

1.3 Synthesis of Diox, Ru and Diox@Ru

1.3.1 Compound **1**[1]

2-chloro-3-hydroxybenzaldehyde (4000 mg, 25.5 mmol) was dissolved in 26 mL dry methanol. Trimethyl orthoformate (3523 mg, 33.2 mmol) and hydrochloric acid (0.086 mL) were added to the resulting solution. Then, the reactive solution was stirred at room temperature and monitored by TLC. Upon completion, the mixture was diluted by adding ethyl acetate and extracted with 0.01 M $NaHCO_3$ solution. The organic phase was dried over Na_2SO_4 and concentrated under reduced pressure. The crude product was purified by column chromatography (EA:Hex=1:4) to afford 5064 mg (98 % yield) of colorless oil. ^{1}H NMR (400 MHz, DMSO-d6) δ 10.17 (s, 1H), 7.14 (t, J = 7.9 Hz, 1H), 6.97 (ddd, J = 9.5, 7.8, 1.6 Hz, 2H), 5.51 (s, 1H), 3.27 (s, 6H). MS (ESI+): *m/z* calc. for $C_9H_{11}ClO_3$: 202.5; found: 171.0 $[M-CH_3O^-]^+$.

1.3.2 Compound **2**[1]

2-chloro-3-(dimethoxymethyl)phenol (**1**, 5140 mg, 25.4 mmol) and imidazole (3453 mg, 50.7 mmol) were dissolved in 42 mL dry DCM. Then t-butyl-dimethyl-chlorosilane (4590 mg, 30.5 mmol) was added in batches to the solution and a white precipitate should be formed. The mixture

was stirred at room temperature and monitored by TLC. Upon completion, the mixture was filtered and washed with DCM. The resulting solution was dried over Na_2SO_4 and concentrated under reduced pressure. The crude product was purified by column chromatography (EA:Hex=1:9) to afford 7638 mg (95 % yield) of colorless oil. ^{1}H NMR (400 MHz, DMSO-d6) δ 7.24 (t, J = 7.9 Hz, 1H), 7.16 (dd, J = 7.8, 1.7 Hz, 1H), 7.01 (dd, J = 8.0, 1.6 Hz, 1H), 5.53 (s, 1H), 3.27 (s, 6H), 1.00 (s, 9H), 0.22 (s, 6H). MS (ESI+): *m/z* calc. for $C_{15}H_{25}ClO_3Si$: 316.5; found: 285.2 $[M-CH_3O^-]^+$.

1.3.3 Compound **3**[1]

2-chloro-1-(dimethoxymethyl)-3-[[(1,1-dimethylethyl)dimethylsilyl]oxy]benzene (**2**, 6890 mg, 21.7 mmol) and trimethyl phosphite (3237.3 mg, 26.1 mmol) were dissolved in 138 mL dry DCM. Then the solution was cooled down to 0 °C and titanium (IV) chloride (4948.8 mg, 26.1 mmol) was added dropwise. The solution turned yellow and was monitored by TLC. Upon completion, the solution was poured into a saturated aqueous solution of $NaHCO_3$ at 0 °C and stirred for 2 minutes. The DCM was added to separate the phases and the organic phase was dried over Na_2SO_4. The crude product was concentrated under reduced pressure and purified by column chromatography (Hex:EA=2:3) to afford 8227 mg (96 % yield) of colorless oil. ^{1}H NMR (400 MHz, DMSO-d6) δ 7.30 (t, J = 7.9 Hz, 1H), 7.16 (ddd, J = 7.9, 2.4, 1.6 Hz, 1H), 7.01 (dt, J = 8.1, 1.7 Hz, 1H), 5.08 (d, J = 15.2 Hz, 1H), 3.67 (d, J = 10.6 Hz, 3H), 3.54 (d, J = 10.5 Hz, 3H), 3.25 (s, 3H), 1.00 (s, 9H), 0.23 (s, 6H). MS (ESI+): *m/z* calc. for $C_{16}H_{28}ClO_5PSi$: 394.5; found: 789.3 $[2M+H]^+$.

1.3.4 Compound **4**[1]

Dimethyl ((3-((tert-butyldimethylsilyl)oxy)-2-chlorophenyl)(methoxy)methyl)phosphonate (**3**,

8259 mg, 20.9 mmol) was dissolved in 62 mL anhydrous THF under N_2 atmosphere and then cooled down to -78°C. Lithium diisopropylamide (2.0 M in THF, 2689 mg, 25.1 mmol, 12.6 mL) was added dropwise and the solution was stirred for 10 minutes and turned red. 2-adamantanone was dissolved in 42 mL anhydrous THF and the solution was added into the above mixture dropwise. The solution was stirred for 5 minutes at -78°C and turned yellow. Then, the reactive solution was warmed to room temperature and monitored by TLC. Upon completion, the mixture was diluted with EA and extracted with brine. The organic phase was dried over Na_2SO_4 and concentrated under reduced pressure. The crude product was purified by column chromatography (Hex:EA= 90:10) to afford 7445 mg (85 % yield) of white solid. ^{1}H NMR (400 MHz, CD_2Cl_2) δ 7.12 (dd, J = 8.2, 7.5 Hz, 1H), 6.88 (ddd, J = 9.3, 7.8, 1.6 Hz, 2H), 3.27 (s, 3H), 3.24 – 3.20 (m, 1H), 2.06 – 2.00 (m, 1H), 1.96 – 1.79 (m, 9H), 1.74 – 1.64 (m, 3H), 1.04 (s, 9H), 0.23 (s, 6H). MS (ESI+): *m/z* calc. for $C_{24}H_{35}ClO_2Si$: 418.5; found: 419.3 $[M+H]^+$.

1.3.5 Compound **5**

(3-(((1r,3r,5R,7S)-adamantan-2-ylidene)(methoxy)methyl)-2-chlorophenoxy)(tert-butyl)dimethyl silane (**4**, 420 mg, 1 mmol), Bis(pinacolato)diborane (457 mg, 1.8 mmol), 4,4’-Bis(1,1-dimethylethyl)-2,2’-bipyridine (10.7 mg, 0.04 mmol) and Bis[(1,2,5,6-η)-1,5-cyclooctadiene]di-μ-methoxydiiridium (I) (13.3 mg, 0.02 mmol) were dissolved in dry THF (5 mL) in sealed tube. Then, the mixture was stirred at 80 °C and monitored by TLC. The solution was diluted with EA upon completion and concentrated under reduced pressure. The crude product was purified by column chromatography (EA:Hex=3:7) to afford 523 mg (96 % yield) of colorless solid. ^{1}H NMR (400 MHz, CD_2Cl_2) δ 7.24 (q, J = 1.5 Hz, 2H), 3.26 (s, 3H), 3.23 (q, J = 3.4 Hz, 1H), 2.03 – 1.99 (m, 1H), 1.96 – 1.79 (m, 9H), 1.73 – 1.67 (m, 3H), 1.33 (s, 12H), 1.04 (s, 9H), 0.24 (s, 6H). ^{13}C

NMR (101 MHz, CD_2Cl_2) δ 151.71, 140.54, 136.10, 131.22, 130.74, 130.06, 126.14, 84.41, 56.92, 39.45, 39.33, 38.98, 38.89, 37.58, 33.35, 30.08, 29.03, 28.90, 25.89, 25.08, 25.06, 18.68, -4.28, -4.31. MS (ESI+): *m/z* calc. for $C_{30}H_{46}BClO_4Si$: 544.3; found: 545.4 $[M+H]^+$.

1.3.6 Compound **6**[1]

N-hydroxysuccinimide (115 mg, 1 mmol), 4-bromomethyl benzoic acid (215 mg, 1 mmol) and N-(3-Dimethylaminopropyl)-N′-ethylcarbodiimid-hydrochlorid (192 mg, 1 mmol) were dissolved in 10 mL of dry DCM. The solution was stirred at room temperature and monitored by TLC. Upon completion, the mixture was extracted with a saturated aqueous solution of $NaHCO_3$ and NaCl. The organic phase was dried over Na_2SO_4 and concentrated under reduced pressure. The crude product was purified by recrystallization using EA/Hex (7/3) to afford 281 mg (90 % yield) of white solid. ^{1}H NMR (400 MHz, $CDCl_3$) δ 8.14 – 8.09 (m, 2H), 7.57 – 7.51 (m, 2H), 4.51 (s, 2H), 2.91 (s, 4H). MS (ESI-): *m/z* calc. for $C_{12}H_{10}BrNO_4$: 312.1; found: 311.2 $[M-H]^-$.

1.3.7 Compound **7**

(3-(((1r,3r,5R,7S)-adamantan-2-ylidene)(methoxy)methyl)-2-chloro-5-(4,4,5,5-tetramethyl-1,3,2-dioxaborolan-2-yl)phenoxy)(tert-butyl)dimethylsilane (**5**, 550 mg, 1 mmol), 2,5-dioxopyrrolidin-1-yl 4-(bromomethyl)benzoate (**6**, 343 mg, 1.1 mmol), tetrakis (triphenylphosphine) palladium(0) (116 mg, 0.1 mmol) and potassium carbonate (351 mg, 2.5 mmol) were added in the sealed tube and exchanged the air of the tube by N_2. Anhydrous 1, 4-dioxane (7.5 mL) was added in the above tube and the mixture was stirred at 120°C. The reaction was monitored by TLC. Upon completion, the solids were filtered and the solution was concentrated under reduced pressure. The crude product was purified by column chromatography (EA:Hex=1:1) to afford 260 mg (40 % yield) of

white solid. ^{1}H NMR (400 MHz, $CDCl_3$) δ 8.05 (d, J = 8.4 Hz, 2H), 7.29 (d, J = 8.3 Hz, 2H), 6.70 (d, J = 2.2 Hz, 1H), 6.65 (d, J = 2.1 Hz, 1H), 3.98 (s, 2H), 3.29 (s, 3H), 3.25 (d, J = 3.1 Hz, 1H), 2.90 (s, 4H), 2.03 – 2.00 (m, 1H), 1.95 – 1.91 (m, 2H), 1.89 (d, J = 3.0 Hz, 2H), 1.84 – 1.77 (m, 4H), 1.71 (d, J = 5.1 Hz, 2H), 1.62 – 1.49 (m, 2H), 1.01 (s, 9H), 0.18 (s, 6H). ^{13}C NMR (101 MHz, $CDCl_3$) δ 171.29, 169.40, 161.86, 152.09, 148.54, 140.13, 138.23, 136.33, 131.04, 130.94, 129.32, 125.43, 124.93, 123.29, 120.94, 60.53, 57.06, 41.49, 39.22, 39.11, 38.73, 37.72, 37.29, 36.66, 34.70, 33.05, 31.16, 29.71, 28.52, 28.41, 25.82, 21.19, 18.51, 14.34, -4.23, -4.26. MS (ESI+): *m/z* calc. for $C_{36}H_{44}ClNO_6Si$: 649.5; found: 650.3 $[M+H]^+$.

1.3.8 Compound **8**

2,5-dioxopyrrolidin-1-yl 4-(3-(((1r,3r,5R,7S)-adamantan-2-ylidene)(methoxy)methyl)-5-((tert-butyldimethylsilyl)oxy)-4-chlorobenzyl)benzoate (**7**, 240 mg, 0.36 mmol) was dissolved in anhydrous THF (12 mL). The solution was cooled down to 0 °C and then tetrabutylammonium fluoride (1.0 M in THF, 181 mg, 0.69 mmol, 0.69 mL) was added to the solution by dropwise. The solution was stirred at 0 °C and monitored by TLC. Upon completion, the mixture was diluted with EA and washed with NH_4Cl (1 M) solution and saturated NaCl solution. The organic phase was dried over Na_2SO_4 and concentrated under reduced pressure. The crude product was purified by column chromatography (EA:Hex=1:2) to afford 174 mg (90 % yield) of colorless oil. ^{1}H NMR (400 MHz, $CDCl_3$) δ 8.06 (d, J = 8.3 Hz, 2H), 7.31 (d, J = 8.5 Hz, 2H), 6.80 (d, J = 2.1 Hz, 1H), 6.66 (d, J = 2.1 Hz, 1H), 5.69 (s, 1H), 4.00 (s, 2H), 3.29 (s, 3H), 3.25 (d, J = 3.1 Hz, 1H), 2.90 (s, 4H), 2.07 (t, J = 3.3 Hz, 1H), 1.96 – 1.89 (m, 4H), 1.80 (d, J = 8.2 Hz, 4H), 1.72 (d, J = 10.4 Hz, 2H), 1.61 (d, J = 13.6 Hz, 2H). ^{13}C NMR (101 MHz, $CDCl_3$) δ 169.41, 161.85, 151.92, 148.30, 139.73, 139.35, 135.29, 132.21, 131.08, 130.98, 130.23, 129.61, 129.43, 129.11, 128.00, 124.54,

124.47, 123.35, 116.07, 116.03, 60.55, 57.30, 41.57, 39.16, 37.21, 33.01, 29.79, 28.41, 25.82, 25.81. MS (ESI+): *m/z* calc. for $C_{30}H_{30}ClNO_6$: 535.5; found: 536.3 $[M+H]^+$.

1.3.9 Compound **9**[2]

(4-(4,4,5,5-Tetramethyl-1,3,2-dioxaborolan-2-yl)phenyl)methano (300 mg, 1.28 mmol) and NaI (576 mg, 3.85 mmol) were dissolved in ACN (9 mL) and cooled down to 0 °C. Then tert-butylchlorodiphenylsilane (418 mg, 3.85 mmol) was added to the reaction mixture by dropwise at 0 °C. The solution was stirred at room temperature and monitored by TLC. Upon completion, the solution was evaporated under reduced pressure and the crude product was diluted with EA. The resulting mixture was washed with a saturated $Na_2S_2O_3$ aqueous solution. The organic phase was dried over Na_2SO_4 and concentrated under reduced pressure. The crude product was purified by column chromatography (EA:Hex=1:10) and recrystallization with EA to afford 418 mg (95 % yield) of white solid. ^{1}H NMR (400 MHz, $CDCl_3$) δ 7.75 – 7.72 (m, 2H), 7.40 – 7.35 (m, 2H), 4.45 (s, 2H), 1.34 (s, 12H). MS (ESI+): *m/z* calc. for $C_{13}H_{18}BIO_2$: 344.0; found: 345.0 $[M+H]^+$.

1.3.10 Compound **10**

2,5-dioxopyrrolidin-1-yl 4-(3-(((1r,3r,5R,7S)-adamantan-2-ylidene)(methoxy)methyl)-4-chloro-5-hydroxybenzyl)benzoate (**8**, 300 mg, 0.56 mmol) and potassium carbonate (85 mg, 0.62 mmol) were dissolved in dry DMF (10 mL). The solution was stirred at room temperature for 10 minutes and then 2-(4-(iodomethyl)phenyl)-4,4,5,5-tetramethyl-1,3,2-dioxaborolane (**9**, 250 mg, 0.73 mmol) was added into the above mixture. The solution was stirred at room temperature and monitored by TLC. Upon completion, the mixture was diluted with EA and washed with 1M NH_4Cl as well as brine. The organic phase was dried over Na_2SO_4 and concentrated under reduced

pressure. The crude product was purified by column chromatography (EA:Hex=1:1) to afford 358 mg (85 % yield) of white solid. ^{1}H NMR (400 MHz, $CDCl_3$) δ 8.07 – 8.03 (m, 2H), 7.84 – 7.80 (m, 2H), 7.48 – 7.44 (m, 2H), 7.27 (d, J = 1.8 Hz, 1H), 7.25 (s, 1H), 6.73 – 6.68 (m, 2H), 5.11 (s, 2H), 4.00 (s, 2H), 3.31 (s, 3H), 3.26 (d, J = 3.4 Hz, 1H), 2.95 – 2.84 (m, 4H), 1.97 – 1.88 (m, 6H), 1.84 – 1.78 (m, 3H), 1.71 (d, J = 11.6 Hz, 2H), 1.61 (s, 2H), 1.34 (s, 12H). ^{13}C NMR (101 MHz, $CDCl_3$) δ 169.38, 161.82, 154.61, 148.35, 139.89, 139.55, 138.39, 136.39, 135.16, 131.48, 131.20, 131.08, 129.39, 129.32, 126.52, 124.97, 123.36, 122.42, 113.95, 83.97, 71.01, 60.53, 57.22, 41.79, 39.25, 39.16, 38.78, 38.69, 37.27, 33.02, 29.82, 29.75, 28.52, 28.39, 25.82, 25.01, 21.19, 14.33. MS (ESI+): *m/z* calc. for $C_{43}H_{47}BClNO_8$: 751.3; found: 752.4 $[M+H]^+$.

1.3.11 Compound **11-1**[3]

1,10 phenanthroline-5,6-dione (200 mg, 0.95 mmol) and 4-carboxybenzaldehyde (143 mg, 0.95 mmol) were dissolved in acetic acid (5 mL). The mixture was heated to 80 °C and stirred for 30 minutes. Ammonium acetate (1460 mg, 19 mmol) was added to the above mixture and stirred at 120 °C for 3 hours. The mixture was cooled down to room temperature. Then the product was filtered and washed with water, acetone and ether to afford 291 mg (90 % yield) of light-yellow solid. ^{1}H NMR (400 MHz, DMSO-d6) δ 9.05 (dd, J = 4.3, 1.8 Hz, 2H), 8.96 (dd, J = 8.1, 1.8 Hz, 2H), 8.41 (d, J = 8.4 Hz, 2H), 8.17 (d, J = 8.4 Hz, 2H), 7.84 (dd, J = 8.1, 4.3 Hz, 2H). MS (ESI+): *m/z* calc. for $C_{20}H_{12}N_4O_2$: 340.0; found: 341.1 $[M+H]^+$.

1.3.12 Compound **11-2**[4]

Ethylenediamine (1159 mg, 19.3 mmol) was dissolved in dry DCM (7 mL) and the solution was cooled down to 0 °C. 9-fluorenylmethyl-succinimidyl carbonate (651 mg, 1.93) was dissolved in

dry DCM (6 mL) and added into the above solution dropwise. Upon completion, the white solid was filtered out and the organic phase solution was extracted with H_2O. The resulting solution was concentrated under reduced pressure and to afford 272 mg (50 % yield) of white solid. ^{1}H NMR (400 MHz, DMSO-d6) δ 8.43 (s, 3H), 8.06 (s, 1H), 7.89 (dt, J = 7.6, 1.0 Hz, 1H), 7.70 (d, J = 7.5 Hz, 1H), 7.51 – 7.30 (m, 3H), 4.33 (d, J = 6.8 Hz, 1H), 3.41 (s, 4H), 3.25 (q, J = 6.3 Hz, 1H), 3.09 (s, 2H), 2.84 (q, J = 6.0 Hz, 1H). MS (ESI+): *m/z* calc. for $C_{17}H_{18}N_2O_2$: 282.0; found: 283.2 $[M+H]^+$.

1.3.13 Compound **11-3**

4-(1H-imidazo[4,5-f][1,10]phenanthrolin-2-yl)benzoic acid (**11-1**, 400 mg, 1.18 mmol) was dissolved in dry DMF and cooled down to 0 °C. (9H-fluoren-9-yl)methyl (2-aminoethyl)carbamate (**11-2**, 380 mg, 1.18 mmol) was added to the solution and then N-(3-Dimethylaminopropyl)-N′-ethylcarbodiimid-hydrochlorid (560 mg, 2.92 mmol) and 4-dimethylaminopyridine (72 mg, 0.59 mmol) were added to the above mixture. It was stirred at 0 °C for a further 4 hours and the solvent was removed under reduced pressure. H_2O was added to obtain precipitation of a solid and it was washed with diethyl ether. Then the precipitation was isolated by centrifugation and dried to afford 557 mg (78 % yield) of yellow solid. ^{1}H NMR (400 MHz, DMSO-d6) δ 14.25 (s, 1H), 9.12 – 8.80 (m, 4H), 8.67 (s, 1H), 8.44 (t, J = 8.5 Hz, 2H), 8.32 – 8.19 (m, 1H), 8.09 (dd, J = 20.2, 8.1 Hz, 2H), 7.97 – 7.63 (m, 6H), 7.41 (ddt, J = 35.6, 28.3, 6.7 Hz, 3H), 4.28 (dd, J = 35.5, 7.1 Hz, 1H), 3.17 (s, 2H), 3.02 (dt, J = 13.3, 6.4 Hz, 2H), 2.89 (s, 1H), 2.73 (d, J = 3.8 Hz, 2H). ^{13}C NMR (101 MHz, DMSO-d6) δ 162.32, 158.42, 149.95, 147.60, 143.91, 140.74, 139.06, 132.15, 130.71, 127.95, 127.62, 127.07, 126.06, 125.18, 123.71, 120.13, 106.95, 54.53, 46.74, 42.10, 35.79. MS (ESI+): *m/z* calc. for $C_{37}H_{28}N_6O_3$: 604.0; found: 605.3 $[M+H]^+$.

1.3.14 Compound **11 (Ru)**

A mixture of cis-[Ru(bpy)$_2$Cl$_2$]·2H_2O (96 mg, 0.189 mmol) and (9H-fluoren-9-yl)methyl (2-(4-(1H-imidazo[4,5-f][1,10]phenanthrolin-2-yl)benzamido)ethyl)carbamate (114 mg, 0.189 mmol) in 15 mL ethanol and H_2O (2:1, v/v) was refluxed under N_2 for 2 hours to give a clear red solution. Upon completion, the solution was concentrated under reduced pressure to obtain a red solid. Without further purification, the solid was dissolved in 11 mL DMF/EtOH (10/1, v/v) and diethylamine (2.5 mL) was added to the above solution. The reaction was monitored by LCMS. Upon completion, the solution was concentrated under reduced pressure and purified by HPLC to afford 214 mg (60 % yield) of red solid. HPLC Method: Shimadzu Nexera, Phenomenex Kinetex 5um EVO C18 100 Å, 30 × 150 mm as the column, 25 mL/min flow rate, H_2O and ACN with 0.1% trifluoroacetic acid as mobile phase. The gradient started with 0% CH_3CN in H_2O (0.1% CF_3COOH) and this solvent ratio was kept for 1 min, after which the CH_3CN content was increased to 100% in 11 min. The product eluted from the column after 7.6 min. ^1H NMR (400 MHz, DMSO-d6) δ 9.15 (dd, J = 8.3, 1.3 Hz, 2H), 8.88 (ddt, J = 15.3, 8.4, 1.0 Hz, 5H), 8.46 (d, J = 8.6 Hz, 2H), 8.22 (td, J = 7.9, 1.5 Hz, 2H), 8.17 – 8.07 (m, 6H), 8.01 – 7.93 (m, 4H), 7.85 (dt, J = 5.4, 1.2 Hz, 2H), 7.64 – 7.57 (m, 4H), 7.35 (ddd, J = 7.3, 5.7, 1.3 Hz, 2H), 3.57 (q, J = 6.1 Hz, 2H), 3.05 (q, J = 6.0 Hz, 2H). ^{13}C NMR (101 MHz, DMSO-d6) δ 166.21, 158.32, 158.00, 156.80, 156.58, 151.79, 151.50, 151.40, 150.06, 145.26, 138.00, 137.85, 135.29, 131.90, 130.55, 128.28, 127.91, 127.77, 126.43, 126.38, 124.50, 124.42. LC retention time: 3.0 min. MS (ESI+): m/z calc. for $C_{42}H_{34}N_{10}ORu^{2+}$: 794.9; found: 795.3 $[M-H^+-2Cl^-]^+$.

1.3.15 Compound **12 (Diox@Ru)**

2,5-dioxopyrrolidin-1-yl 4-(3-(((1r,3r,5R,7S)-adamantan-2-ylidene)(methoxy)methyl)-4-chloro-

5-((4-(4,4,5,5-tetramethyl-1,3,2-dioxaborolan-2-yl)benzyl)oxy)benzyl)benzoate (**10**, 30 mg, 0.04 mmol) and a catalytic amount of methylene blue (0.7 mg) were dissolved in 5 mL dry DCM. Oxygen was bubbled through the mixture while irradiating with yellow light. The reaction was monitored by TLC. Upon completion, the solvent was concentrated under reduced pressure and the crude product was passed through a silica gel column (EA : Hex = 1:1) to remove methylene blue. The solution was concentrated and dried to obtain a white solid and it was dissolved in 3 mL dry DMF. Then Ru (**11**, 45 mg, 0.04 mmol) and trimethylamine (0.02 mL) were added to the above mixture. The reaction was stirred at room temperature and monitored by LCMS. Upon completion, the solution precipitates as a solid when added dropwise to cold diethyl ether. The product was dried to afford 51 mg (88 % yield) of red solid. ^{1}H NMR (400 MHz, DMSO-d6) δ 14.66 (s, 1H), 9.12 (d, J = 8.2 Hz, 2H), 8.87 (dd, J = 15.5, 8.2 Hz, 5H), 8.60 (s, 1H), 8.41 (d, J = 8.1 Hz, 2H), 8.23 (dd, J = 7.9, 1.5 Hz, 2H), 8.13 – 8.07 (m, 6H), 7.94 (s, 2H), 7.85 (d, J = 5.6 Hz, 2H), 7.80 (d, J = 7.9 Hz, 2H), 7.68 (d, J = 7.8 Hz, 2H), 7.61 (dd, J = 7.5, 3.2 Hz, 4H), 7.46 (d, J = 7.7 Hz, 2H), 7.35 (dt, J = 7.8, 3.4 Hz, 3H), 7.30 (d, J = 8.0 Hz, 1H), 5.25 (d, J = 5.2 Hz, 2H), 4.08 (s, 2H), 3.49 (s, 4H), 3.07 (s, 4H), 2.83 (s, 1H), 2.23 (d, J = 12.5 Hz, 1H), 1.87 (s, 1H), 1.62 (t, J = 21.8 Hz, 10H), 1.41 (d, J = 12.8 Hz, 2H), 1.29 (s, 12H). LC retention time: 7.5 min. MS (ESI+): m/z calc. for $C_{81}H_{76}BClN_{10}O_8Ru^{2+}$: 1463.1; found: 1463.7 $[M-H^+-2Cl^-]^+$. High resolution-ESI (HR-ESI): m/z calc. for $C_{81}H_{76}BClN_{10}O_8Ru^{2+}$: 731.5172; found 731.5164 $[M-2H^+-2Cl^-]^{2+}$.

1.3.16 Compound **13**

(3-(((1r,3r,5R,7S)-adamantan-2-ylidene)(methoxy)methyl)-2-chloro-5-(4,4,5,5-tetramethyl-1,3,2-dioxaborolan-2-yl)phenoxy)(tert-butyl)dimethylsilane (**5**, 550 mg, 1 mmol), Methyl 4-(bromomethyl)benzoate (252 mg, 1.1 mmol), tetrakis (triphenylphosphine) palladium(0) (116 mg,

0.1 mmol) and potassium carbonate (351 mg, 2.5 mmol) were added in the sealed tube and exchanged the air of the tube by N_2. Anhydrous 1, 4-dioxane (7.5 mL) was added in the above tube and the mixture was stirred at 120°C. The reaction was monitored by TLC. Upon completion, the solids were filtered and the solution was concentrated under reduced pressure. The crude product was purified by column chromatography (EA:Hex=1:20) to afford 284 mg (50 % yield) of white solid. ^{1}H NMR (400 MHz, $CDCl_3$) δ 7.98 – 7.94 (m, 2H), 7.23 – 7.19 (m, 2H), 6.70 (d, J = 2.1 Hz, 1H), 6.63 (d, J = 2.1 Hz, 1H), 3.95 (s, 2H), 3.90 (s, 3H), 3.29 (s, 3H), 3.24 (t, J = 3.1 Hz, 1H), 2.02 (t, J = 3.1 Hz, 1H), 1.95 – 1.87 (m, 5H), 1.85 – 1.76 (m, 4H), 1.73 – 1.66 (m, 2H), 1.60 (s, 1H), 1.00 (s, 9H), 0.16 (s, 6H). ^{13}C NMR (101 MHz, $CDCl_3$) δ 167.15, 151.97, 146.06, 140.21, 138.85, 136.14, 130.75, 130.00, 129.73, 129.21, 129.08, 128.88, 128.38, 125.43, 124.65, 120.92, 60.53, 57.01, 52.19, 41.32, 39.20, 39.11, 38.74, 38.70, 37.30, 33.03, 29.70, 28.55, 28.42, 25.82, 18.50, -4.27, -4.30. MS (ESI+): m/z calc. for $C_{33}H_{43}ClO_4Si$: 566.5; found: 567.4 $[M+H]^+$.

1.3.17 Compound **14**

Methyl 4-(3-(((1r,3r,5R,7S)-adamantan-2-ylidene)(methoxy)methyl)-5-((tert-butyldimethylsilyl)-oxy)-4-chlorobenzyl)benzoate (**13**, 380 mg, 0.67 mmol) was dissolved in anhydrous THF (27 mL). Then tetrabutylammonium fluoride (1.0 M in THF, 193 mg, 0.74 mmol, 0.74 mL) was added to the solution by dropwise. The solution was stirred at room temperature and monitored by TLC. Upon completion, the mixture was diluted with EA and washed with NH_4Cl (1 M) solution and saturated NaCl solution. The organic phase was dried over Na_2SO_4 and concentrated under reduced pressure. The crude product was purified by column chromatography (EA:Hex=1:4) to afford 300 mg (99 % yield) of colorless oil. ^{1}H NMR (400 MHz, $CDCl_3$) δ 7.98 – 7.93 (m, 2H), 7.25 – 7.21 (m, 2H), 6.80 (d, J = 2.1 Hz, 1H), 6.67 (d, J = 2.1 Hz, 1H), 5.75 (s, 1H), 3.96 (s, 2H), 3.90 (s, 3H),

3.29 (s, 3H), 3.26 – 3.22 (m, 1H), 2.07 (t, J = 3.3 Hz, 1H), 1.96 – 1.87 (m, 5H), 1.84 – 1.75 (m, 4H), 1.71 (dd, J = 11.8, 2.8 Hz, 2H), 1.62 (s, 1H). ^{13}C NMR (101 MHz, $CDCl_3$) δ 167.14, 151.80, 145.85, 139.94, 139.80, 135.10, 132.04, 130.04, 128.94, 128.44, 124.54, 118.70, 116.06, 60.55, 57.25, 52.20, 41.44, 39.17, 37.21, 33.00, 29.83, 29.77, 28.47, 28.36, 25.78, 21.18, 14.33. MS (ESI+): m/z calc. for $C_{27}H_{29}ClO_4$: 452.5; found: 453.2 $[M+H]^+$.

1.3.18 Compound **15**

Methyl 4-(3-(((1r,3r,5R,7S)-adamantan-2-ylidene)(methoxy)methyl)-4-chloro-5-hydroxybenzyl) benzoate (**14**, 350 mg, 0.77 mmol) and potassium carbonate (118 mg, 0.85 mmol) were dissolved in dry DMF (10 mL). The solution was stirred at room temperature for 10 minutes and then 2-(4-(iodomethyl)phenyl)-4,4,5,5-tetramethyl-1,3,2-dioxaborolane (**9**, 345 mg, 1 mmol) was added into above mixture. The solution was stirred at room temperature and monitored by TLC. Upon completion, the mixture was diluted with EA and washed with 1M NH_4Cl as well as brine. The organic phase was dried over Na_2SO_4 and concentrated under reduced pressure. The crude product was purified by column chromatography (EA:Hex=1:4) to afford 489 mg (95 % yield) of white solid. ^{1}H NMR (400 MHz, $CDCl_3$) δ 7.98 – 7.92 (m, 2H), 7.85 – 7.79 (m, 2H), 7.46 – 7.42 (m, 2H), 7.21 – 7.16 (m, 2H), 6.73 – 6.68 (m, 2H), 5.10 (s, 2H), 3.96 (s, 2H), 3.91 (s, 3H), 3.30 (s, 3H), 3.27 – 3.23 (m, 1H), 1.96 – 1.88 (m, 5H), 1.85 – 1.74 (m, 4H), 1.70 (dt, J = 12.2, 2.8 Hz, 2H), 1.64 – 1.56 (m, 2H), 1.35 (s, 12H). ^{13}C NMR (101 MHz, $CDCl_3$) δ 167.11, 154.50, 145.90, 139.97, 139.58, 138.99, 136.22, 135.22, 135.15, 131.30, 130.16, 130.04, 129.94, 128.90, 128.46, 127.49, 126.51, 124.98, 122.15, 114.03, 83.97, 70.99, 65.67, 60.53, 57.17, 52.19, 41.65, 39.23, 39.17, 38.79, 38.69, 37.28, 33.01, 29.83, 29.74, 28.55, 28.39, 25.01, 25.01, 14.34. MS (ESI+): m/z calc. for $C_{40}H_{46}BClO_6$: 668.3; found: 691.4 $[M+Na]^+$.

1.3.19 Compound **16 (Diox)**

Methyl 4-(3-(((1r,3r,5R,7S)-adamantan-2-ylidene)(methoxy)methyl)-4-chloro-5-((4-(4,4,5,5-tetramethyl-1,3,2-dioxaborolan-2-yl)benzyl)oxy)benzyl)benzoate (**15**, 30 mg, 0.04 mmol) and a catalytic amount of methylene blue (0.7 mg) were dissolved in 5 mL dry DCM. Oxygen was bubbled through the mixture while irradiating with yellow light. The reaction was monitored by TLC. Upon completion, the solvent was concentrated under reduced pressure and the crude product was passed through a silica gel column (EA : Hex = 1:2) to remove methylene blue to afford 26 mg (95 % yield) of white solid. ^{1}H NMR (400 MHz, $CDCl_3$) δ 7.96 (d, J = 8.3 Hz, 2H), 7.79 (d, J = 8.0 Hz, 2H), 7.58 (d, J = 2.0 Hz, 1H), 7.39 (d, J = 7.7 Hz, 2H), 7.18 (d, J = 8.0 Hz, 2H), 6.78 (d, J = 2.1 Hz, 1H), 5.08 (s, 2H), 4.03 (s, 2H), 3.91 (s, 3H), 3.23 (s, 3H), 3.01 (s, 1H), 2.35 (d, J = 12.8 Hz, 1H), 2.01 – 1.98 (m, 1H), 1.89 – 1.78 (m, 2H), 1.76 – 1.56 (m, 8H), 1.46 (dq, J = 12.7, 2.7 Hz, 1H), 1.35 (s, 12H). ^{13}C NMR (101 MHz, $CDCl_3$) δ 171.29, 167.08, 154.98, 145.58, 139.43, 139.26, 135.16, 133.41, 130.14, 130.09, 129.01, 128.94, 128.58, 126.52, 126.46, 126.19, 119.98, 116.10, 112.26, 96.49, 84.01, 71.26, 60.53, 52.21, 49.85, 47.12, 41.78, 39.40, 36.79, 33.98, 33.72, 32.75, 32.48, 31.72, 31.58, 26.35, 25.95, 25.01, 21.19, 14.34. MS (ESI+): m/z calc. for $C_{40}H_{46}BClO_8$: 700.3; found: 723.3 $[M+Na]^+$.

1.3.20 Compound **17 (Me-Diox@Ru)**

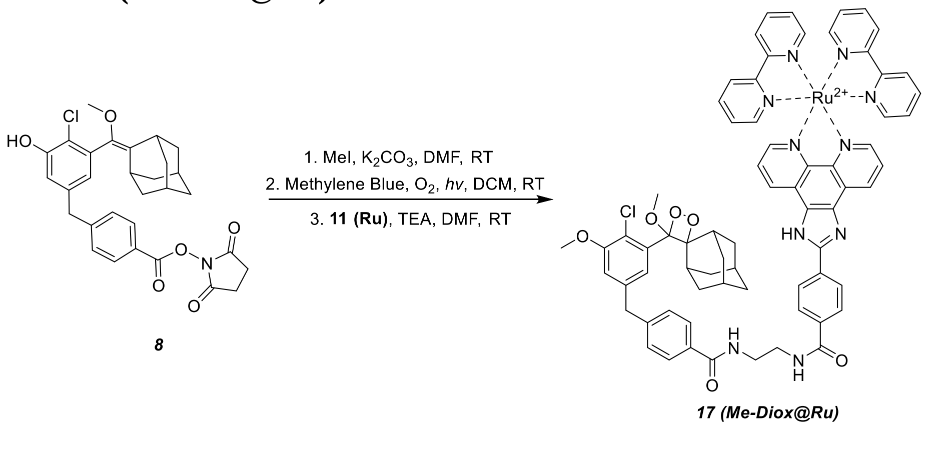

2,5-dioxopyrrolidin-1-yl 4-(3-(((1r,3r,5R,7S)-adamantan-2-ylidene)(methoxy)methyl)-4-chloro-5-hydroxybenzyl)benzoate (**8**, 300 mg, 0.56 mmol) and potassium carbonate (85 mg, 0.62 mmol) were dissolved in dry DMF (10 mL). The solution was stirred at room temperature for 10 minutes and then MeI (103.7 mg, 0.73 mmol) was added into the above mixture. The solution was stirred at room temperature and monitored by TLC. Upon completion, the mixture was diluted with EA and washed with 1M NH_4Cl as well as brine. The organic phase was dried over Na_2SO_4 and concentrated under reduced pressure. The crude product and a catalytic amount of methylene blue were dissolved in 5 mL dry DCM. Oxygen was bubbled through the mixture while irradiating with yellow light. The reaction was monitored by TLC. Upon completion, the solvent was concentrated under reduced pressure and the crude product was passed through a silica gel column (EA : Hex = 1:2) to remove methylene blue. The solution was concentrated and dried to obtain a white solid. Lastly, the solid (11 mg) and Ru (**11**, 11.2 mg) were dissolved in 3 mL dry DMF. Then trimethylamine (0.02 mL) were added to the above mixture. The reaction was stirred at room temperature and monitored by LCMS. Upon completion, the solution precipitates as a solid when added dropwise to cold diethyl ether. The product was dried to afford 11.5 mg (93 % yield) of red solid. ^{1}H NMR (400 MHz, MeOD-d4) δ 9.14 (dd, J = 8.3, 1.3 Hz, 2H), 8.74 (ddt, J = 16.0, 8.2, 1.1 Hz, 4H), 8.37 (d, J = 8.4 Hz, 2H), 8.18 (td, J = 7.9, 1.5 Hz, 2H), 8.14 – 8.00 (m, 6H), 7.95 (ddd, J = 5.7, 1.5, 0.7 Hz, 2H), 7.87 (dd, J = 8.3, 5.3 Hz, 2H), 7.81 – 7.76 (m, 2H), 7.70 (dt, J = 5.6, 1.2 Hz, 2H), 7.56 (ddd, J = 7.7, 5.6, 1.3 Hz, 2H), 7.46 (s, 1H), 7.33 (ddd, J = 7.4, 5.8, 1.3 Hz, 4H), 7.07 (d, J = 2.0 Hz, 1H), 4.10 (s, 2H), 3.89 – 3.81 (m, 3H), 3.66 (s, 4H), 3.10 (s, 3H), 2.84 (s, 1H), 2.40 – 2.26 (m, 1H), 2.00 – 1.46 (m, 9H), 1.44 – 1.21 (m, 3H). ^{13}C NMR (101 MHz, MeOD-d4) δ 170.61, 169.69, 158.79, 158.60, 152.84, 152.74, 151.17, 147.09, 146.12, 141.82, 139.31, 139.17, 137.17, 134.21, 133.69, 131.98, 130.02, 129.28, 128.94, 128.85, 128.69, 127.95, 127.29, 125.65,

125.58, 115.40, 57.00, 42.29, 41.09, 40.73, 37.59, 34.79, 33.20, 32.75, 27.62, 27.27. LC retention time: 6.6 min. MS (ESI+): m/z calc. for $C_{69}H_{61}ClN_{10}O_6Ru^{2+}$: 631.2; found: 631.5 $[M-2H^+-2Cl^-]^{2+}$.

1.4 Chemiluminescence Studies

Chemiluminescence kinetic profiles and emission spectra were tested in white 96-well plates with or without H_2O_2 by Tecan Spark 20M microplate reader. All samples were prepared in DPBS (1x, pH=7.4) and 100 µL solution per well was used to test. More details for every tested sample can be found in the subtitles of the figures.

1.5 $^1O_2$ Detection - ABDA

The $^1O_2$ generation was assessed by employing ABDA as an indicator. The absorption spectra of ABDA were monitored in a range of 320 - 800 nm without irradiation and the absorbance decrease of ABDA at 380 nm was recorded to indicate the decomposition rates of ABDA ($^1O_2$ generation rate). All samples were prepared in DPBS and 100 µL solution per well was used to test in a transparent 384-well plate. More details for every tested sample can be found in the figure captions.

1.6 pH-Stability Studies

The pH stability of Diox@Ru in Tris-HCl solution from pH 3-10 was measured by recording their total chemiluminescence intensity in 2h without adding H_2O_2 on Tecan Spark 20M microplate reader. Tris-HCl solution (50 mM) was prepared by dissolving Tris base in DPBS and then adjusting the pH to the desired value by adding concentrated HCl solution. The pH stability of Diox@Ru (250 µM) in Tris-HCl solution was tested with 100 µL solution per well in half white 96-well plate.

1.7 Light-Induced Decomposition Studies

The stability of Diox@Ru and Me-Diox@Ru under physiological conditions was assessed by HPLC-MS both in darkness and ambient light. Diox@Ru (400 μM) and Me-Diox@Ru (400 μM) were incubated in DPBS (1x, pH = 7.4) at ambient temperature for 48h. Reaction solutions of identical concentration and volume were subjected to LCMS analysis, quantifying the decomposition of both Diox@Ru and Me-Diox@Ru based on the relative peak area integration compared to their initial values at 254 nm.

1.8 Tumor Biomarker Specificity

The tumor biomarkers responsive studies were measured by recording their total chemiluminescence intensity in 2h on Tecan Spark 20M microplate reader. The Diox@Ru (50 μM) was incubated in DPBS (1x, pH=7.4) with hydrogen peroxide (H_2O_2, 50 μM), L-glutathione (GSH, 10 mM), lactate dehydrogenase from porcine heart (LDH, 0.5 units/mL) and matrix metalloproteinase-1 (MMP-1, 500 ng/mL).

1.9 Cell Culture

In vitro studies were conducted using A549 lung carcinoma cells (DSMZ), 4T1 mouse mammary carcinoma cell (ATCC, #CRL-2539) or HaCaT human keratinocyte cells (provided by Michael Delacher, Institute of Immunology, University Medical Center Mainz). 4T1 and A549 cells were cultured at 37 °C and 5% CO_2 in Dulbecco's Modified Eagle's Medium (DMEM, high glucose), supplemented with 10% FBS. HaCaT cells were cultured for 5 days at 37 °C and 5% CO_2 in DMEM (high glucose, NEAA, Gibco), supplemented with 10% FCS (Gibco) before being used for further analysis. 4T1, A549 and HaCaT cell lines were cultured in T75 culture flask and

subcultivated two to three times per week.

For cellular assays, DMSO concentrations did not exceed 2% (v/v) as a co-solvent. The nature of polypyridyl ligands and the positive charge associated with the ruthenium(II) center within Diox@Ru enhances its aqueous solubility[5,6] and fetal bovine serum present in the culture medium facilitates its cellular uptake[7,8].

1.10 Cellular Internalization Assay

A549 and 4T1 cells were seeded for overnight adherence at 25000 cells/well in a transparent 8-well plate. Then the medium was removed by aspiration and 200 µL diluted samples (50 µM) were added into each well. Cells were incubated at 37 °C and 5% CO_2 for 8h. The medium was removed by aspiration and cells were fixed with 4% PFA by the protocol. Images were obtained by Confocal Laser Scanning Microscopy.

1.11 Intracellular Colocalization Assay

4T1 cells were seeded for overnight adherence at 25000 cells/well in a transparent 8-well plate. Then the medium was removed by aspiration and 200 µL diluted samples (50 µM) were added into each well. Cells were incubated at 37 °C and 5% CO_2 for 8h. For nucleus colocalization, the medium was removed and cells were stained with NucBlue™ Live ReadyProbes™ Reagent (Hoechst 33342) by the protocol. For mitochondria colocalization, the medium was removed and cells were stained with 200 µL MitoTracker Orange CMTMRos (0.5 µM) by the protocol. Images were obtained by Confocal Laser Scanning Microscopy. The Pearson's R was calculated by ImageJ with colocalization analysis.

1.12 Intracellular Chemiluminescence Assay

A549 and 4T1 cells were seeded for overnight adherence at 10000 cells/well in white 96-well plates. The medium was removed by aspiration and 100 µL diluted samples (50 µM) were added into each well. Cells were incubated at 37 °C and 5% CO_2 for 4h. The medium was removed by aspiration and 200 µL DPBS was added and removed into each well. Lastly, 100 µL DPBS was added into each well and chemiluminescence was tested by Tecan Spark 20M microplate reader.

1.13 Cell Viability - CellTiterGlo Luminescent Cell Viability Assay

A549 and 4T1 cells were seeded for overnight adherence at 10000 cells/well in white 96-well plates. The medium was removed by aspiration and 100 µL of the samples (0-40 µM, more details for every tested sample could be found in the figure captions) were added into each well. Cells were incubated at 37 °C and 5% CO_2 for 24h. The medium was removed by aspiration and 200 µL DPBS was added and removed into each well. 100 µL CellTiterGlo Luminescent Cell Viability Assay reagent was added into each well and the plate was covered with foil and rested in the cell bench for 10 minutes. Luminescence data were recorded on Tecan Spark 20M microplate reader.

1.14 Live/Dead Cell Viability Assay - Calcein-AM/PI

A549 and 4T1 cells were seeded for overnight adherence at 25000 cells/well in a transparent 8-well plate. The medium was removed by aspiration and 200 µL of the samples (50 µM) were added into each well. Cells were incubated at 37 °C and 5% CO_2 for 8h. The medium was removed by aspiration and 200 µL DPBS was added and removed into each well. Then 200 µL Calcein-AM (2 µM)/PI (4.5 µM) reagent was added into each well and cells were incubated at 37 °C and 5% CO_2 for 20 minutes. Images were obtained by Keyence Microscope BZX800.

1.15 Intracellular ROS Assay - DCFH-DA

A549 and 4T1 cells were seeded for overnight adherence at 25000 cells/well in a transparent 8-well plate. The medium was removed by aspiration and 200 μL of the samples (50 μM) were added into each well. Cells were incubated at 37 °C and 5% CO_2 for 8h. The medium was removed by aspiration and 200 μL DPBS was added and removed into each well. Then 200 μL DCFH-DA (10 μM) reagent was added into each well and cells were incubated at 37 °C and 5% CO_2 for 20 minutes. The medium was removed and each well was washed 3 times with 200 μL DPBS. Lastly, 200 μL DPBS was added into each well and images were obtained by Keyence Microscope BZX800.

1.16 Cell Apoptosis Assay - Annexin V-FITC

A549 and 4T1 cells were seeded for overnight adherence at 25000 cells/well in a transparent 8-well plate. The medium was removed by aspiration and 200 μL diluted samples (50 μM) were added into each well. Cells were incubated at 37 °C and 5% CO_2 for 8h. The medium was removed by aspiration and 200 μL Annexin V binding buffer was added and removed into each well. Then 200 μL Annexin V-FITC reagent was added into each well and the plate was covered with foil and rested in the cell bench for 15 minutes. The medium was removed by aspiration and 200 μL Annexin V binding buffer was added and removed into each well. Lastly, 200 μL Annexin V binding buffer was added into each well and images were obtained by Keyence Microscope BZX800.

1.17 Tumor Spheroids

4T1 cells were seeded for 7 days at 10000 cells/well in 96-well plates and the medium was removed. Then 150 μL of samples (50 μM) were added into each well and tumor spheroids were incubated at 37 °C and 5% CO_2 for 24 h. For Internalization, the medium was removed and tumor spheroids were fixed with 4% PFA and stained with Hoechst 33342 by the protocol. For ROS, the medium was removed and tumor spheroids were stained with DCFH-DA (10 μM) and then stained with Hoechst 33342 by the protocol. Images were obtained by Confocal Laser Scanning Microscopy. For cell viability, 100 µL CellTiter-Glo® 3D Cell Viability Assay reagent was added into each well by the protocol and the plate was covered with foil and rested in the cell bench for 30 minutes. Luminescence data were recorded on Tecan Spark 20M microplate reader.

1.18 Cell Viability of HaCaT Cells

HaCaT cells were seeded for overnight adherence at 25000 cells/well in white 96-well plates. The medium was removed by aspiration and 100 μL of the samples with varying concentrations (0-100 µM) were added into each well. Cells were incubated at 37 °C and 5% CO_2 for 24h. The medium was removed by aspiration and 200 μL DPBS was added and removed into each well. 100 µL CellTiterGlo Luminescent Cell Viability Assay reagent was added into each well and the plate was covered with foil and rested in the cell bench for 10 minutes. Luminescence data were recorded on Tecan Spark 20M microplate reader.

1.19 Statistical Analysis of Cellular Experiments

To ensure statistically valid inference and enhance the reproducibility of cellular assays, we have conducted a series of independent experiments or technical replicates on internalization efficiency,

intracellular ROS generation, intracellular chemiluminescence and cell viability of Diox@Ru in both two-dimensional (2D) monolayer cultures and three-dimensional (3D) tumor spheroid models with 4T1 cells. For technical replicates, data are presented as data + SEM; independent experiments are indicated in figure captions. Methodological details are provided within the corresponding figure and its caption.

2. Supporting Figures

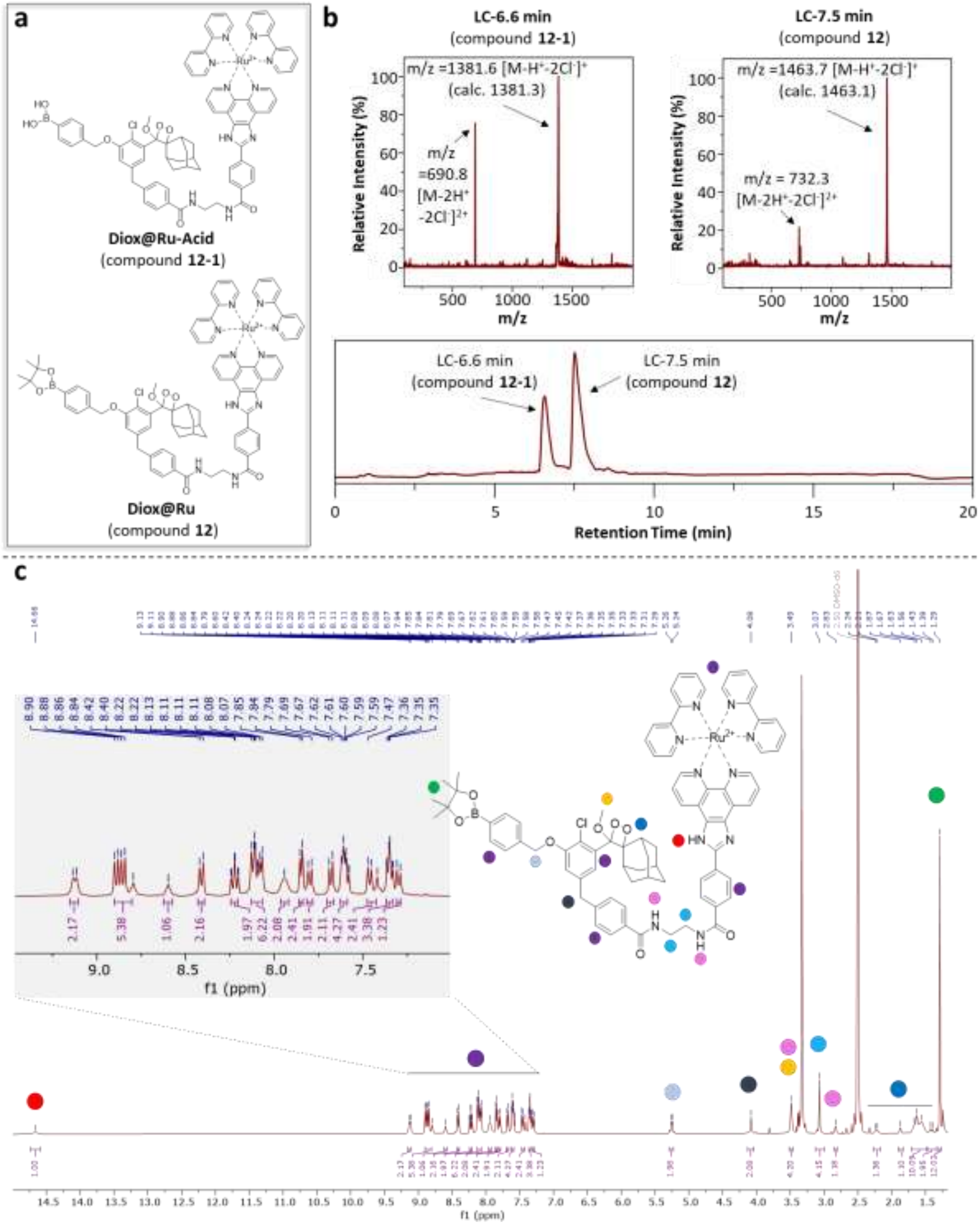

Figure S1. (a) Abbreviations of compounds. (b) LC-MS and (c) ^{1}H-NMR of Diox@Ru. ^{1}H spectrum in full range is given on page S54.

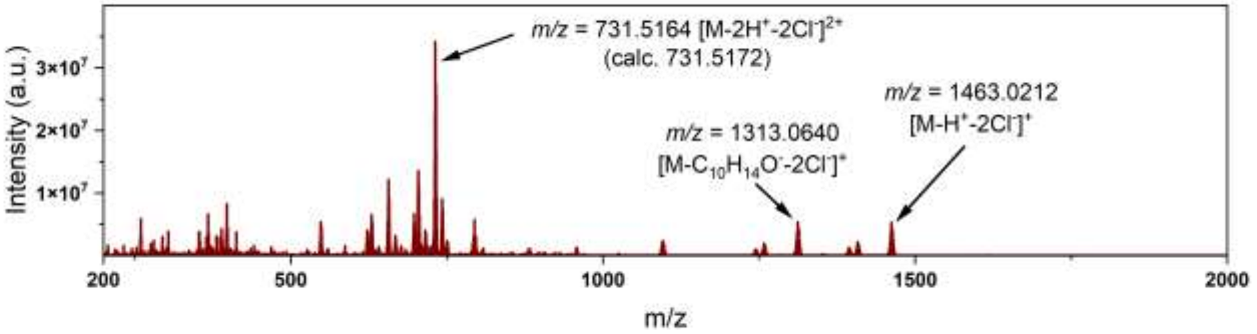

Figure S2. High Resolution-ESI (HR-ESI) spectrometry of Diox@Ru.

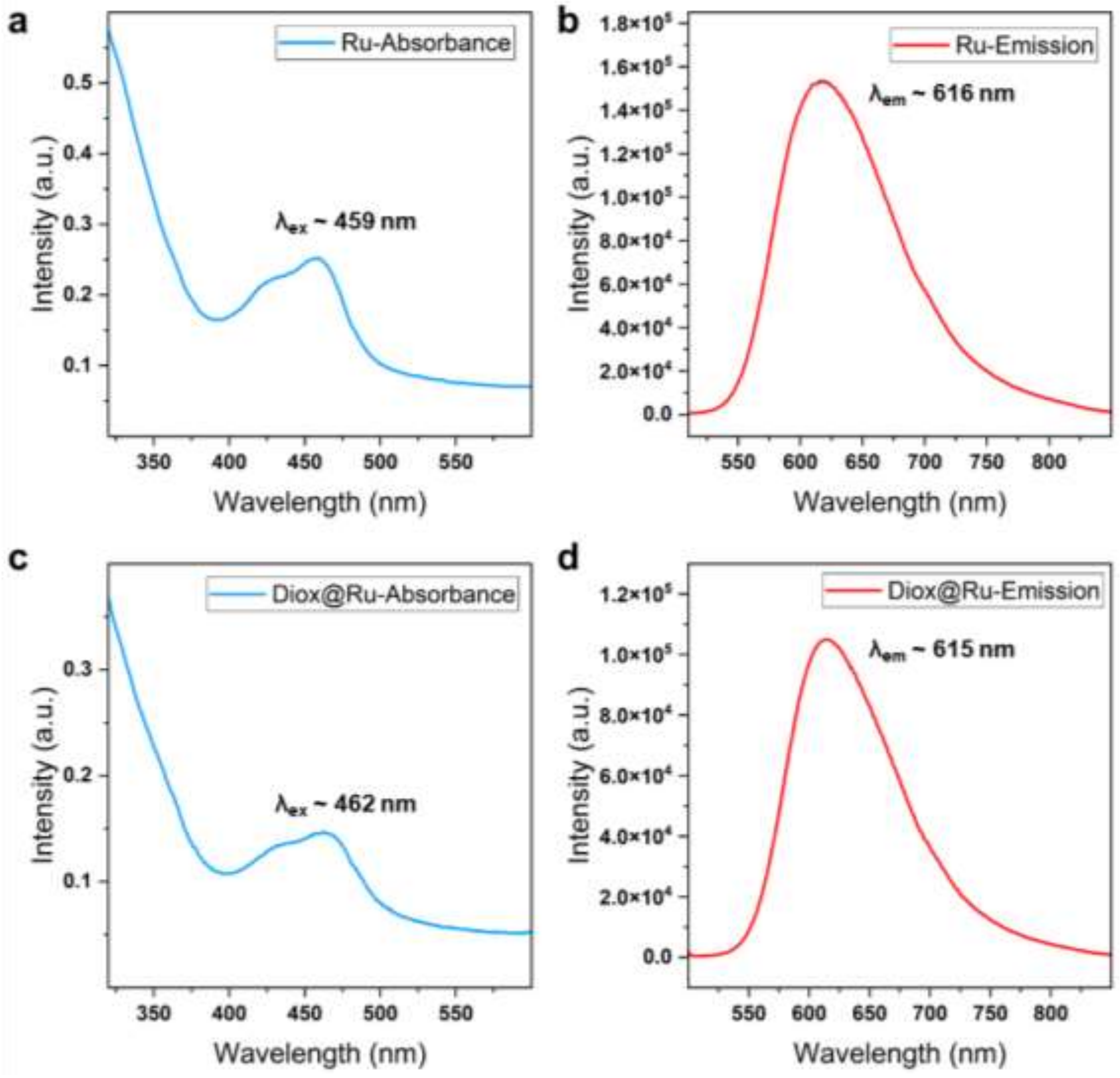

Figure S3. (a) Absorbance and (b) emission (λex = 459 nm) spectra of Ru (12.5 µM in DPBS, pH = 7.4). (c) Absorbance and (d) emission (λex = 462 nm) spectra of Diox@Ru (12.5 µM in DPBS, pH = 7.4).

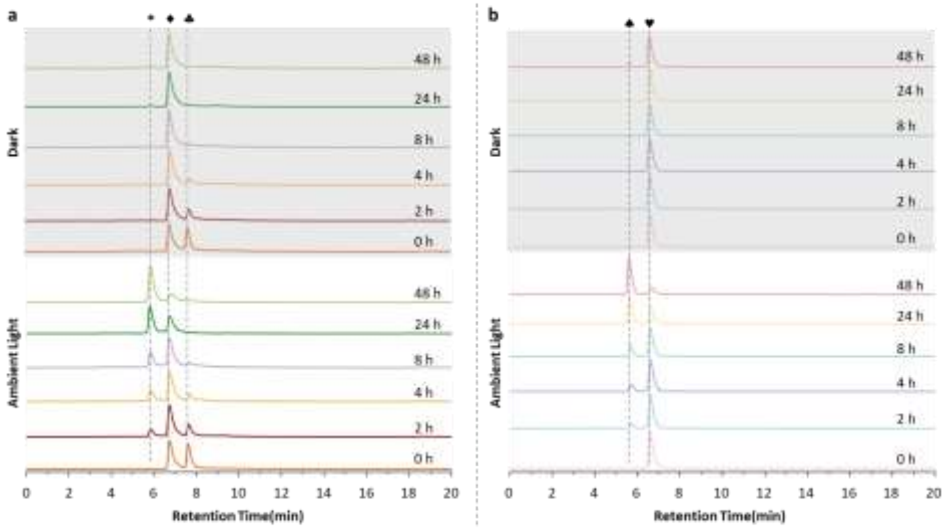

Figure S4. Full LC spectra of (a) Diox@Ru and (b) Me-Diox@Ru at ambient temperature in darkness and ambient light (400 μM in DPBS, pH = 7.4).

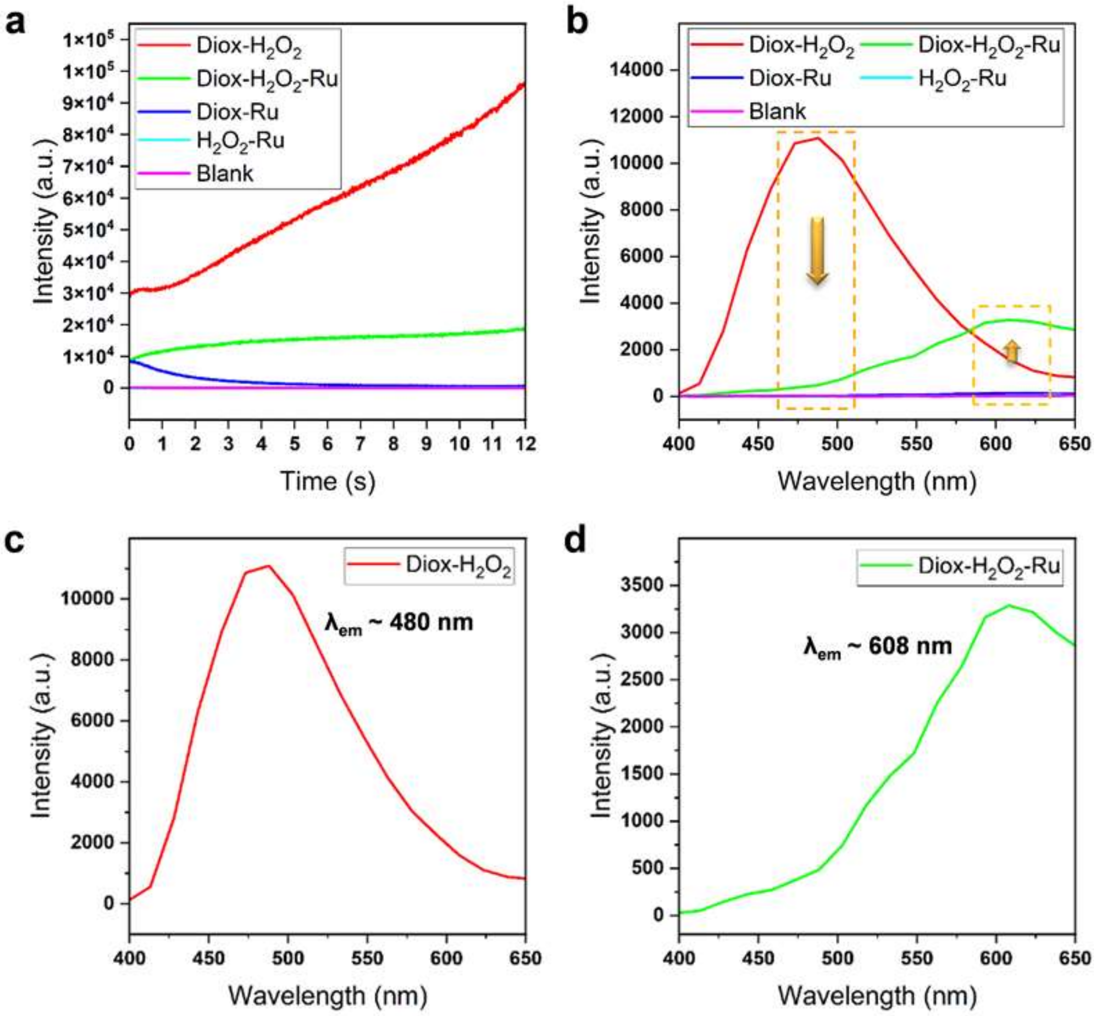

Figure S5. Comparison of chemiluminescence (a) kinetic profiles (360-700 nm) and (b-d) emission spectra (after 12 h) (250 μM Diox, 250 μM H_2O_2, 250 μM Ru, 25% DMSO in DPBS (1x, pH=7.4)).

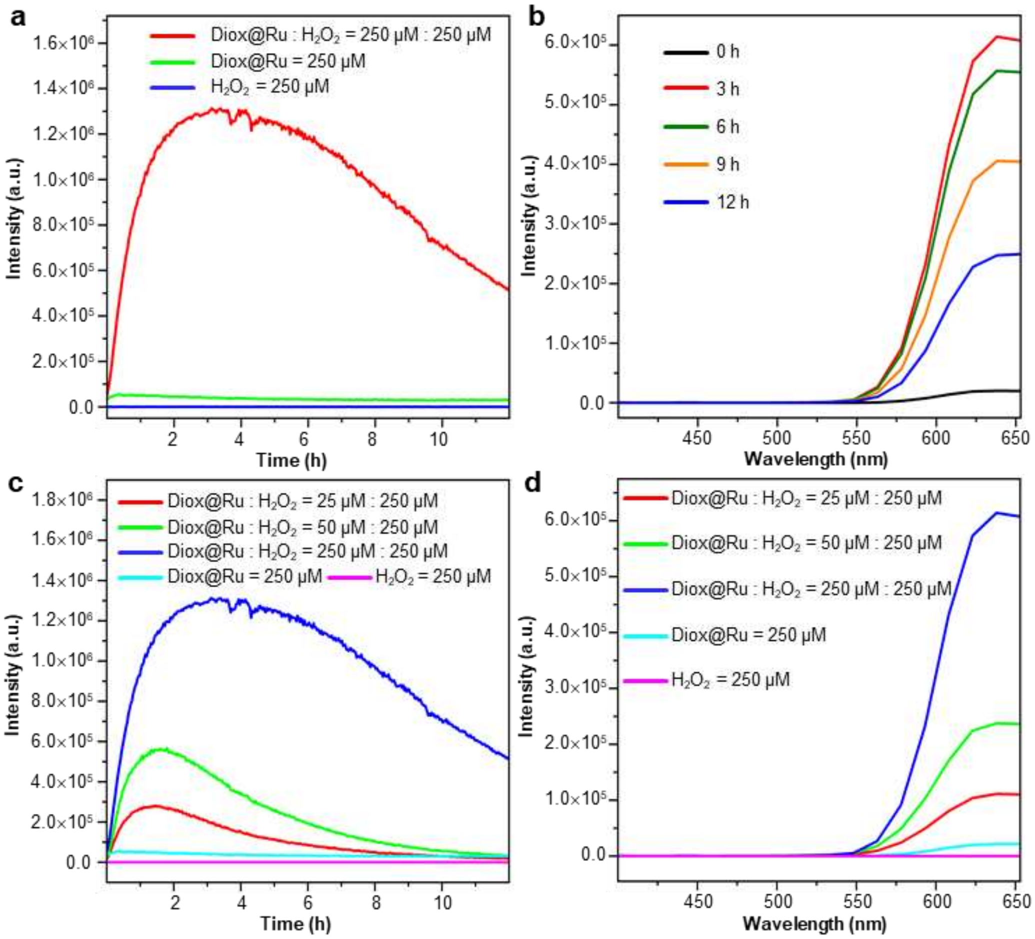

Figure S6. Photophysical properties of Diox@Ru. Chemiluminescence (a) kinetic profiles (360-700 nm) and (b) emission spectra (0-12h) of Diox@Ru (250 μM Diox@Ru, 250 μM H_2O_2, 25% DMSO in DPBS (1x, pH=7.4)). Various Diox@Ru and H_2O_2 concentration ratios of chemiluminescence (c) kinetic profiles (360-700 nm) and (d) emission spectra (2h).

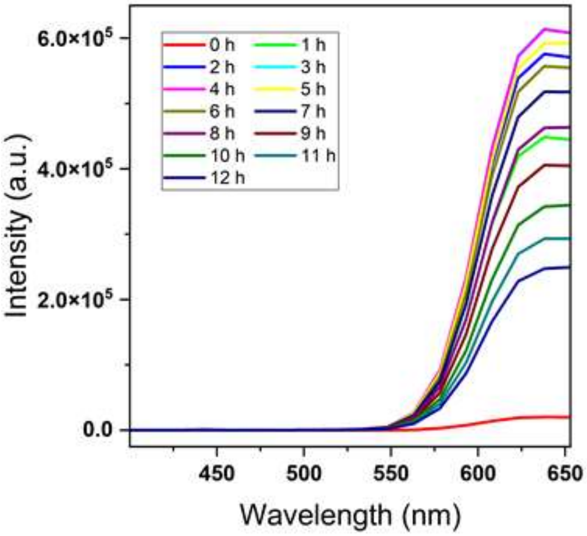

Figure S7. Chemiluminescence emission spectra (0-12h) of Diox@Ru (250 μM Diox@Ru, 250 μM H_2O_2, 25% DMSO in DPBS (1x, pH=7.4)).

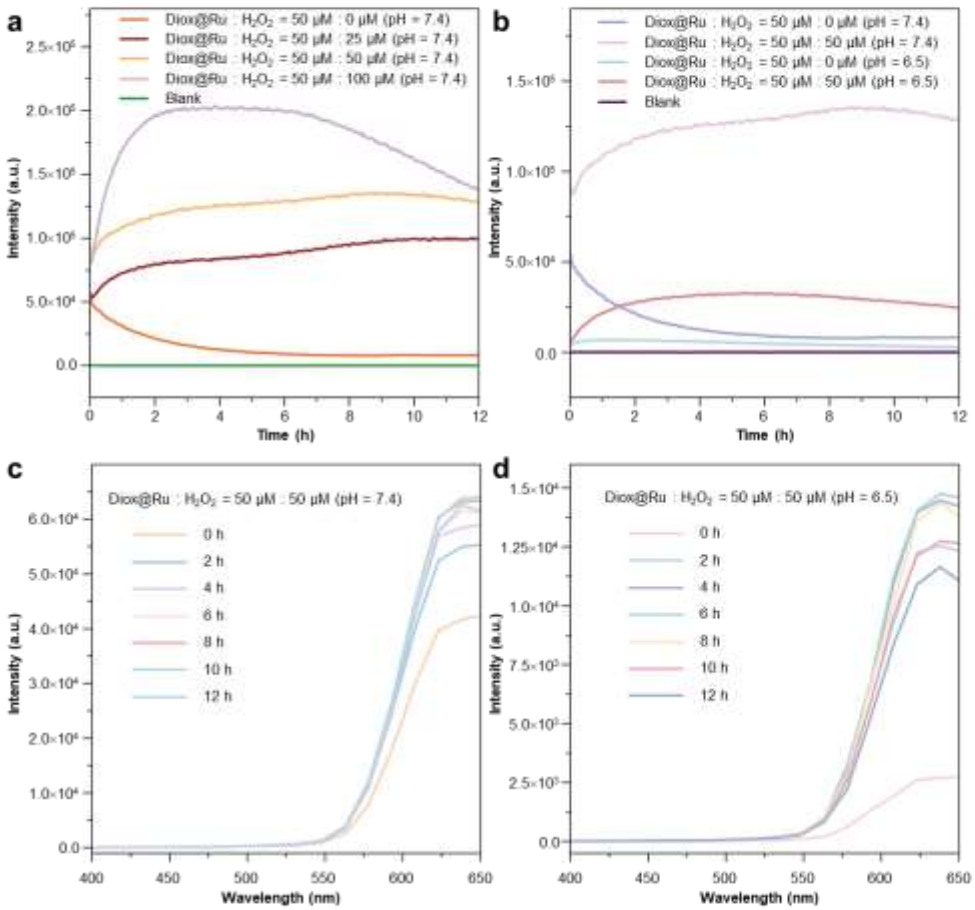

Figure S8. (a) Various Diox@Ru and H_2O_2 concentration ratios of chemiluminescence kinetic profiles (360-700 nm, 50 µM Diox@Ru in DPBS (1x, pH=7.4), 12h). (b) Comparisons of chemiluminescence kinetic profiles of Diox@Ru with low/high ROS and pH (360-700 nm, 50 µM Diox@Ru in DPBS), 12h). Chemiluminescence emission spectra (0-12h) of Diox@Ru at (c) pH = 7.4 and (d) pH = 6.5 (50 µM Diox@Ru, 50 µM H_2O_2 in DPBS).

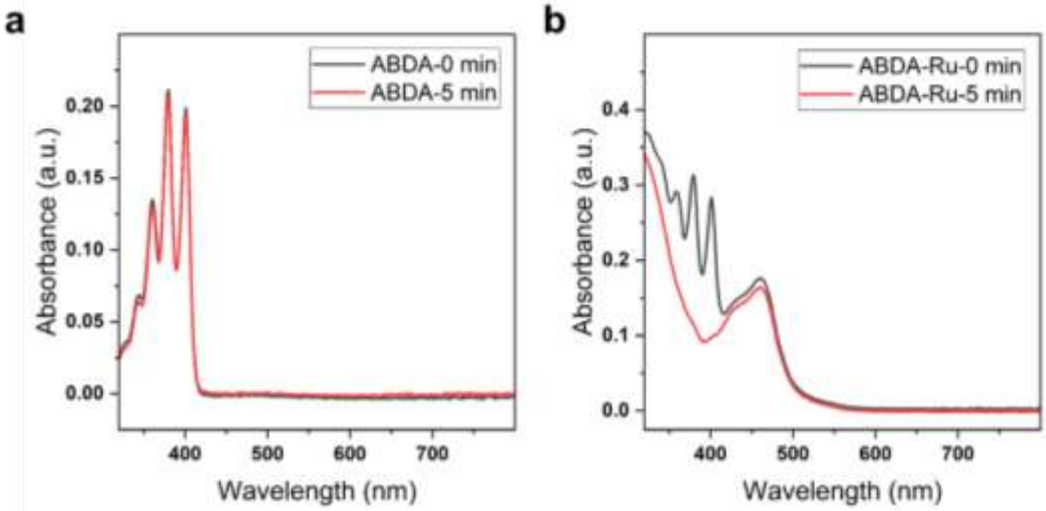

Figure S9. Absorbance curve of (a) ABDA (50 µM), and (b) Ru-ABDA (50 µM) before and after photo-irradiation using 470 nm LED lamp (~20 mW cm^{-2}) for 5 min.

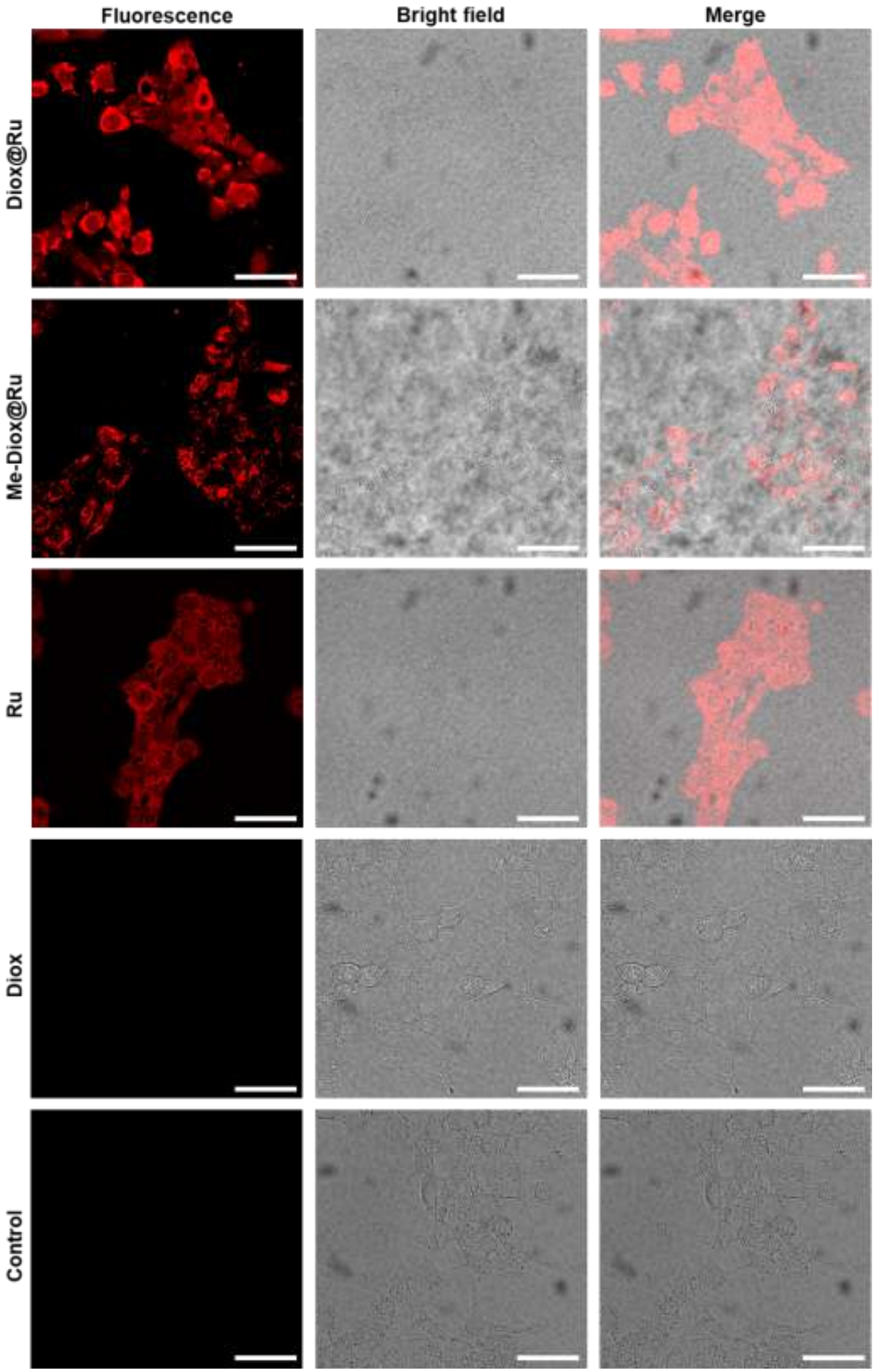

Figure S10. Internalization of Diox@Ru, Me-Diox@Ru, Ru and Diox into 4T1 cells (50 µM, 1% DMSO, incubating 8 h). Scale bar: 50 µm.

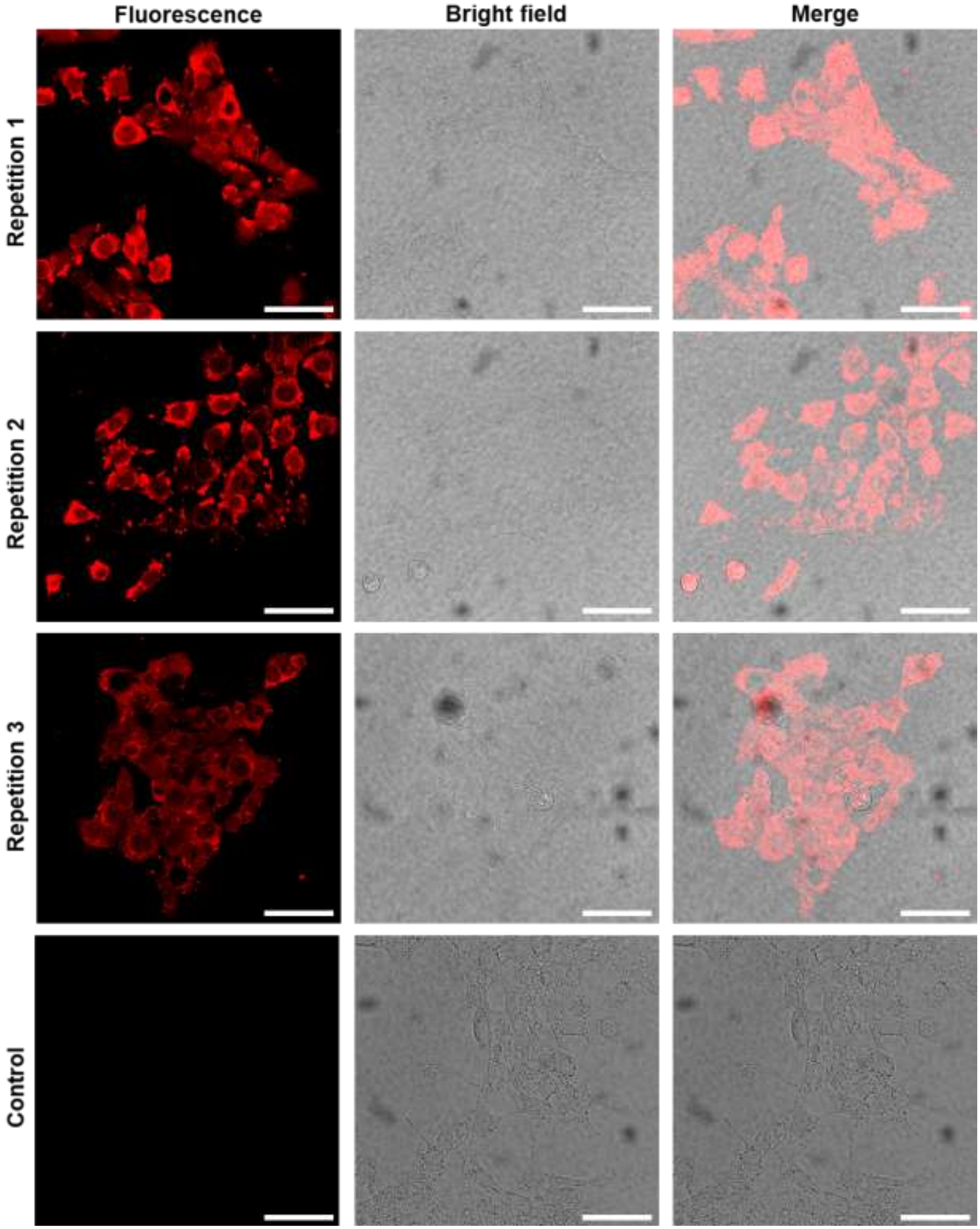

Figure S11. Independent experiments (replicates) performed for Diox@Ru into 4T1 cells (50 μM, 1% DMSO, incubating 8 h). Scale bar: 50 μm.

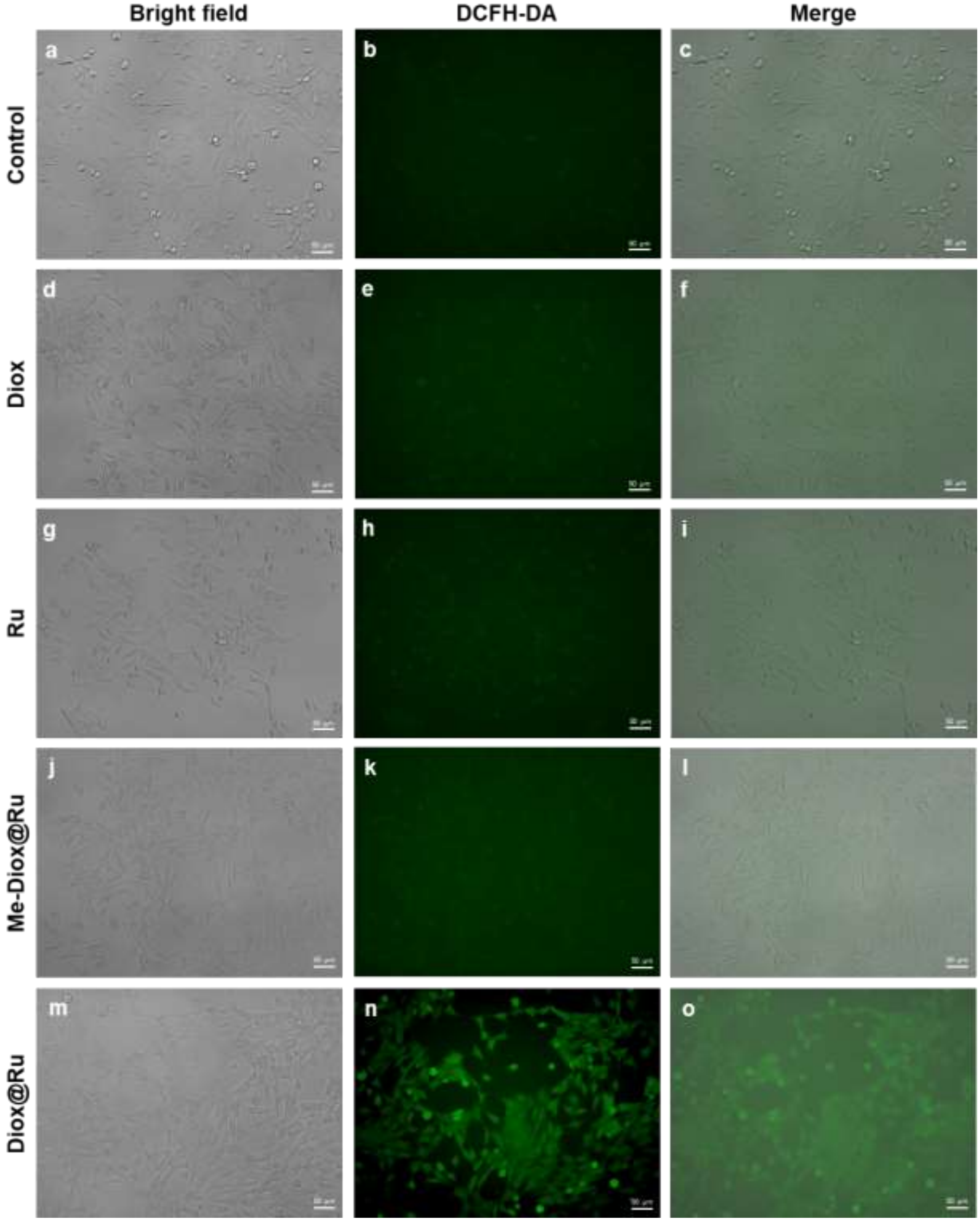

Figure S12. Intracellular ROS generation of (a-c) Control, (d-f) Diox, (g-i) Ru, (j-l) Me-Diox@Ru and (m-o) Diox@Ru with 4T1 cells (50 μM, 1% DMSO, incubating 8 h, DCFH-DA as an indicator). Scale bar: 50 μm.

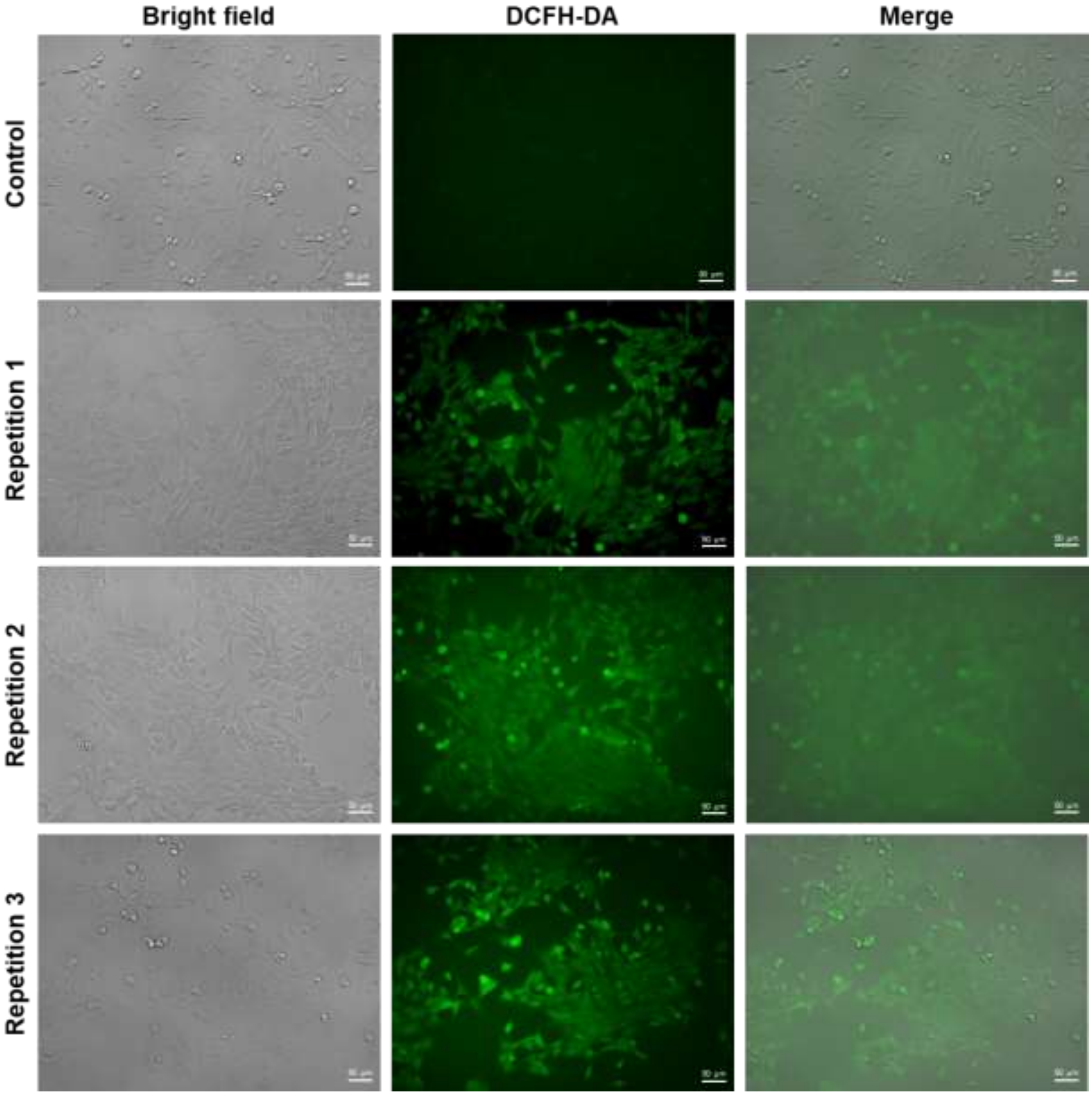

Figure S13. Independent experiments (replicates) performed for intracellular ROS generation of Diox@Ru with 4T1 cells (50 μM, 1% DMSO, incubating 8 h, DCFH-DA as an indicator). Scale bar: 50 μm.

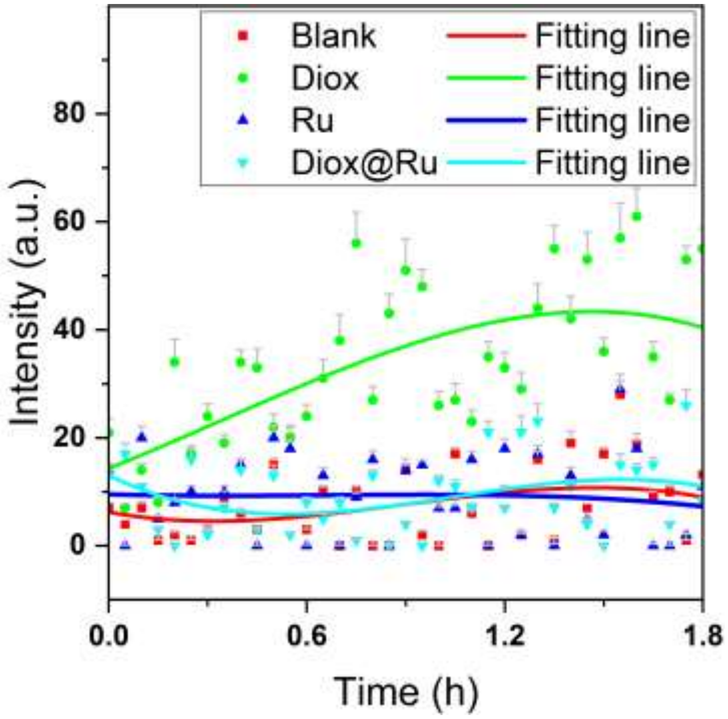

Figure S14. Intracellular chemiluminescence kinetic profiles of Diox@Ru in 4T1 cells (50 μM, 1% DMSO, incubating 4 h, recording wavelength: 460-500 nm. Data presented as S.E.M, n = 3, technical replicates).

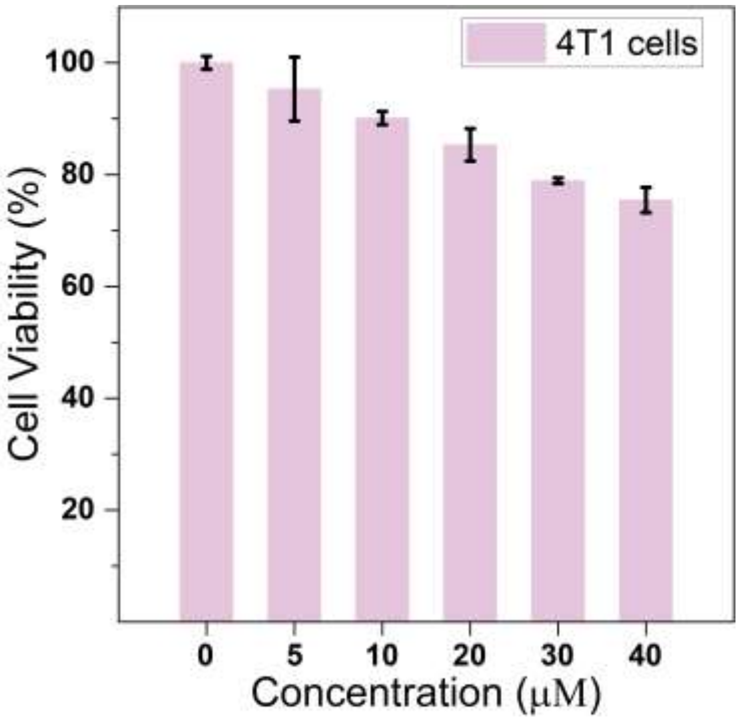

Figure S15. Cell viability of 4T1 cells with mixture of Diox and Ru (Diox-Ru) (< 1% DMSO, incubating 24 h, CellTiterGlo luminescent cell viability assay as an indicator). Data presented as S.E.M, n = 5.

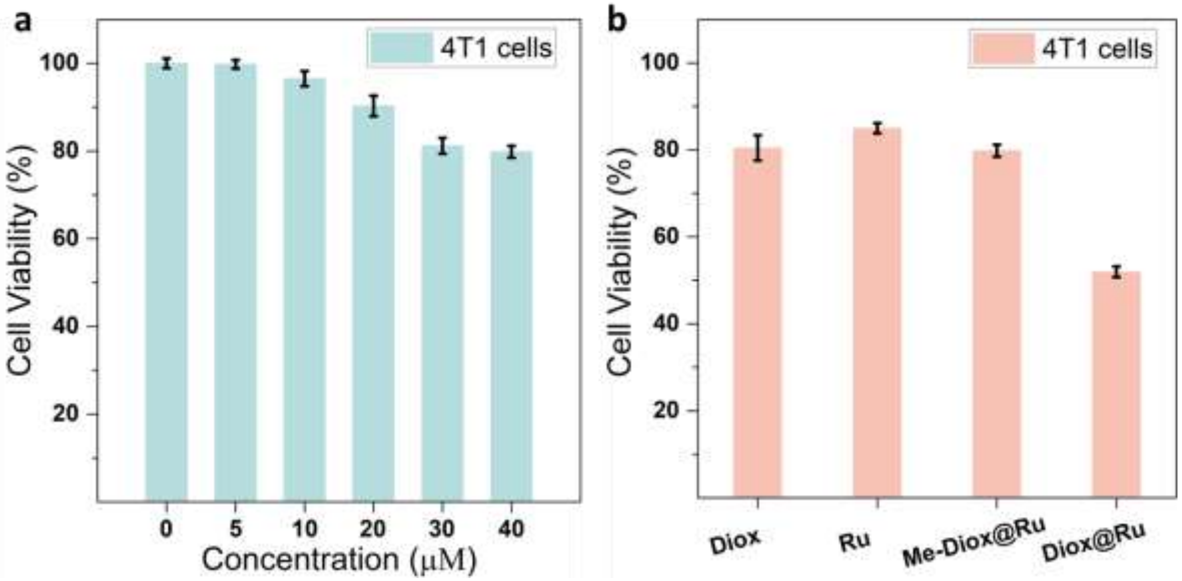

Figure S16. Cell viability of (a) Me-Diox@Ru (0 - 40 μM) and (b) comparisons of Me-Diox@Ru with Diox, Ru and Diox@Ru at identical concentrations (40 μM) in 4T1 cells (< 1% DMSO, incubating 24 h, CellTiterGlo luminescent cell viability assay as an indicator). Data presented as S.E.M, n = 5.

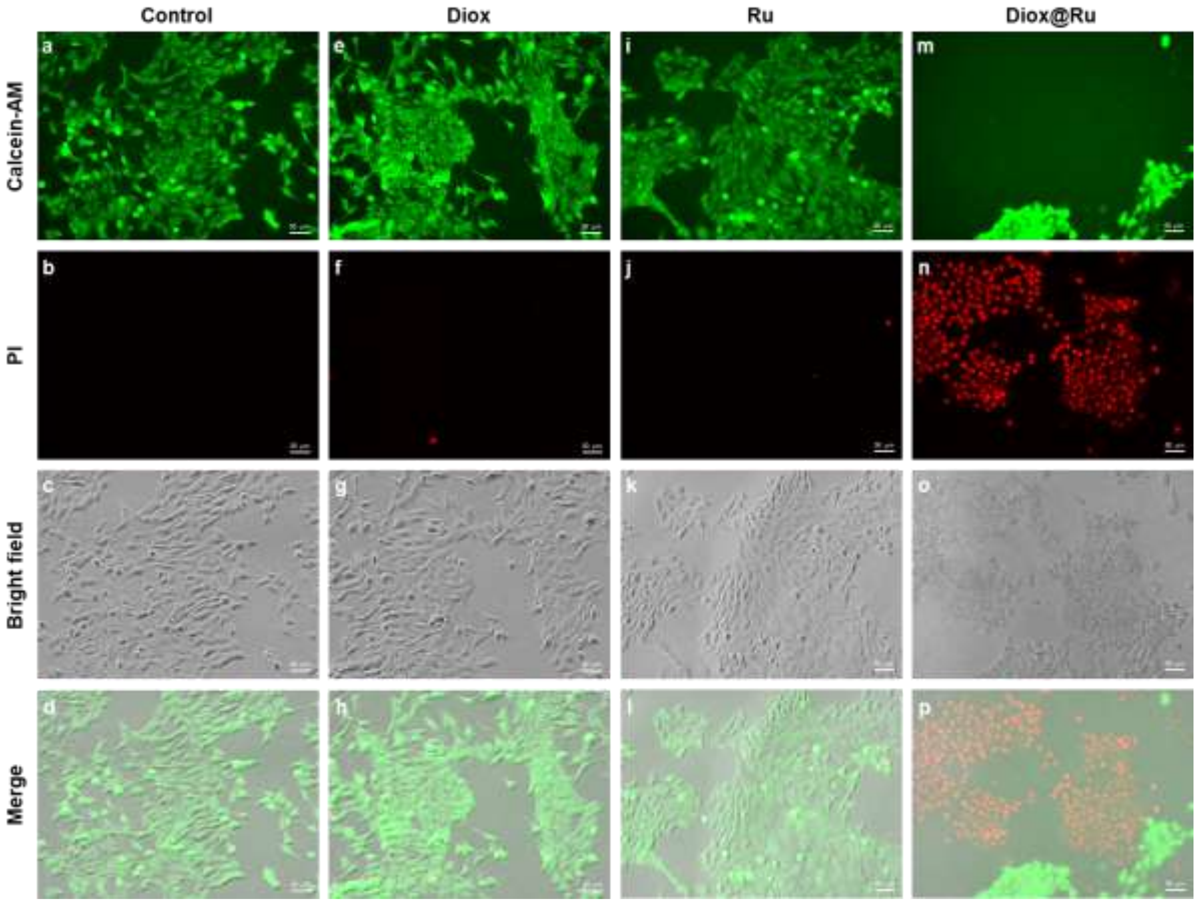

Figure S17. Live/Dead cell viability of (a-d) Control, (e-h) Diox, (i-l) Ru and (m-p) Diox@Ru with 4T1 cells (50 μM, 1% DMSO, incubating 8 h, Calcein-AM/PI as an indicator). Scale bar: 50 μm.

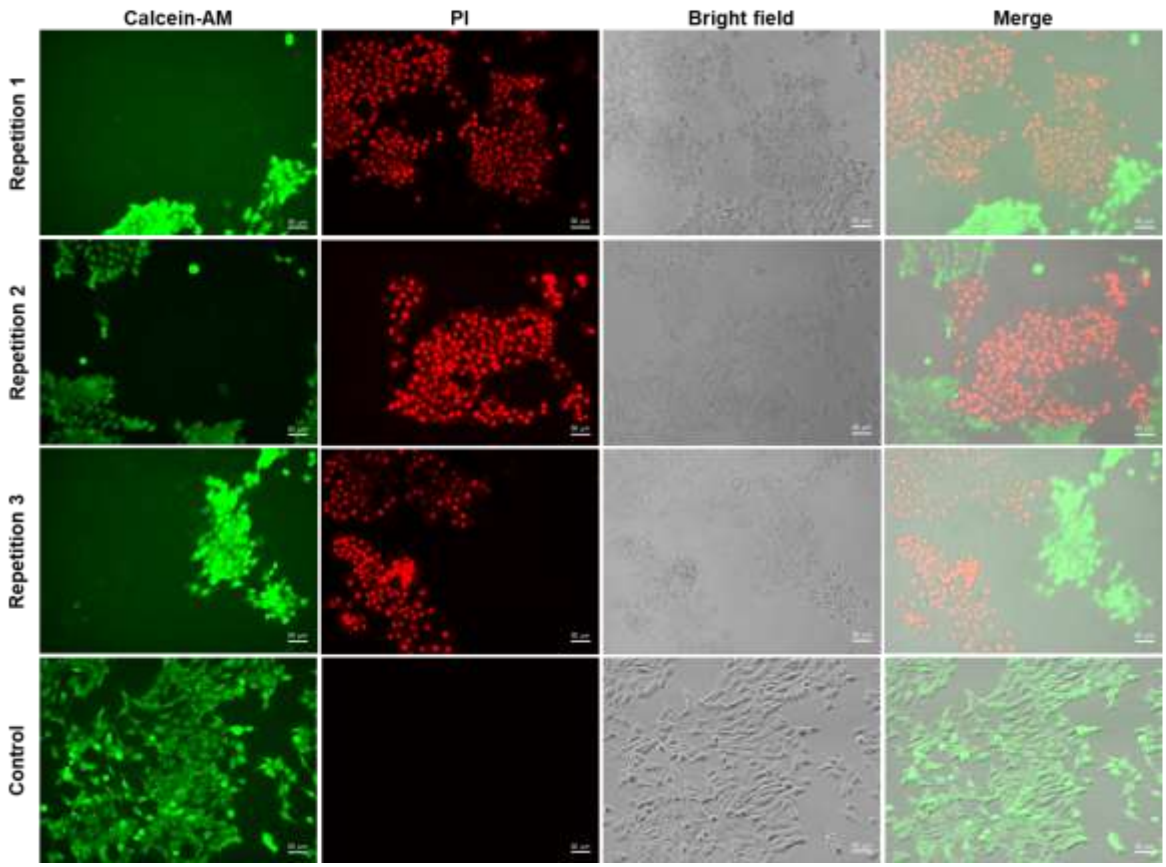

Figure S18. Independent experiments (replicates) performed for live/dead cell viability of Diox@Ru with 4T1 cells (50 μM, 1% DMSO, incubating 8 h, Calcein-AM/PI as an indicator). Scale bar: 50 μm.

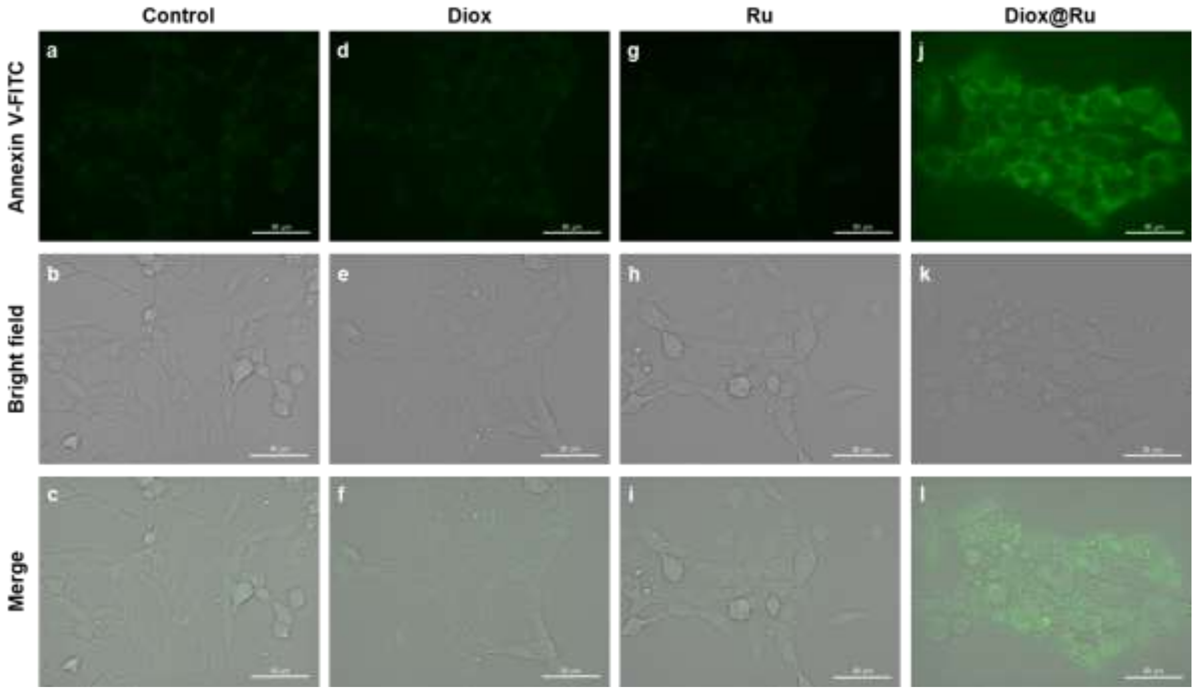

Figure S19. Cell apoptosis of (a-c) Control, (d-f) Diox, (g-i) Ru and (j-l) Diox@Ru with 4T1 cells (50 μM, 1% DMSO, incubating 8 h, Annexin V-FITC as an indicator). Scale bar: 50 μm.

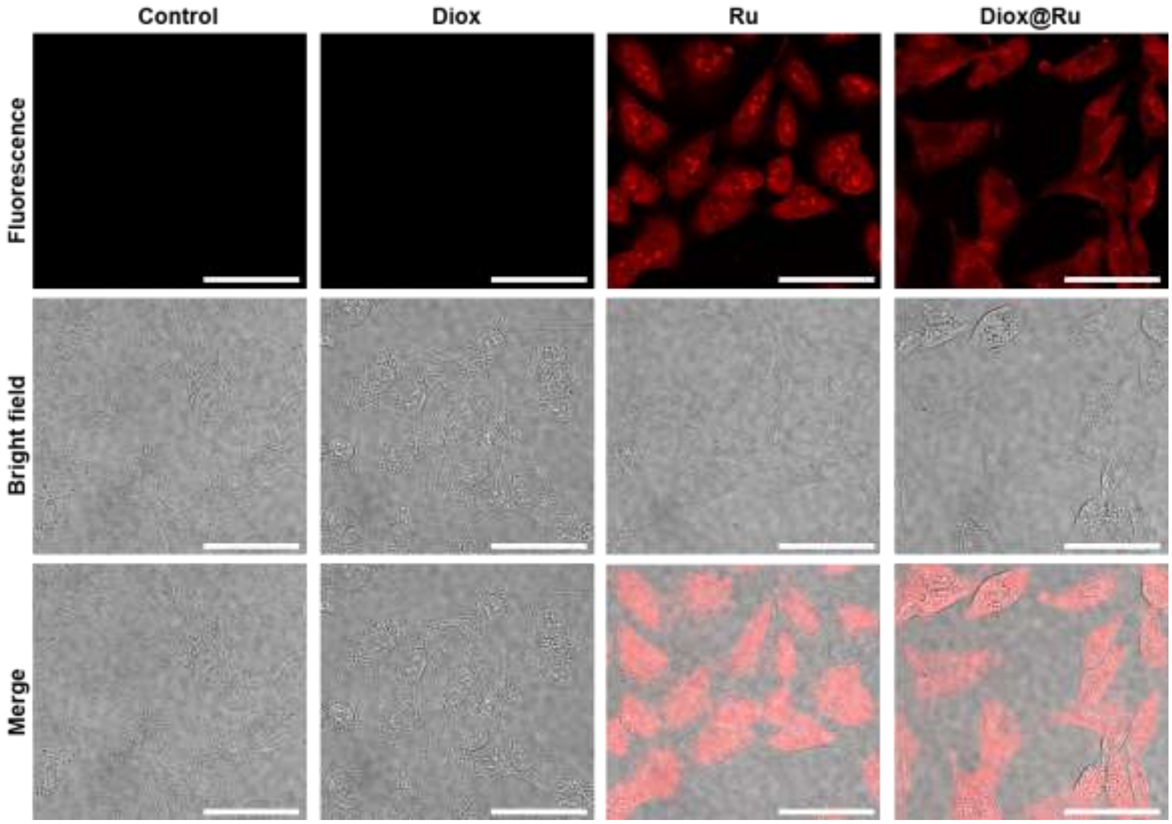

Figure S20. Internalization of Diox@Ru, Ru and Diox into A549 cells (50 μM, 1% DMSO, incubating 8h). Scale bar: 50 μm.

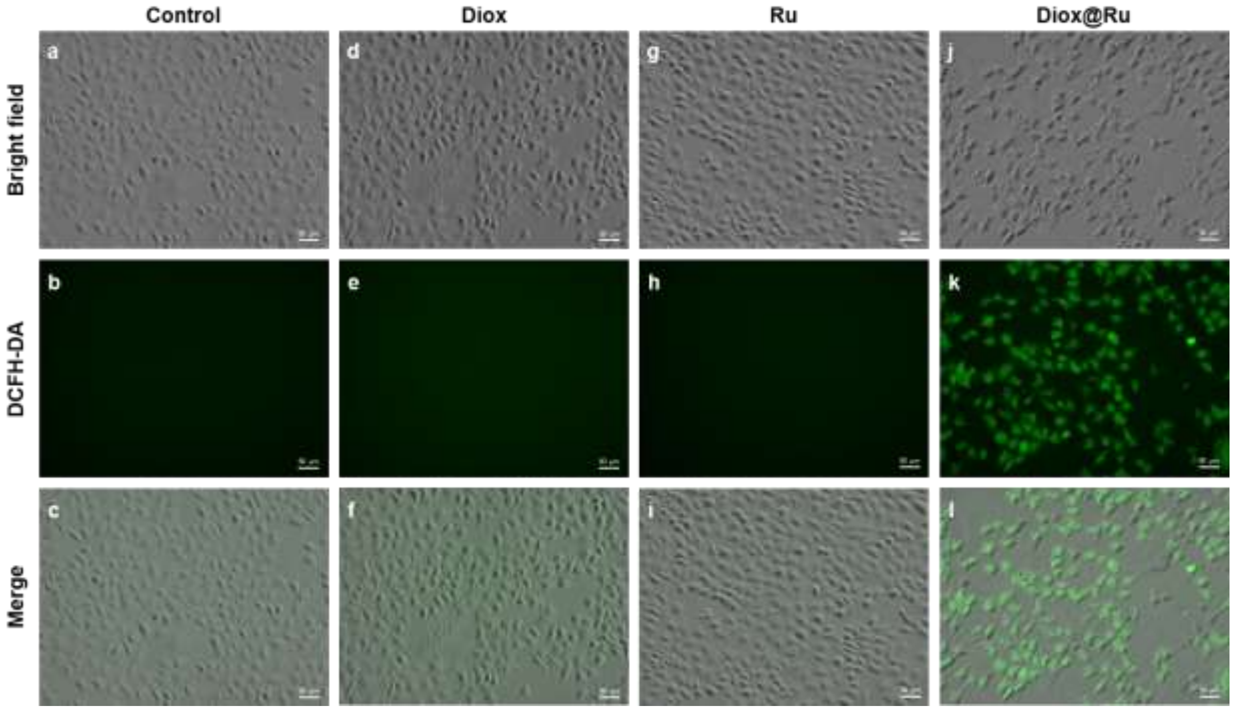

Figure S21. Intracellular ROS generation of (a-c) Control, (d-f) Diox, (g-i) Ru and (j-l) Diox@Ru with A549 cells (50 μM, 1% DMSO, incubating 8 h, DCFH-DA as indicator). Scale bar: 50 μm.

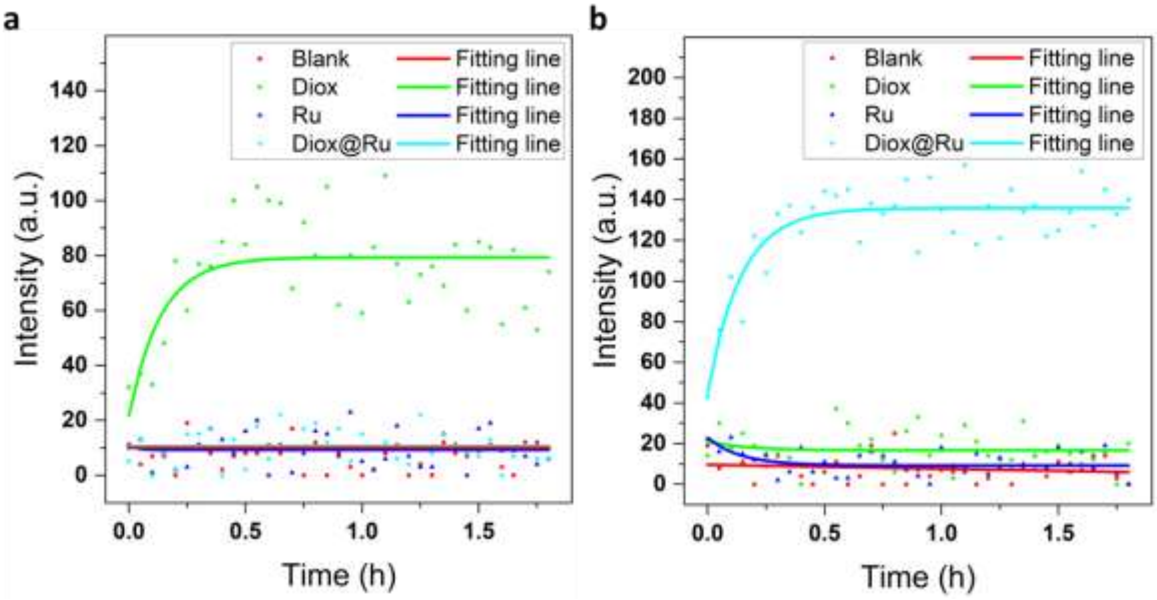

Figure S22. Intracellular chemiluminescence kinetic profiles of Diox@Ru in A549 cells at (a) 460-500 nm and (b) 580-700 nm (50 μM, 1% DMSO, incubating 4 h, the solid line is the fitting line.).

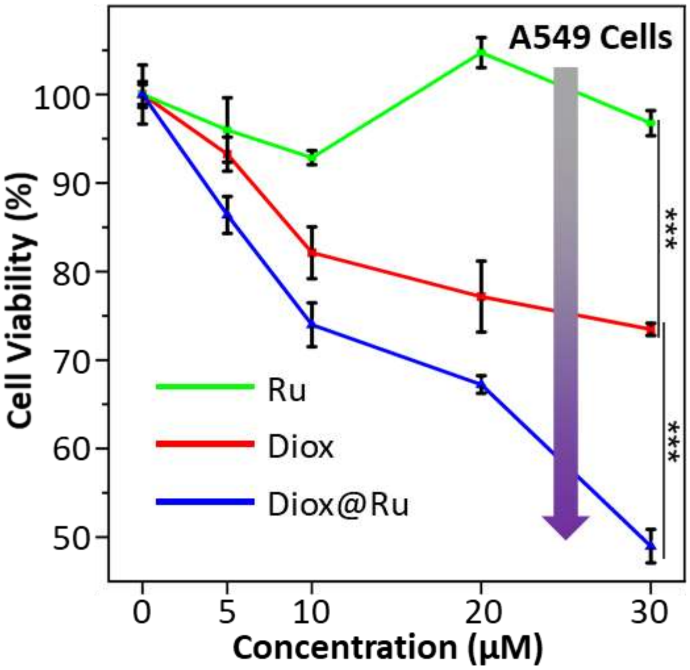

Figure S23. Cell viability of A549 cells (< 1% DMSO, incubating 24 h, CellTiterGlo luminescent cell viability assay as an indicator). Data presented as S.E.M, n = 5. Statistical significance was calculated by ANOVA with a Tukey post hoc test. *p < 0.05, **p < 0.01, ***p < 0.001

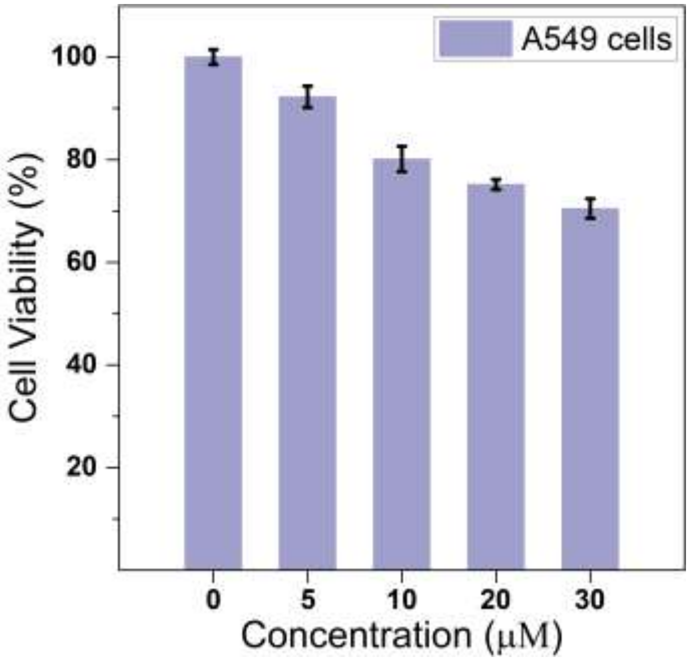

Figure S24. Cell viability of A549 cells with mixture of Diox and Ru (Diox-Ru) ((< 1% DMSO, incubating 24 h, CellTiterGlo luminescent cell viability assay as an indicator). Data presented as S.E.M, n = 5.

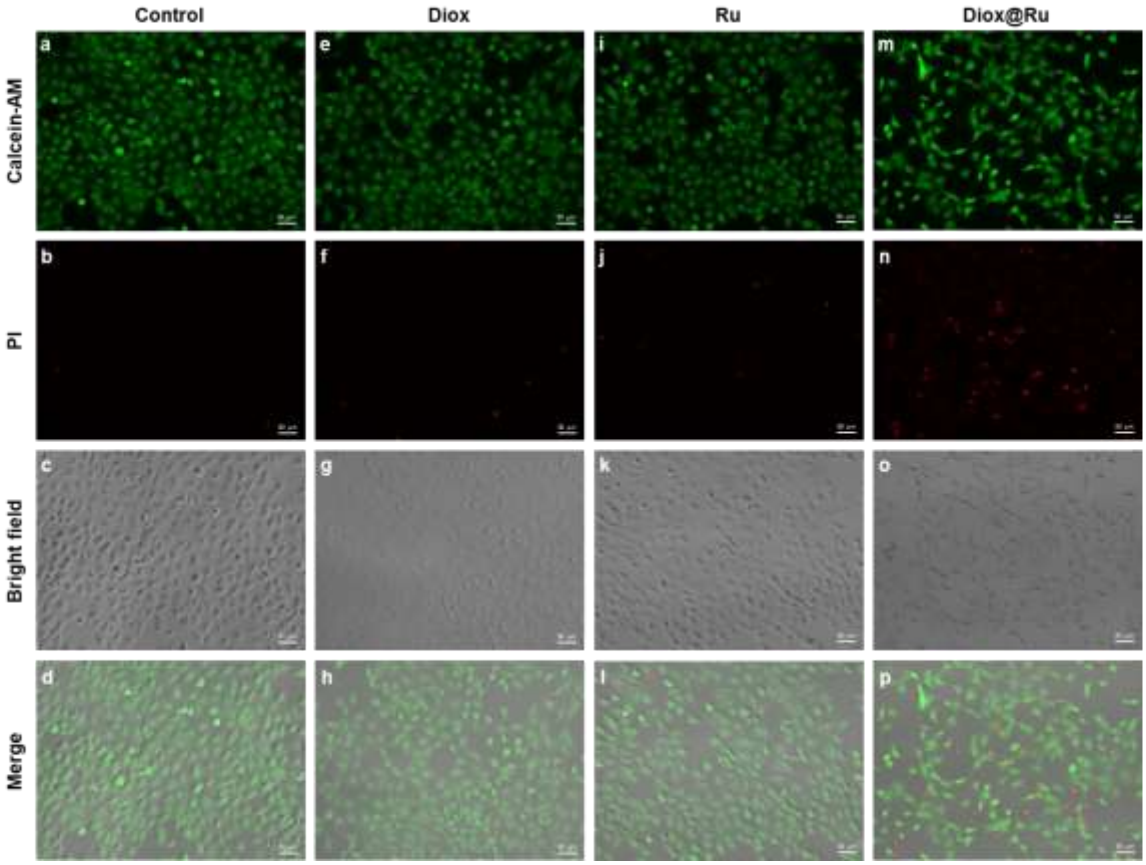

Figure S25. Live/Dead cell viability of (a-d) Control, (e-h) Diox, (i-l) Ru and (m-p) Diox@Ru with A549 cells (50 μM, 1% DMSO, incubating 8 h, Calcein-AM/PI as indicator). Scale bar: 50 μm.

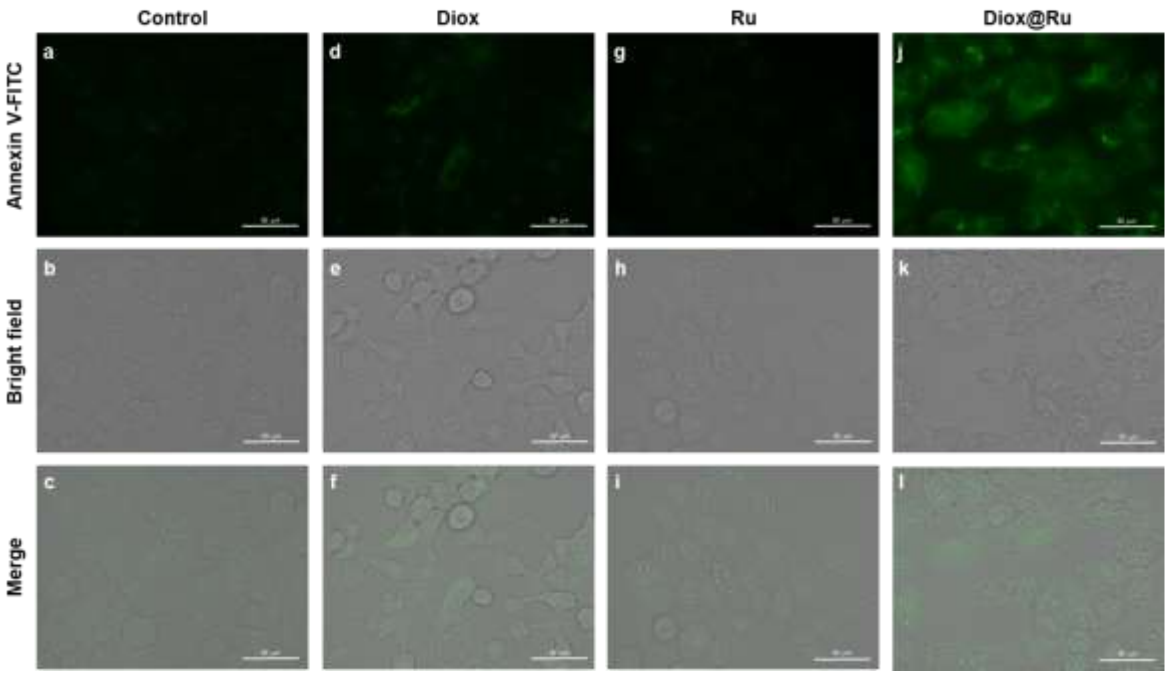

Figure S26. Cell apoptosis of (a-c) Control, (d-f) Diox, (g-i) Ru and (j-l) Diox@Ru with A549 cells (50 μM, 1% DMSO, incubating 8 h, Annexin V-FITC as indicator). Scale bar: 50 μm.

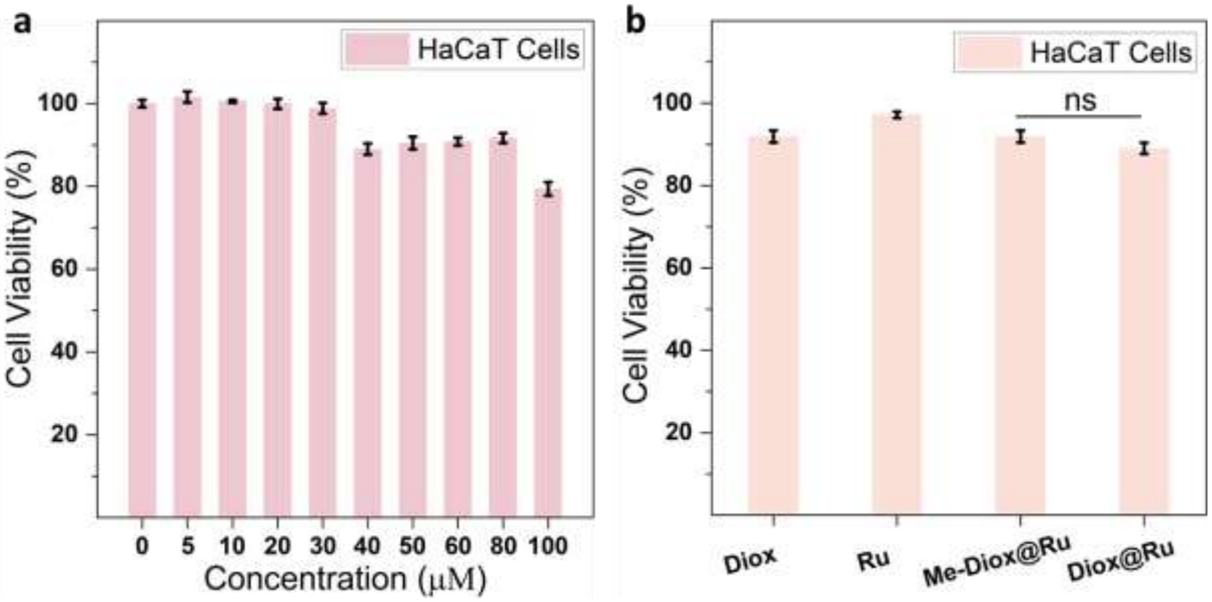

Figure S27. Cell viability of (a) Me-Diox@Ru (0 - 100 μM) and (b) comparisons of Me-Diox@Ru with Diox, Ru and Diox@Ru at identical concentrations (40 μM) in HaCaT cells. (< 2% DMSO, incubating 24 h, CellTiterGlo luminescent cell viability assay as an indicator). Data presented as S.E.M, n = 5. Statistical significance was calculated by ANOVA with a Tukey post hoc test. ns, not significant.

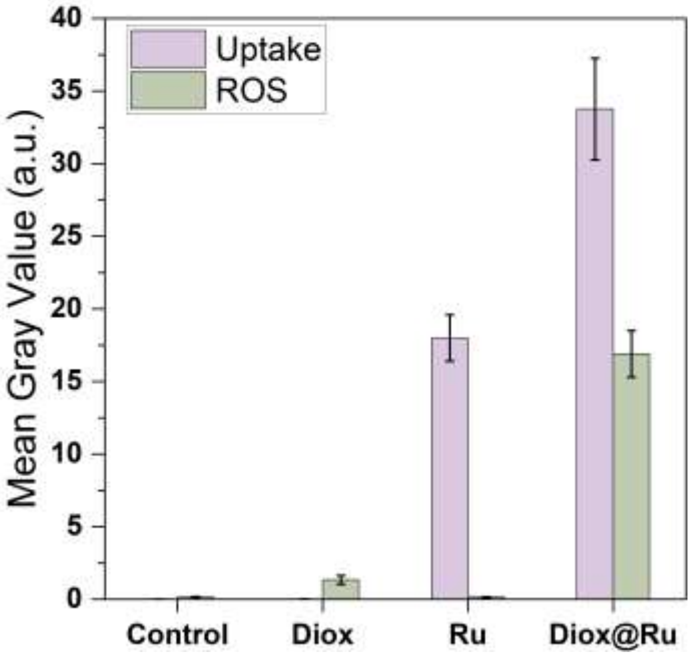

Figure S28. Mean gray value of Diox, Ru and Diox@Ru on internalization and ROS with 3D tumor spheroids of 4T1 cells. Mean gray value = Total fluorescence intensity/Area. Data presented as S.E.M, n = 3.

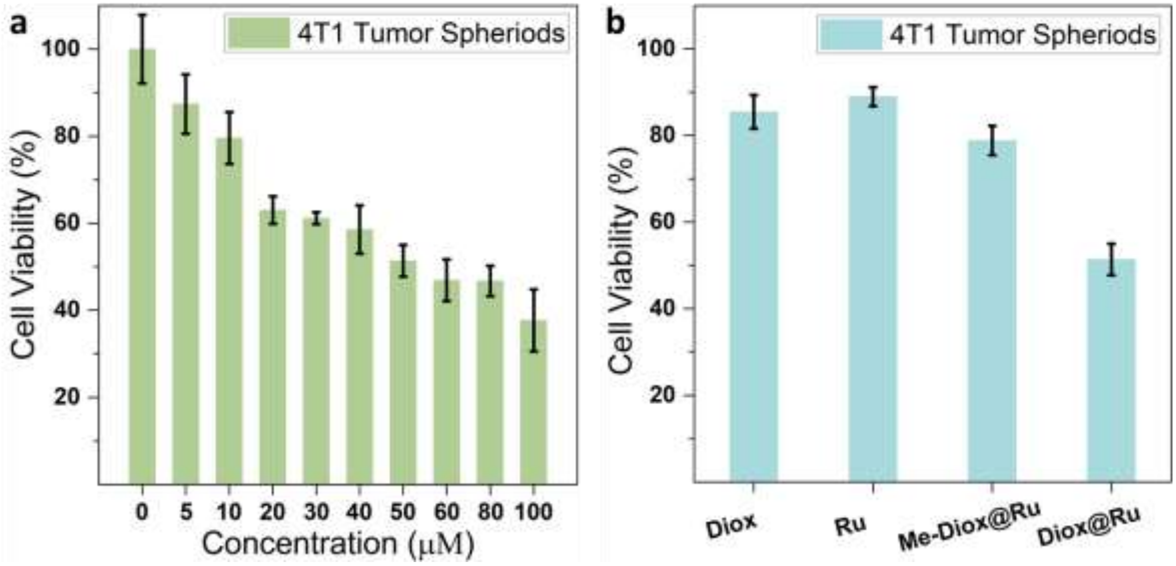

Figure S29. Cell viability of (a) Diox@Ru (0 - 100 μM) and (b) comparisons of Diox@Ru with Diox, Ru and Me-Diox@Ru at identical concentrations (50 μM) in 3D tumor spheroids of 4T1 cells. (< 2% DMSO, incubating 24 h, CellTiter-Glo® 3D cell viability assay as an indicator). Data presented as S.E.M, n = 5.

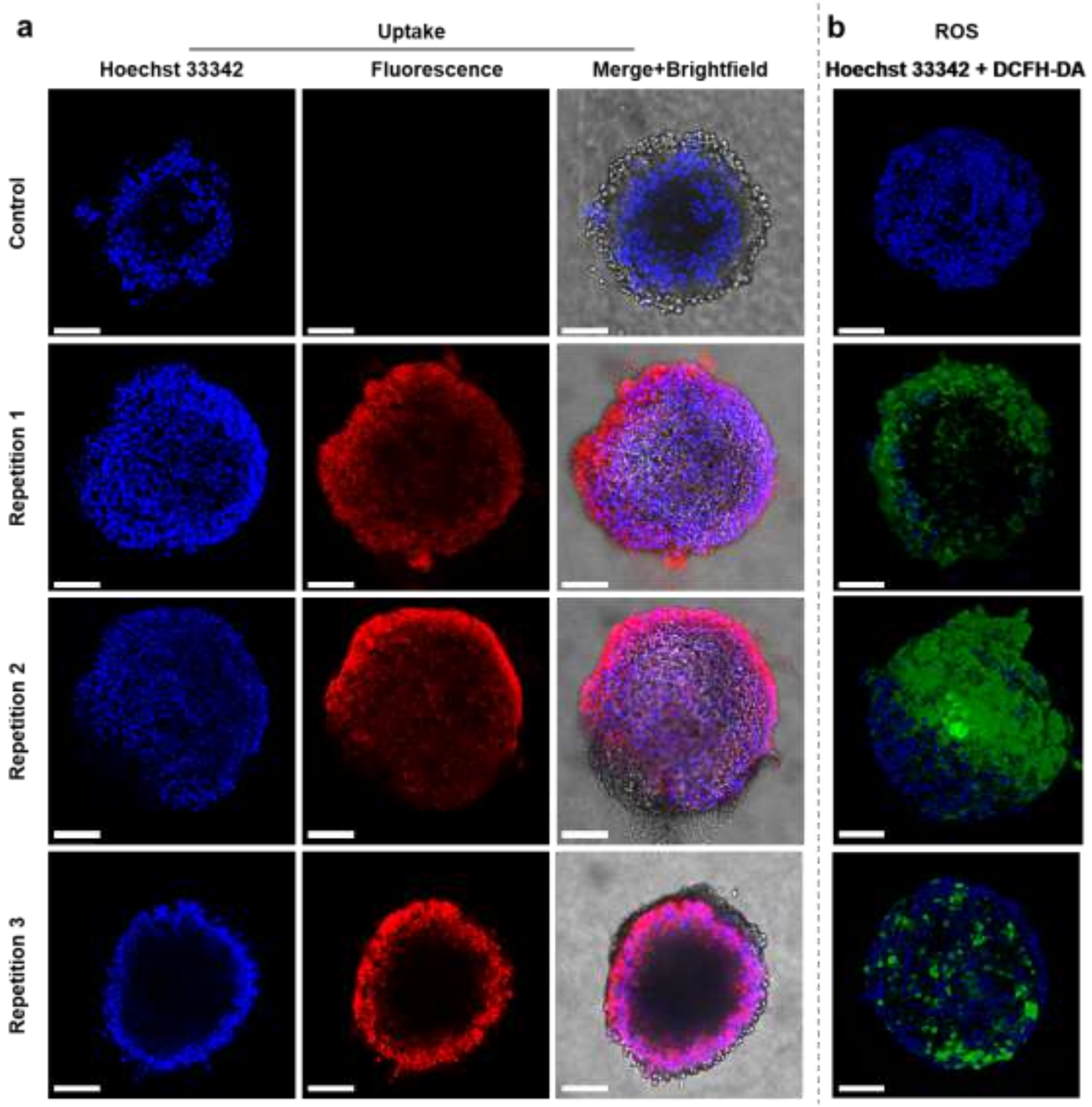

Figure S30. Independent experiments (replicates) performed for (a) cellular uptake and (b) ROS production of Diox@Ru in 4T1 tumor spheroids (50 μM, 1% DMSO, 24 h incubation). Scale bar: 100 μm.

3. Spectra of Compounds (^{1}H-NMR, ^{13}C-NMR, LC-MS)

Compound **1**

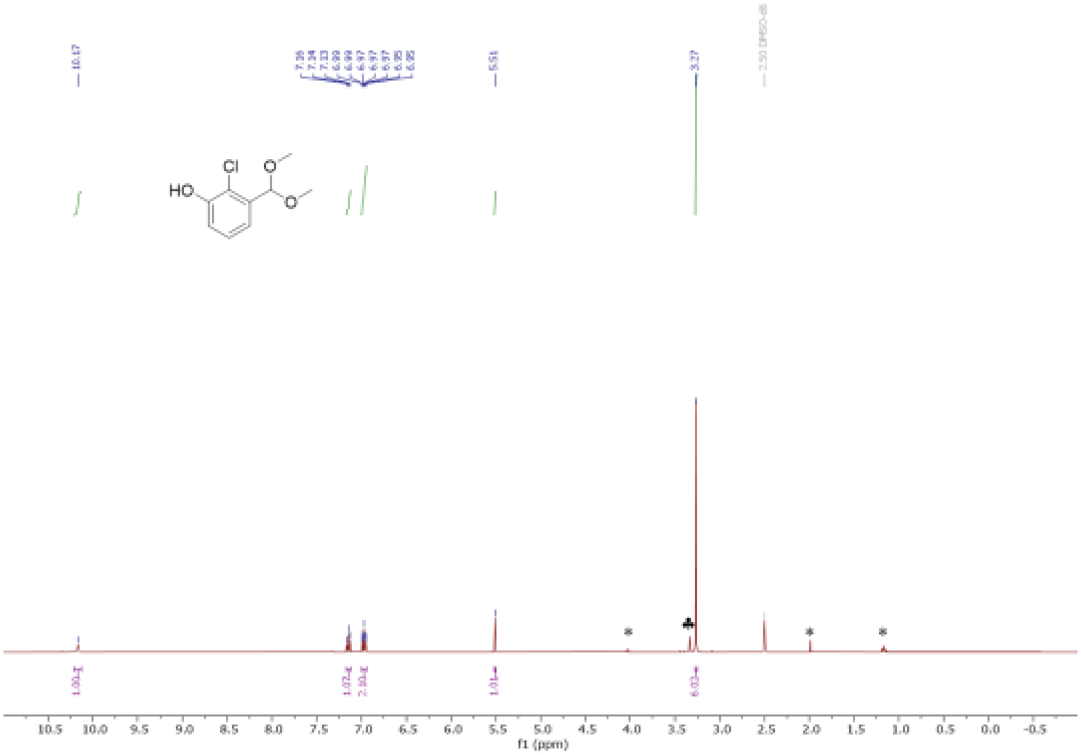

Solvent residual signals: ∗ ethyl acetate; ♣ water

Compound **2**

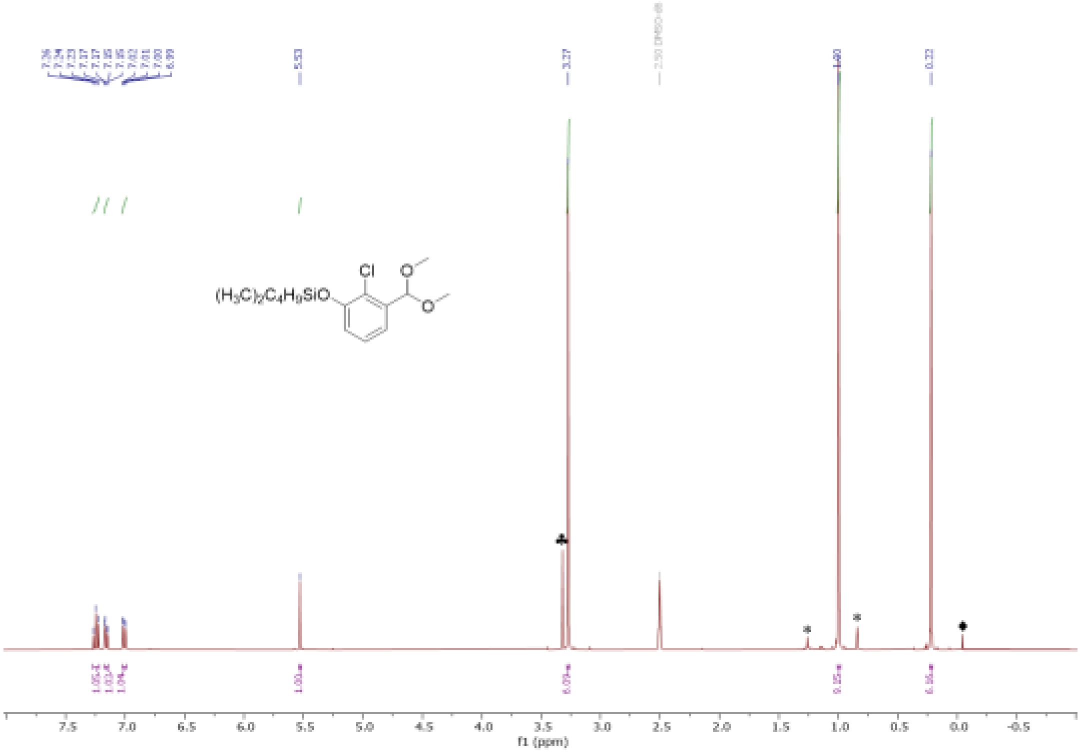

Solvent residual signals: ∗ n-hexane; ♣ water; ♦ silicone grease

Compound **3**

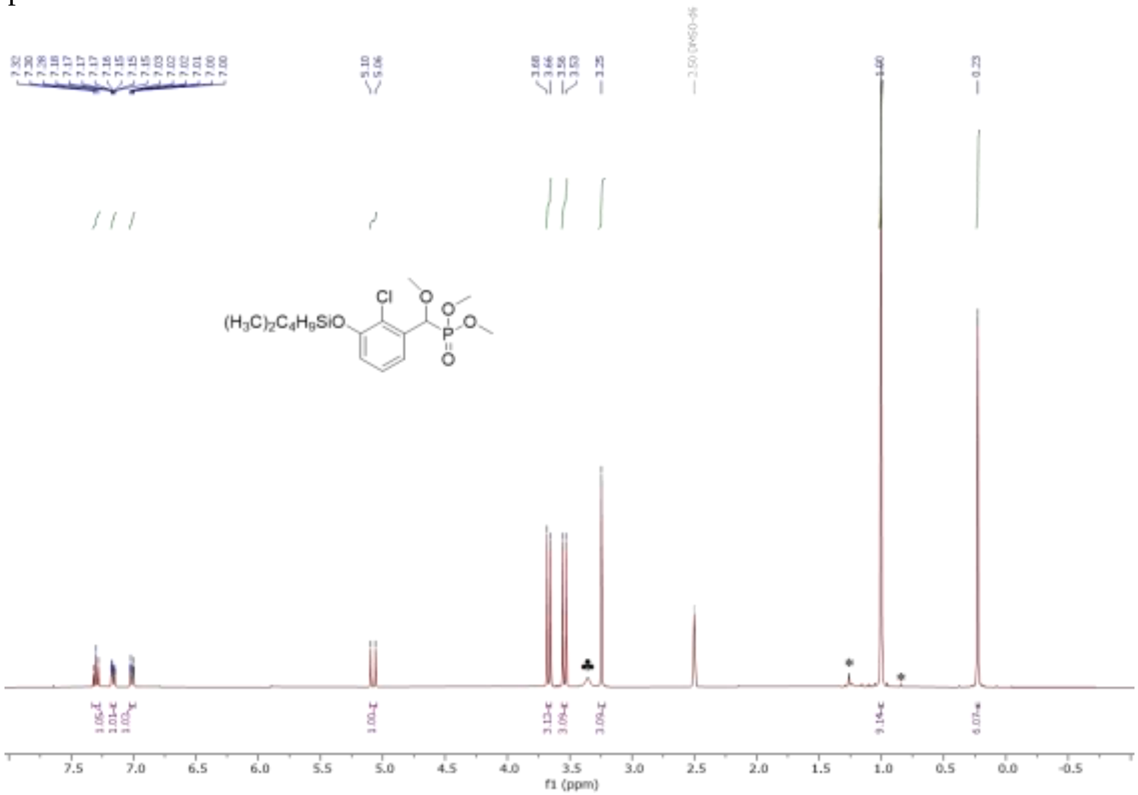

Solvent residual signals: ∗ n-hexane; ♣ water

Compound **4**

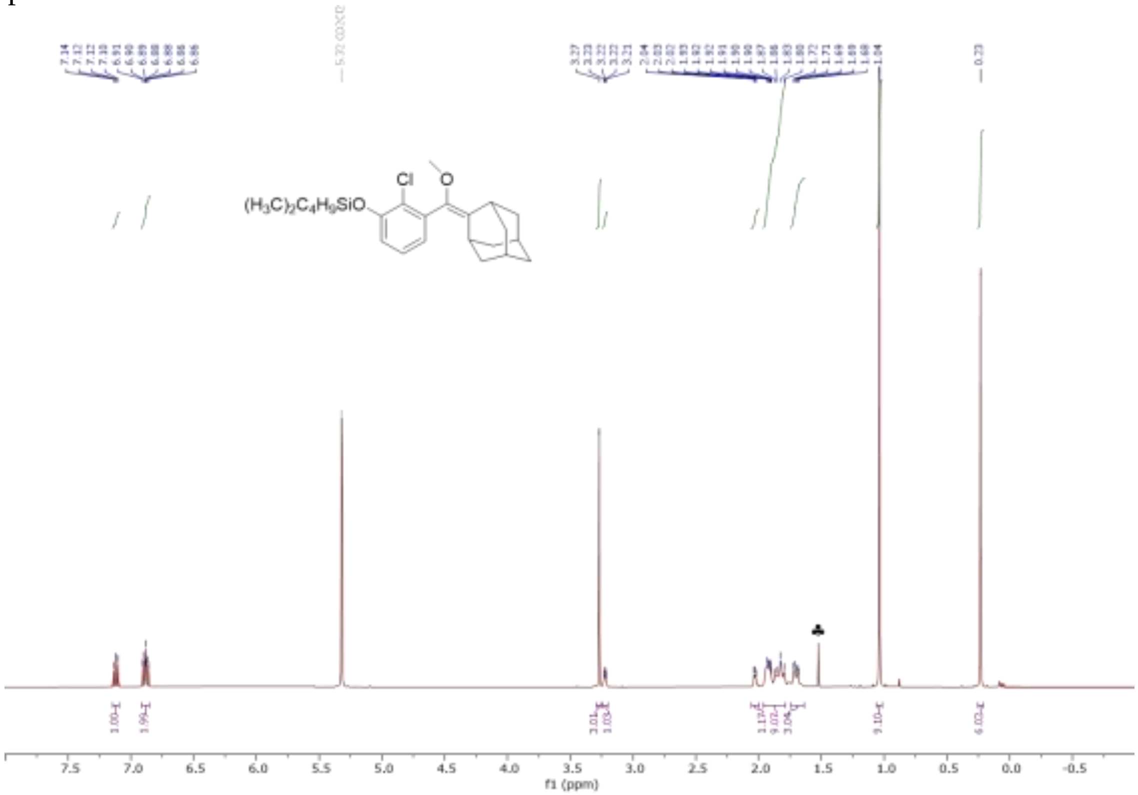

Solvent residual signals: ♣ water

Compound **5**

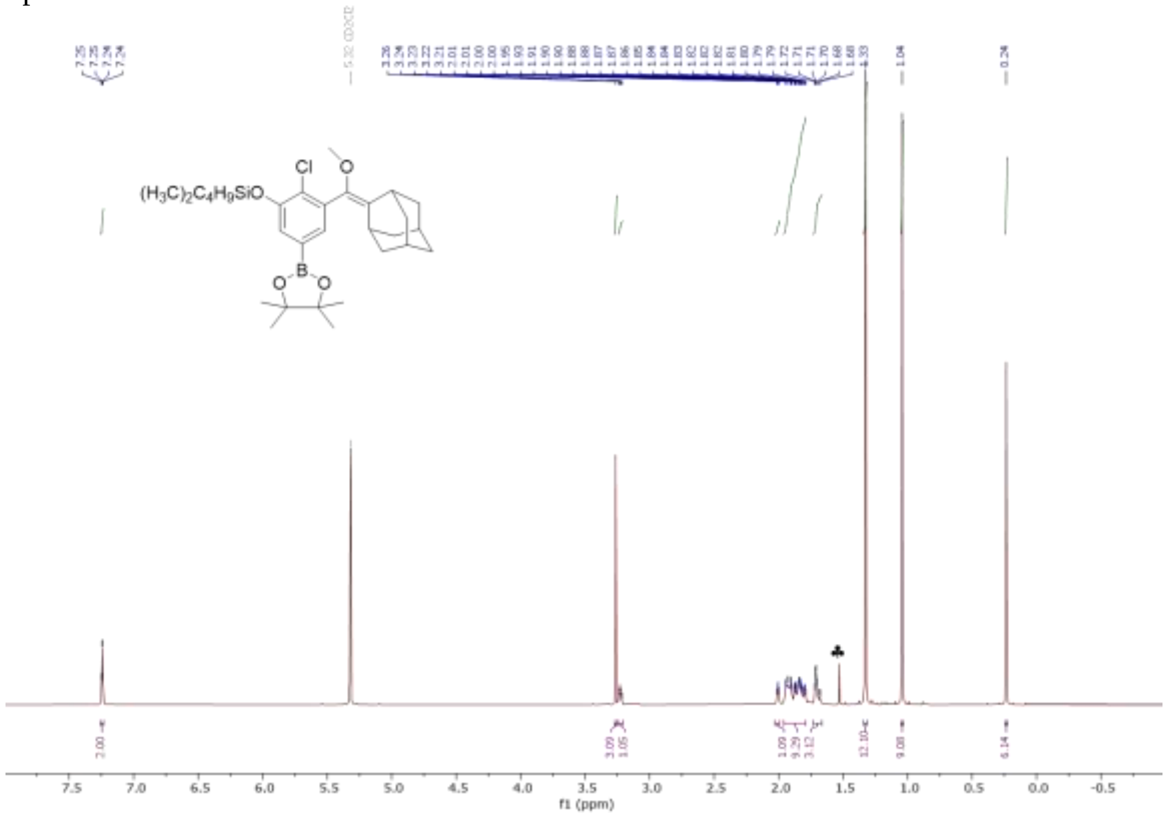

Solvent residual signals: ♣ water

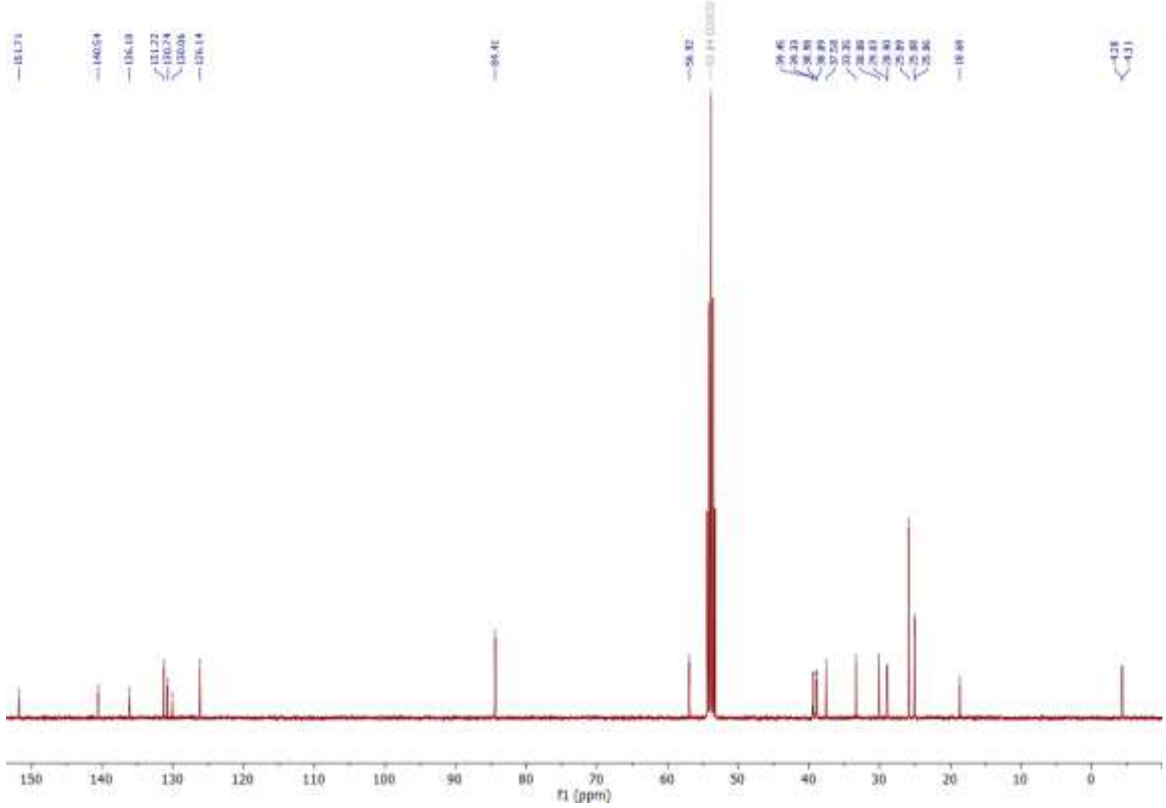

Compound **6**

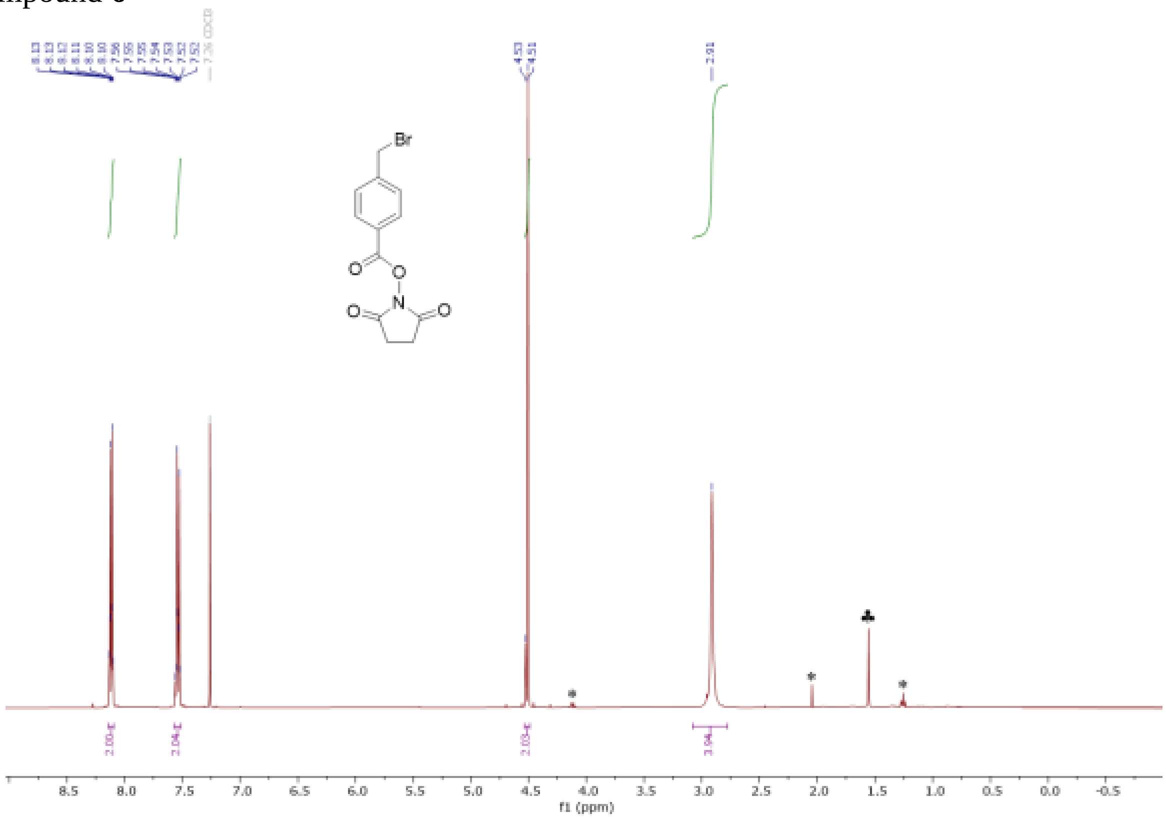

Solvent residual signals: ∗ ethyl acetate; ♣ water

Compound **7**

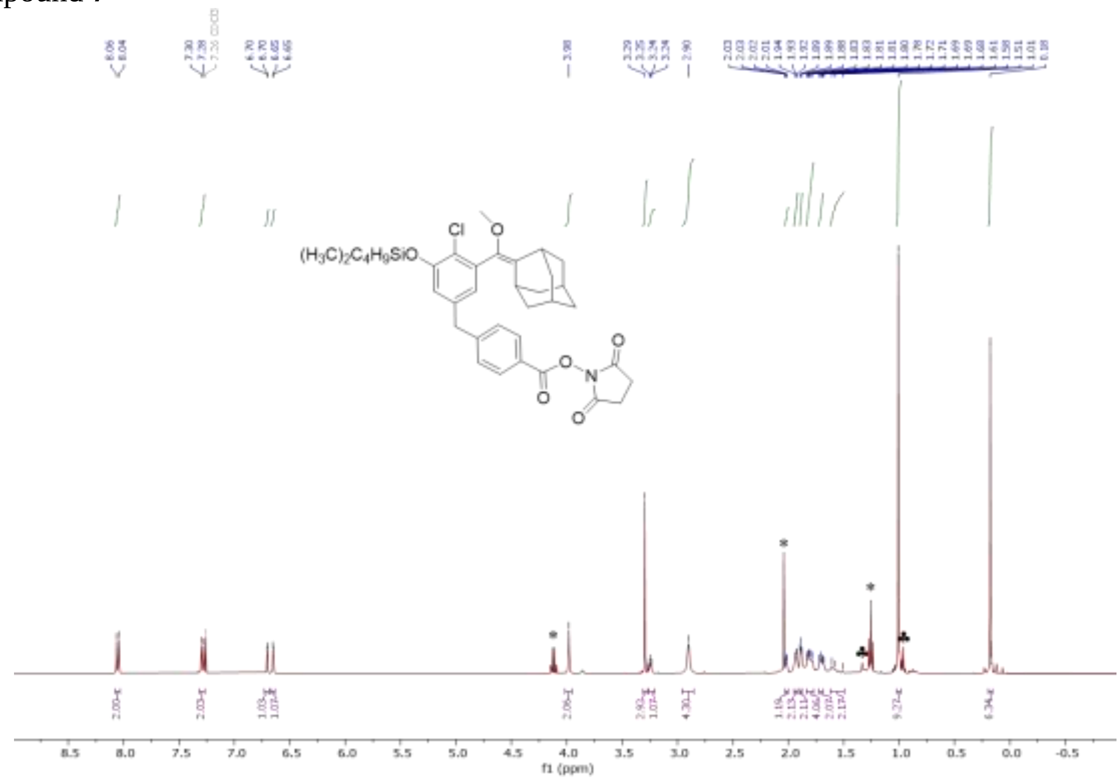

Solvent residual signals: ∗ ethyl acetate; ♣ n-hexane

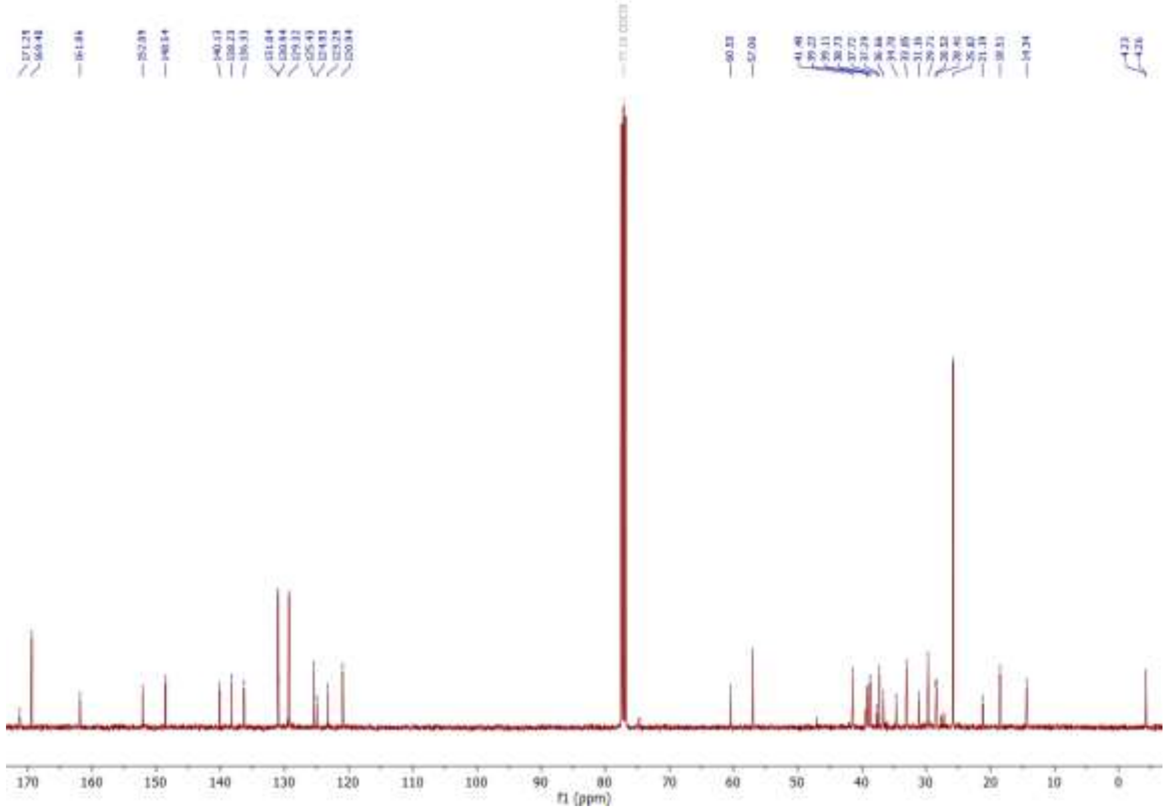

Compound **8**

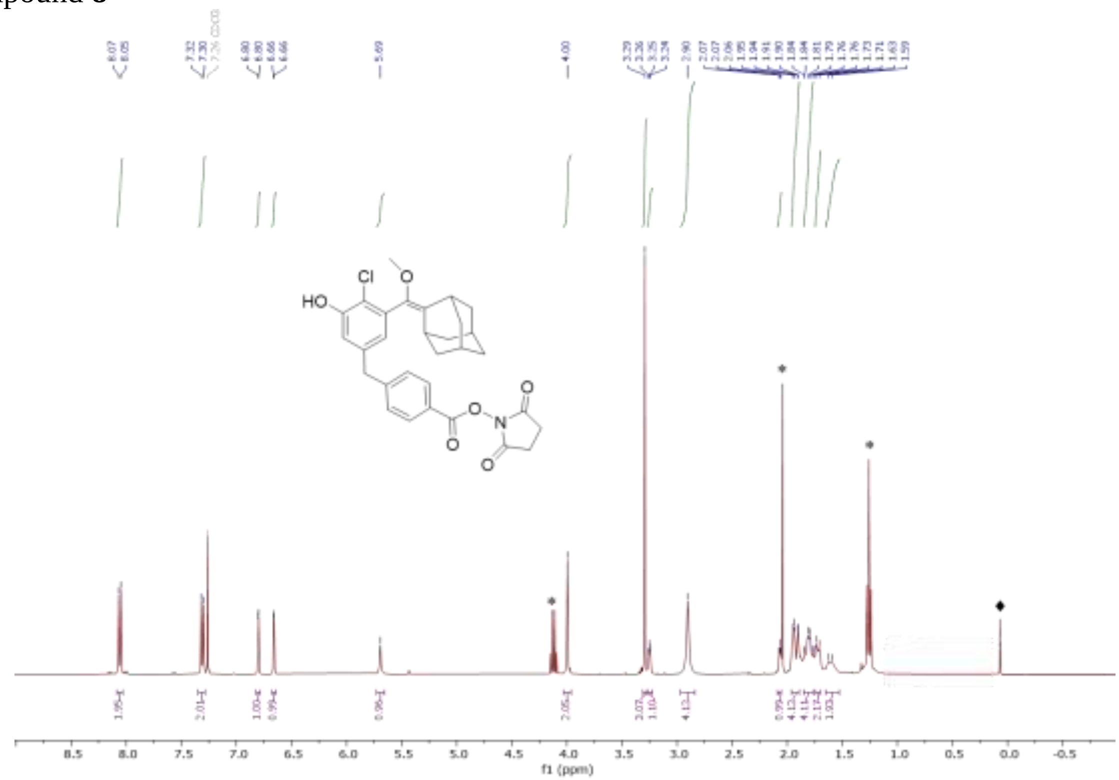

Solvent residual signals: ∗ ethyl acetate; ♦ silicone grease

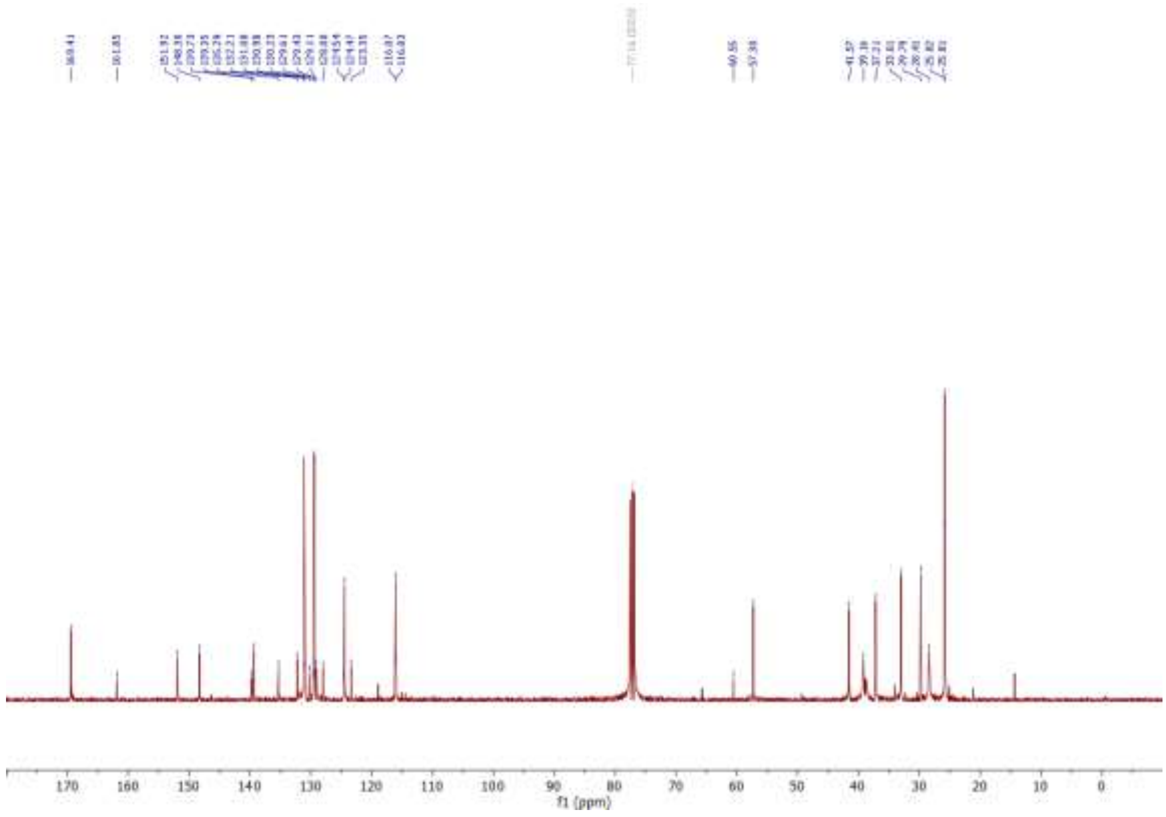

Compound **9**

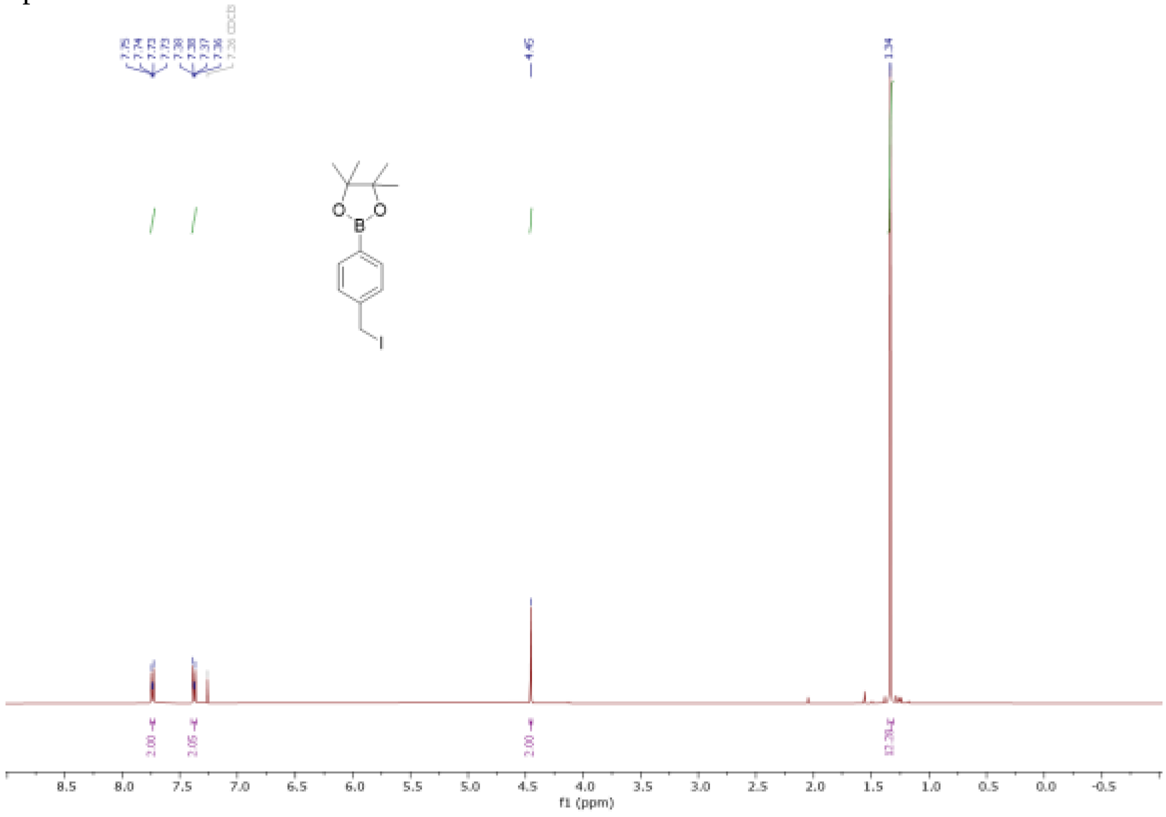

Compound **10**

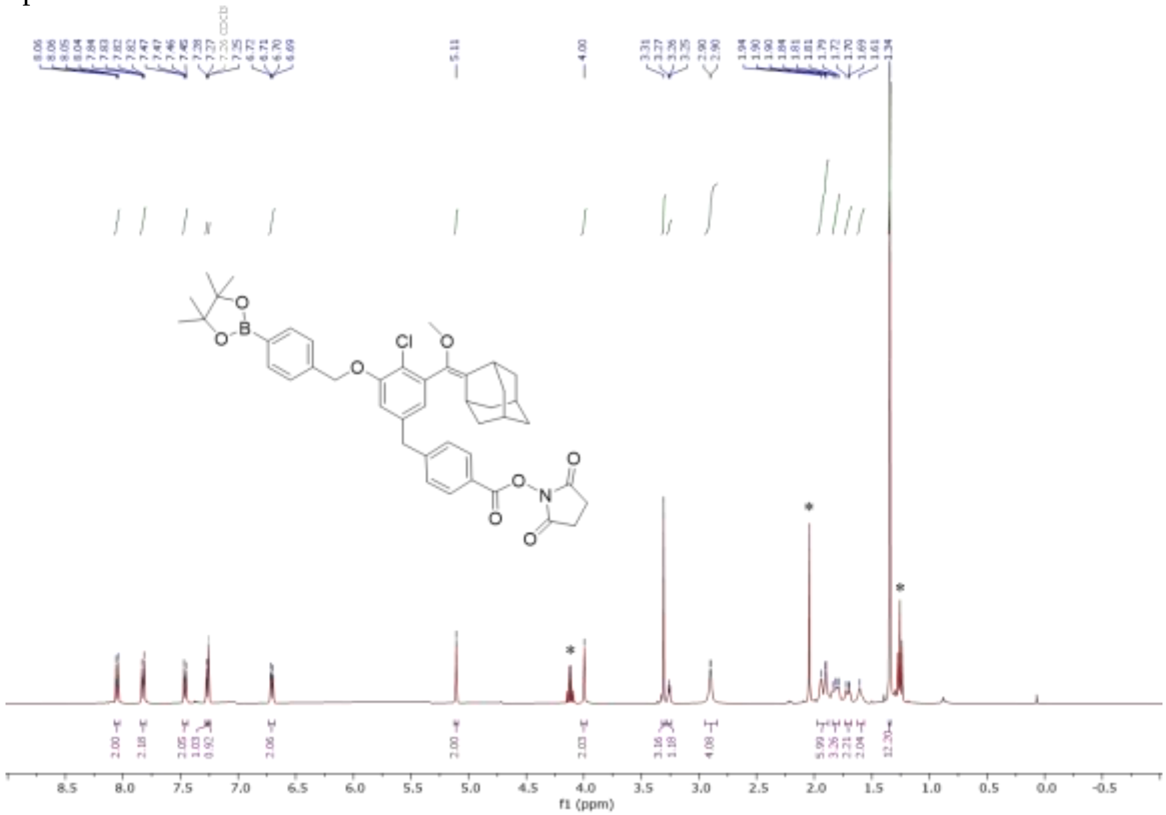

Solvent residual signals: ∗ ethyl acetate

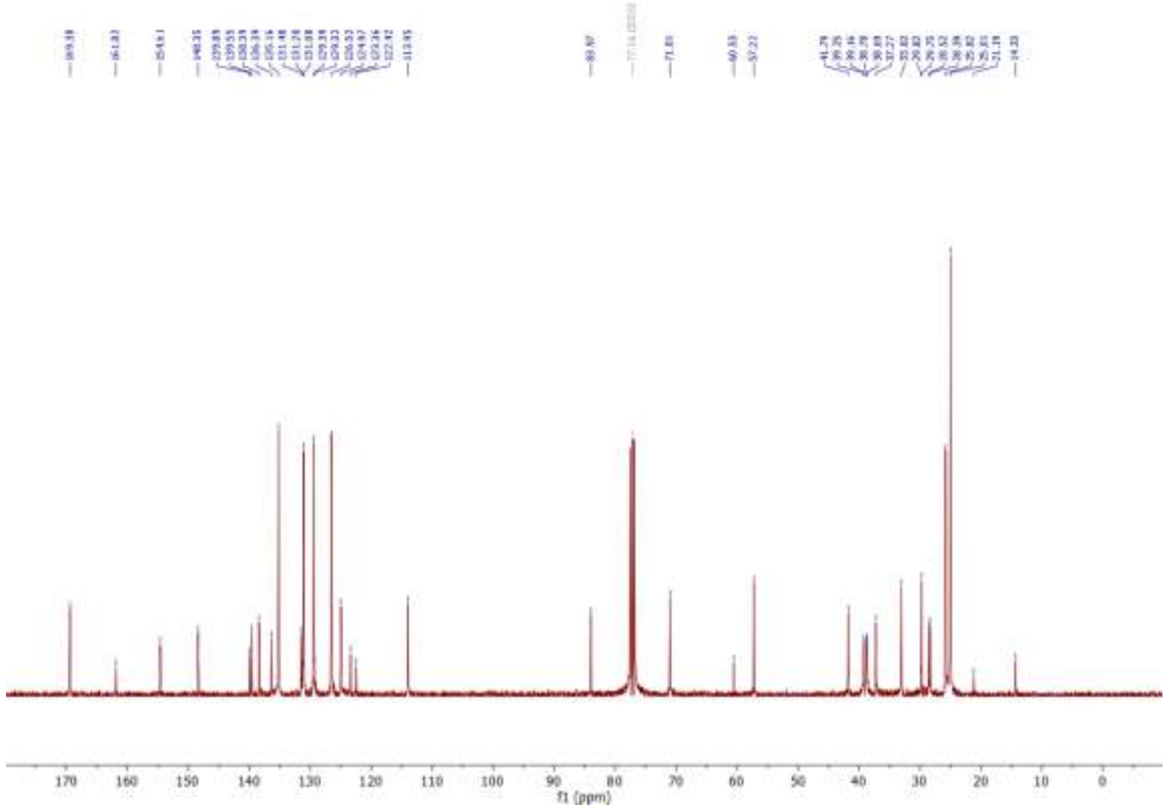

Compound **11-1**

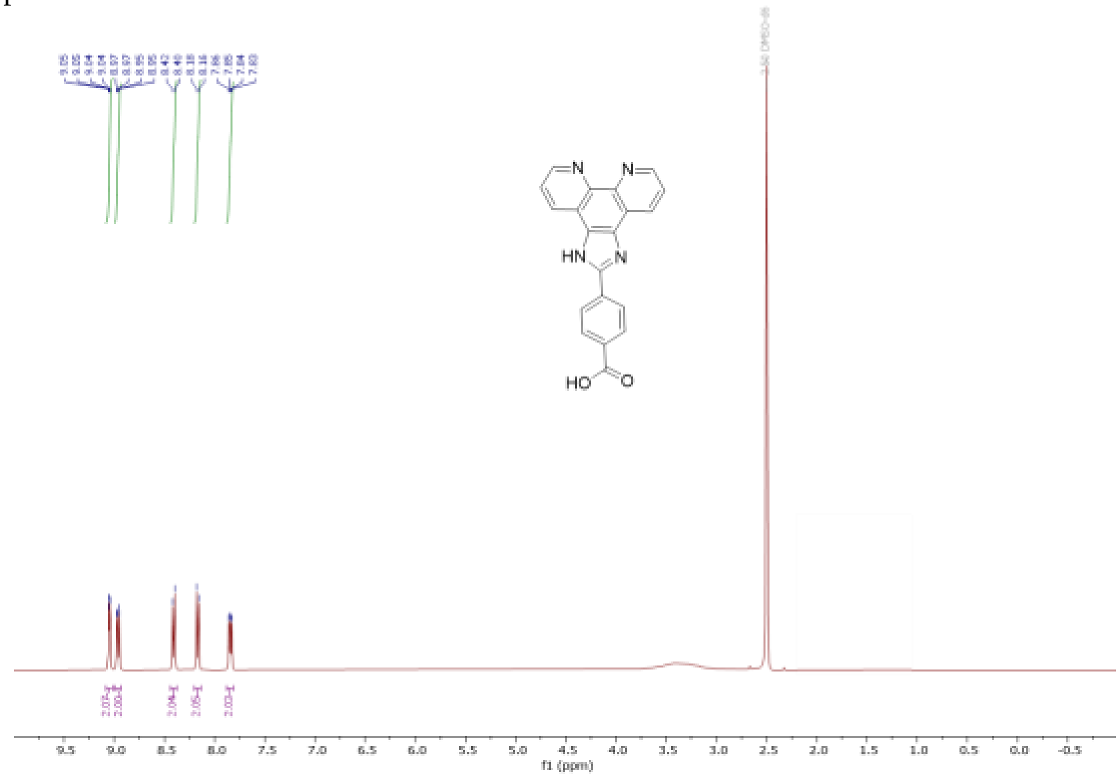

Compound **11-2**

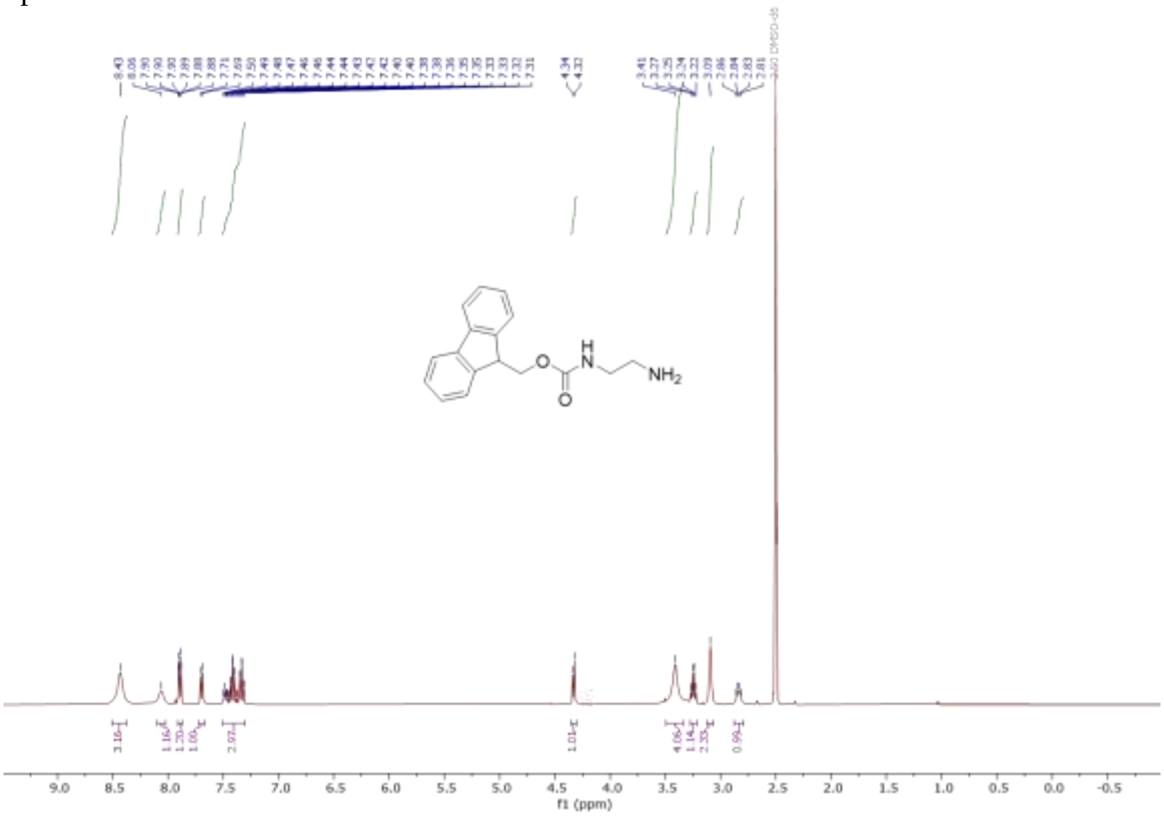

Compound **11-3**

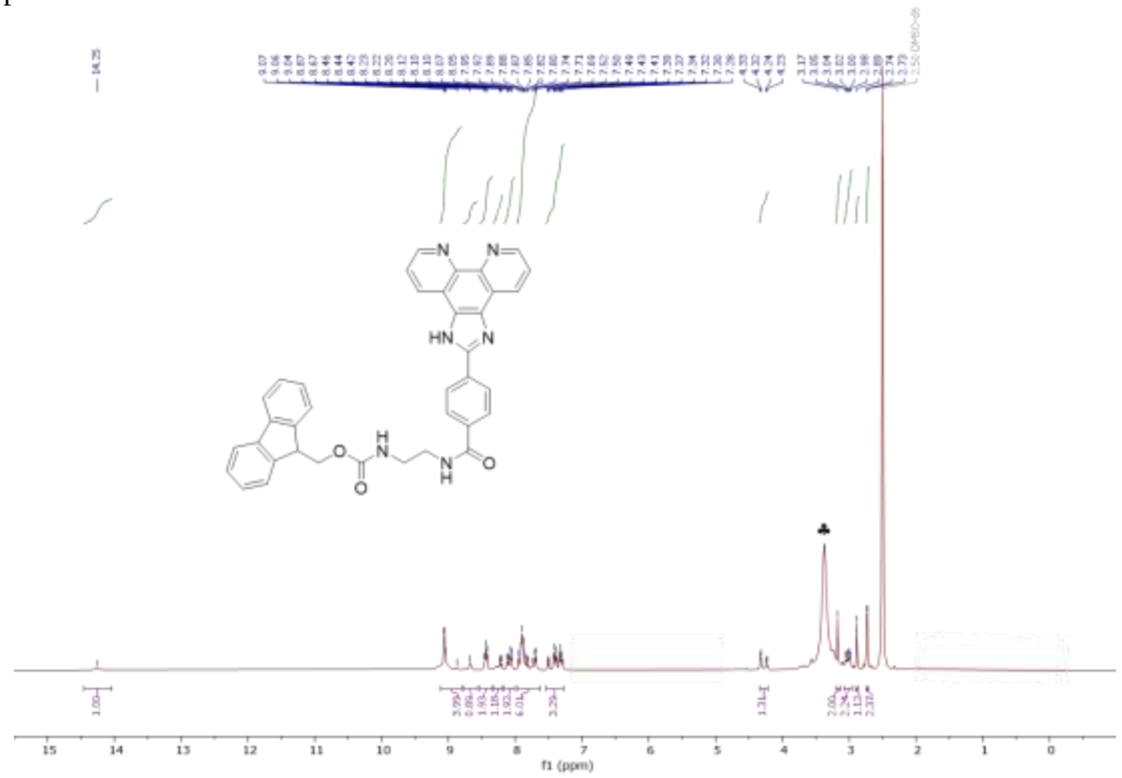

Solvent residual signals: ♣ water

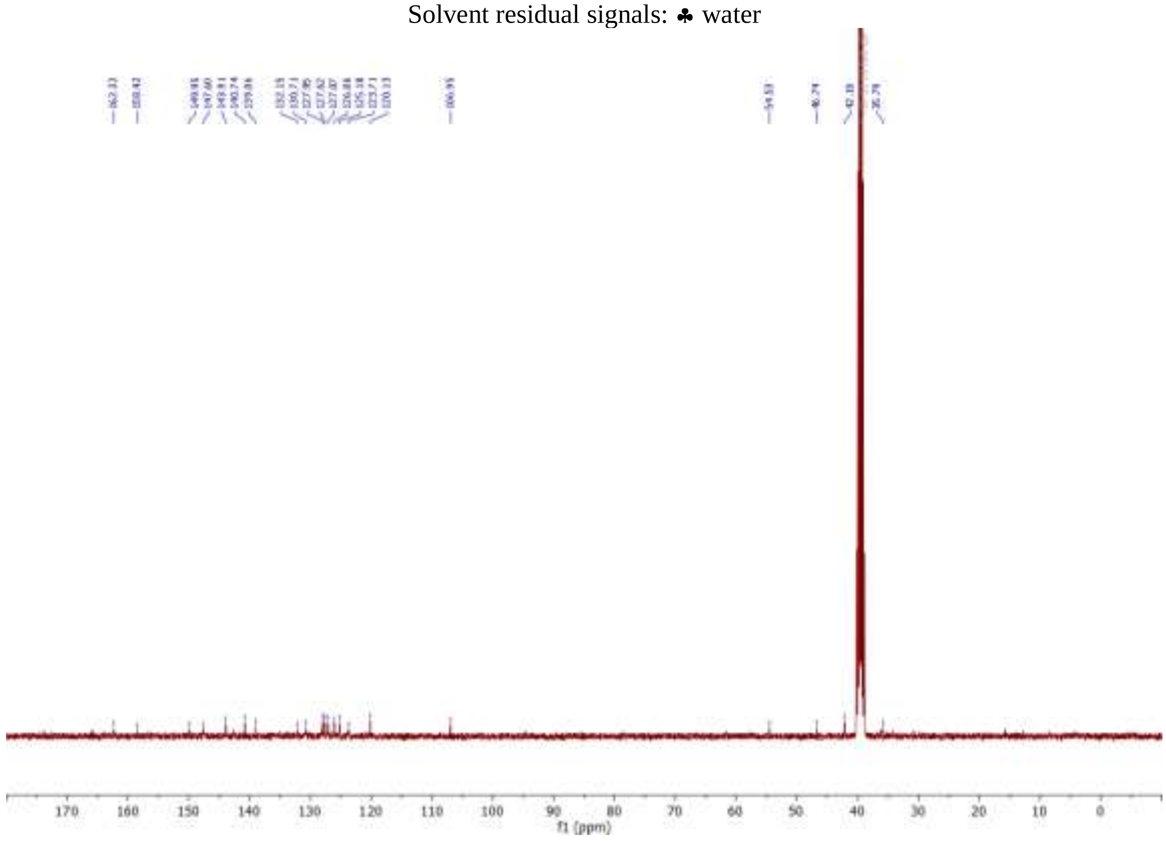

Compound **11**

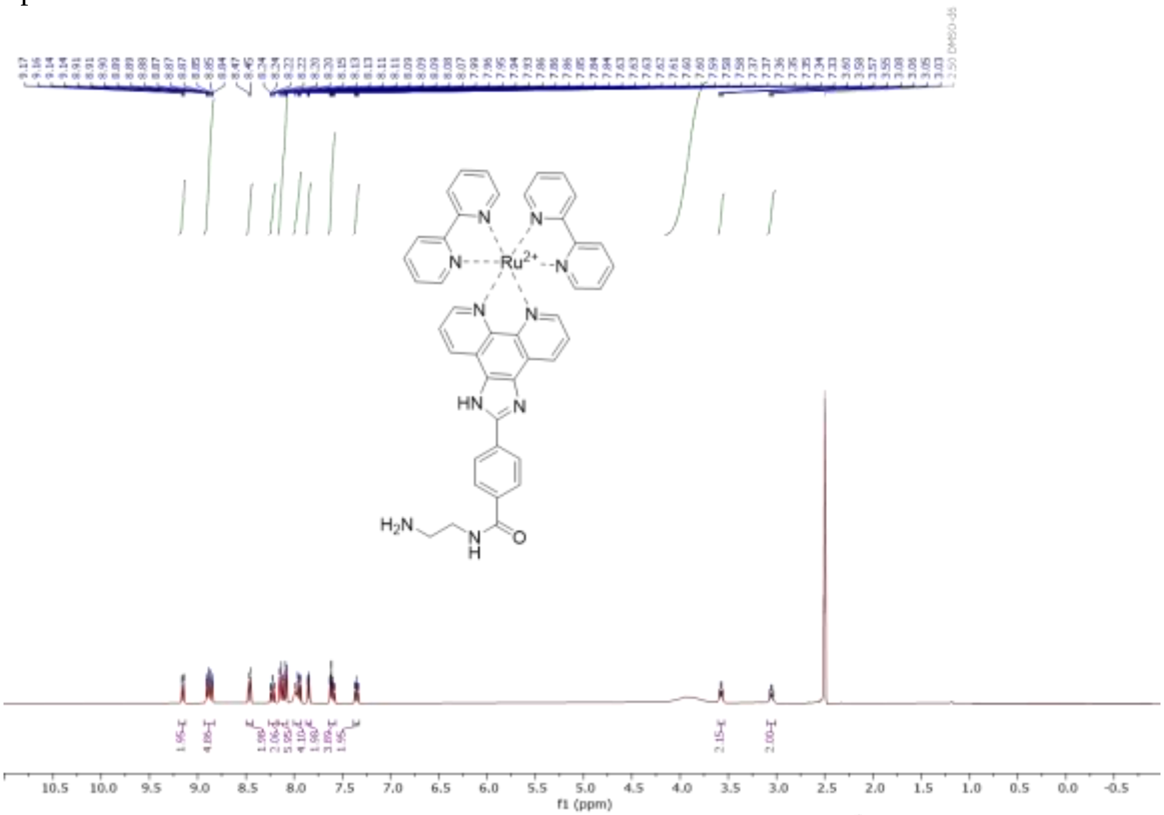

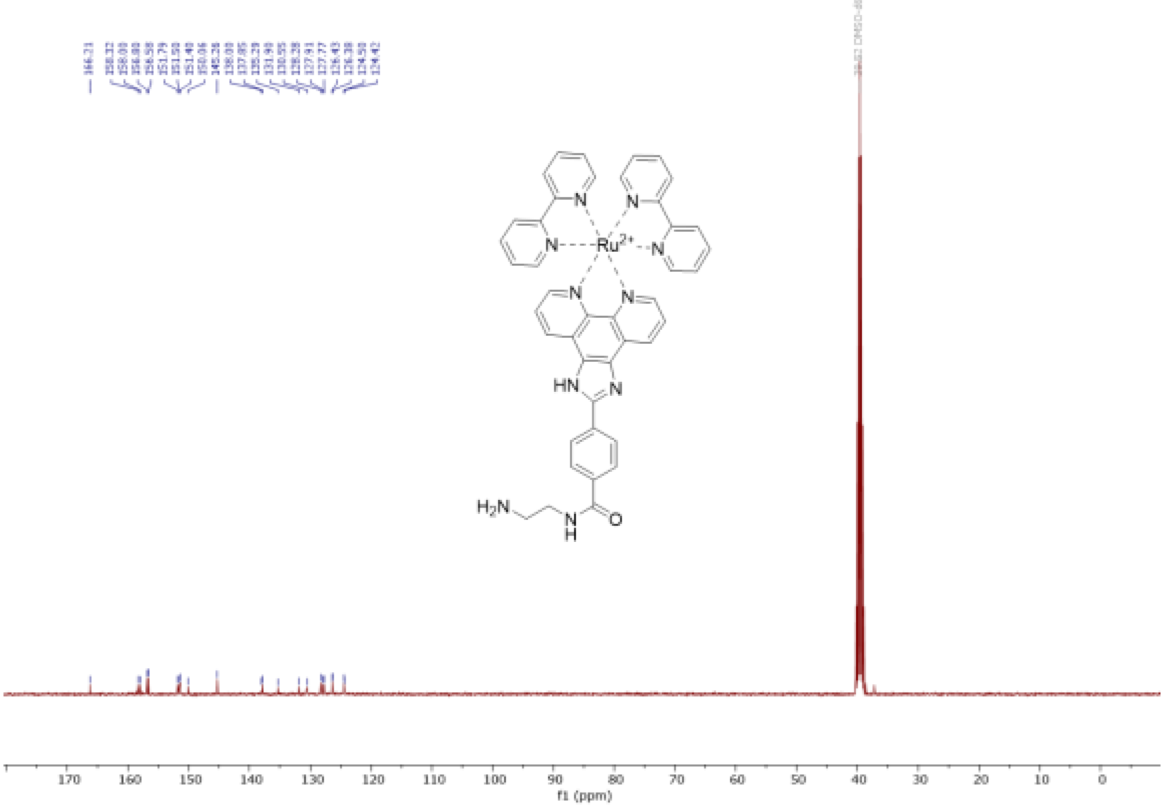

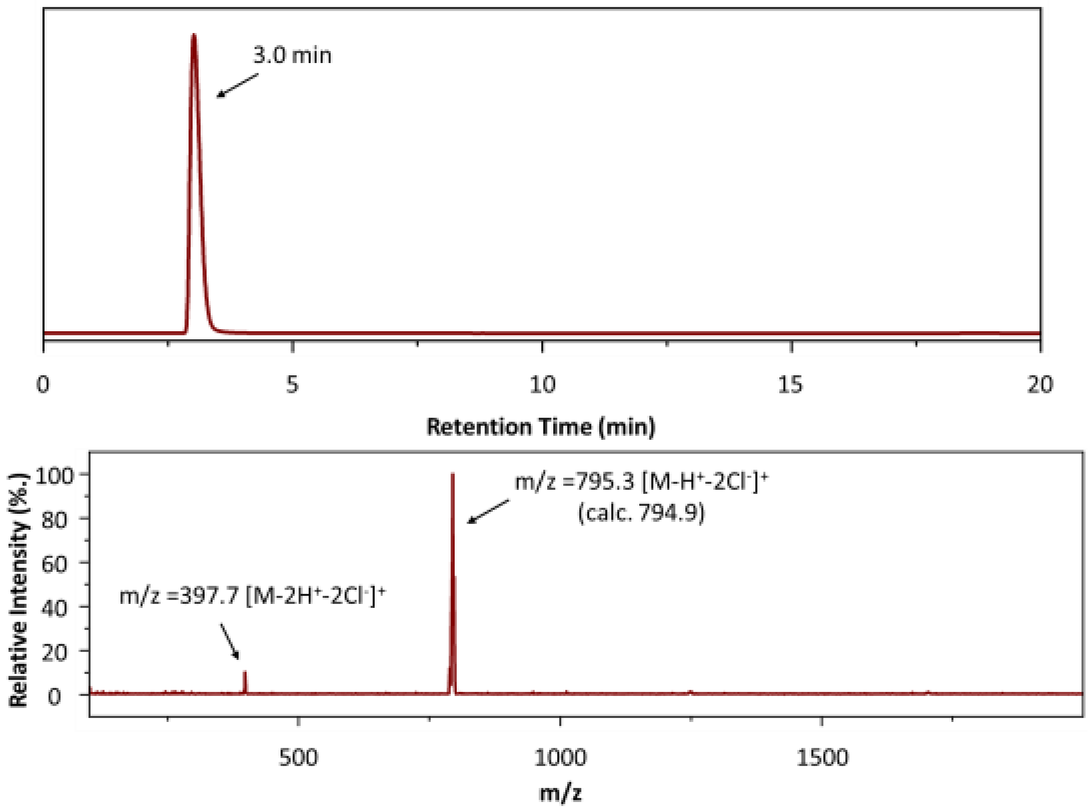

High-performance liquid chromatography - mass spectrometry of compound 11

Compound **12**

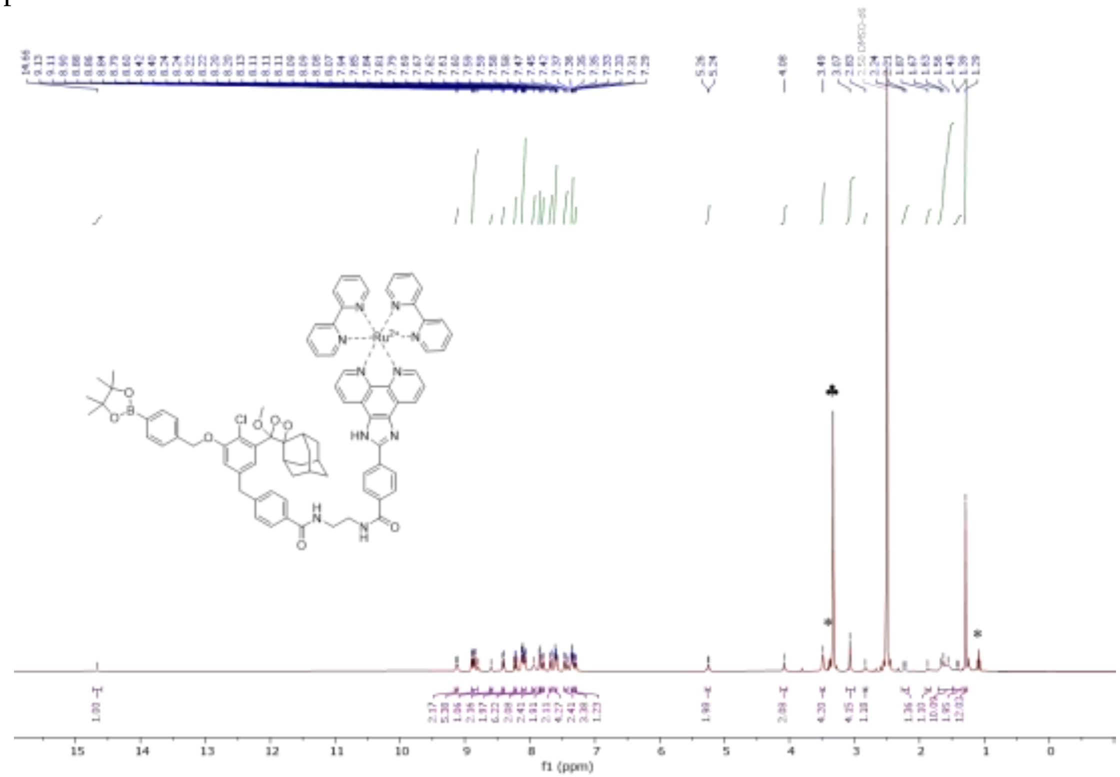

Solvent residual signals: ♣ water; ∗ diethyl ether

Compound **13**

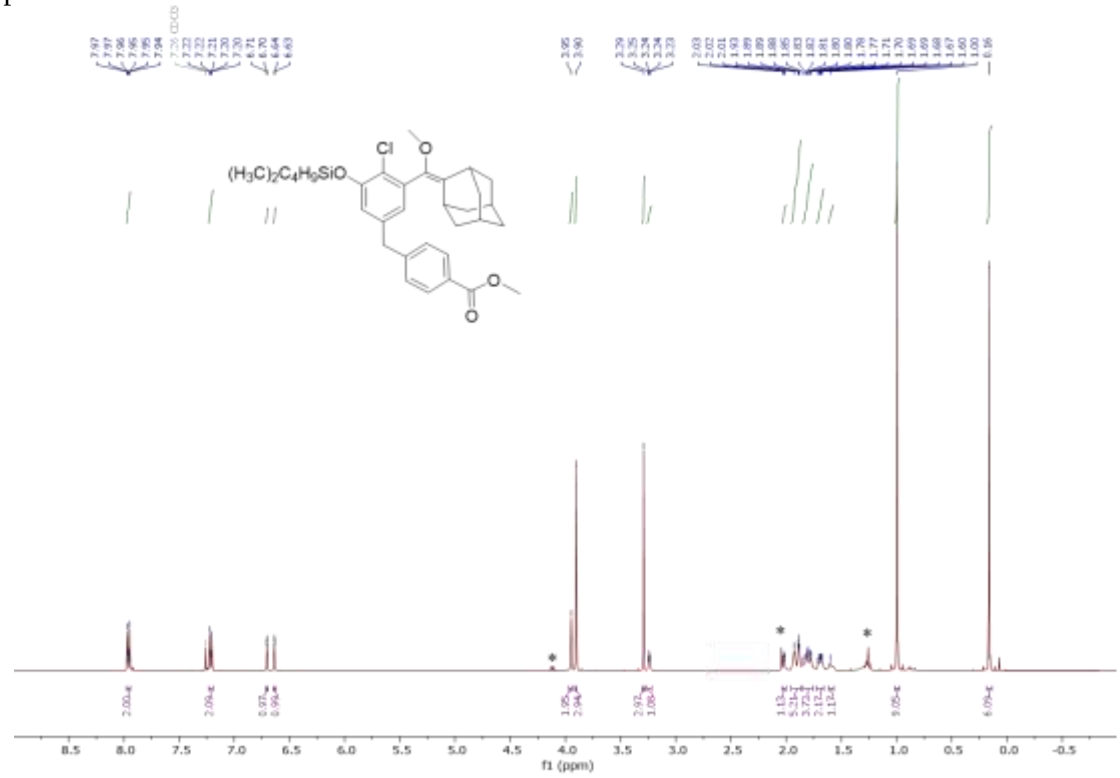

Solvent residual signals: ∗ ethyl acetate

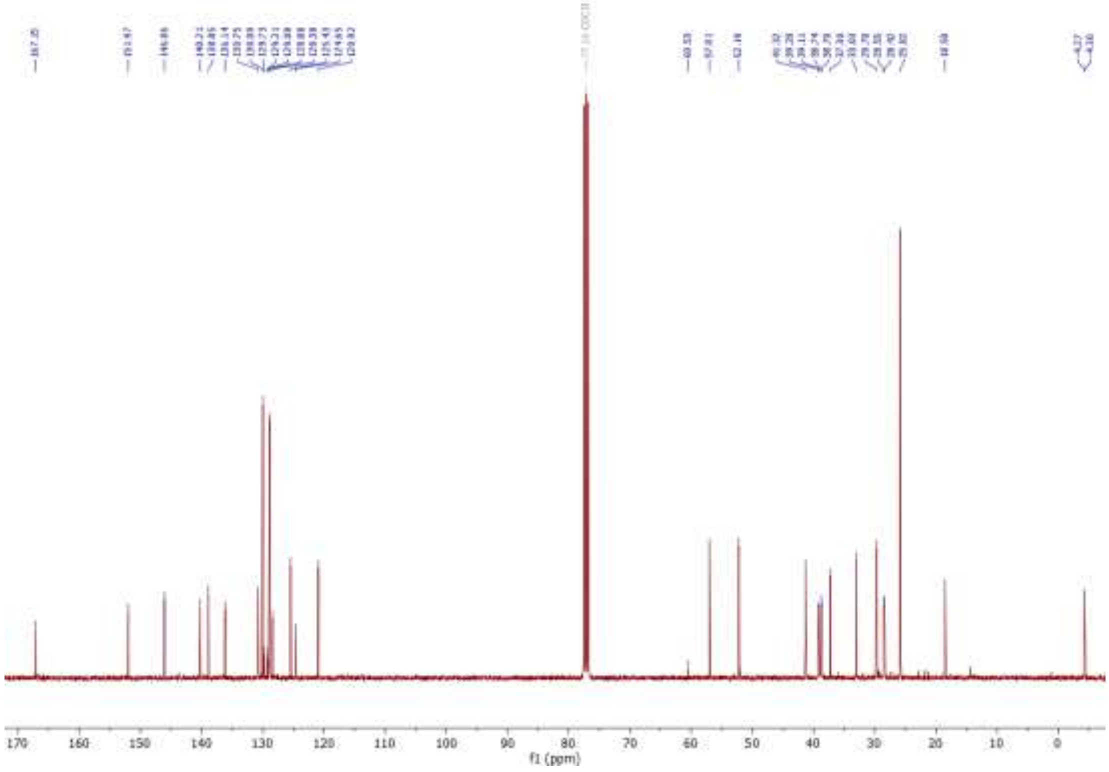

Compound **14**

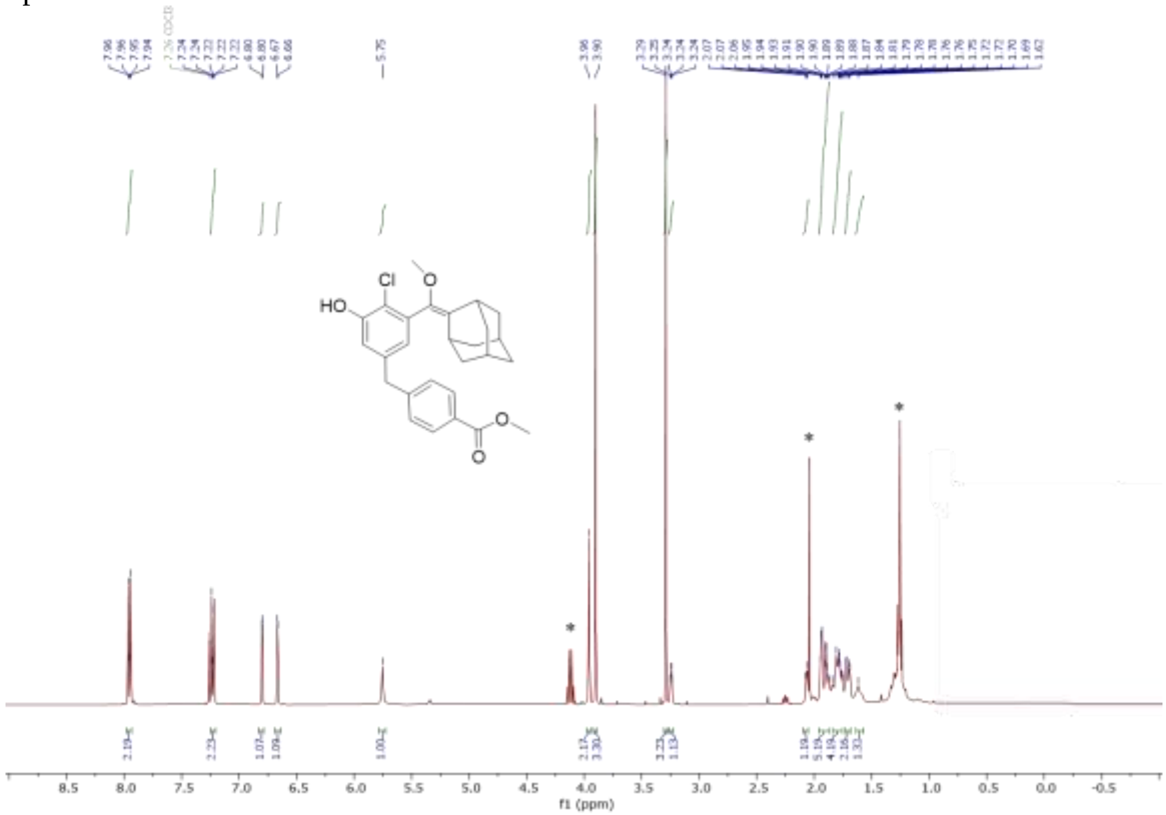

Solvent residual signals: ∗ ethyl acetate

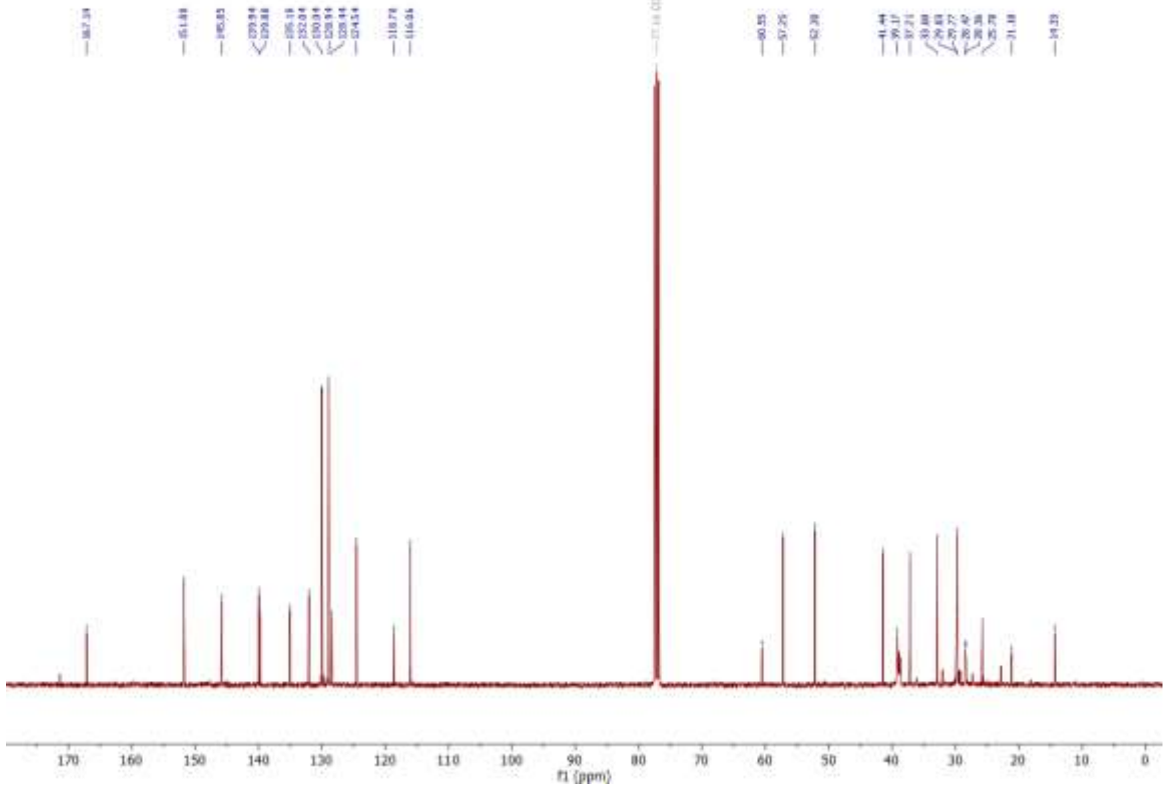

Compound **15**

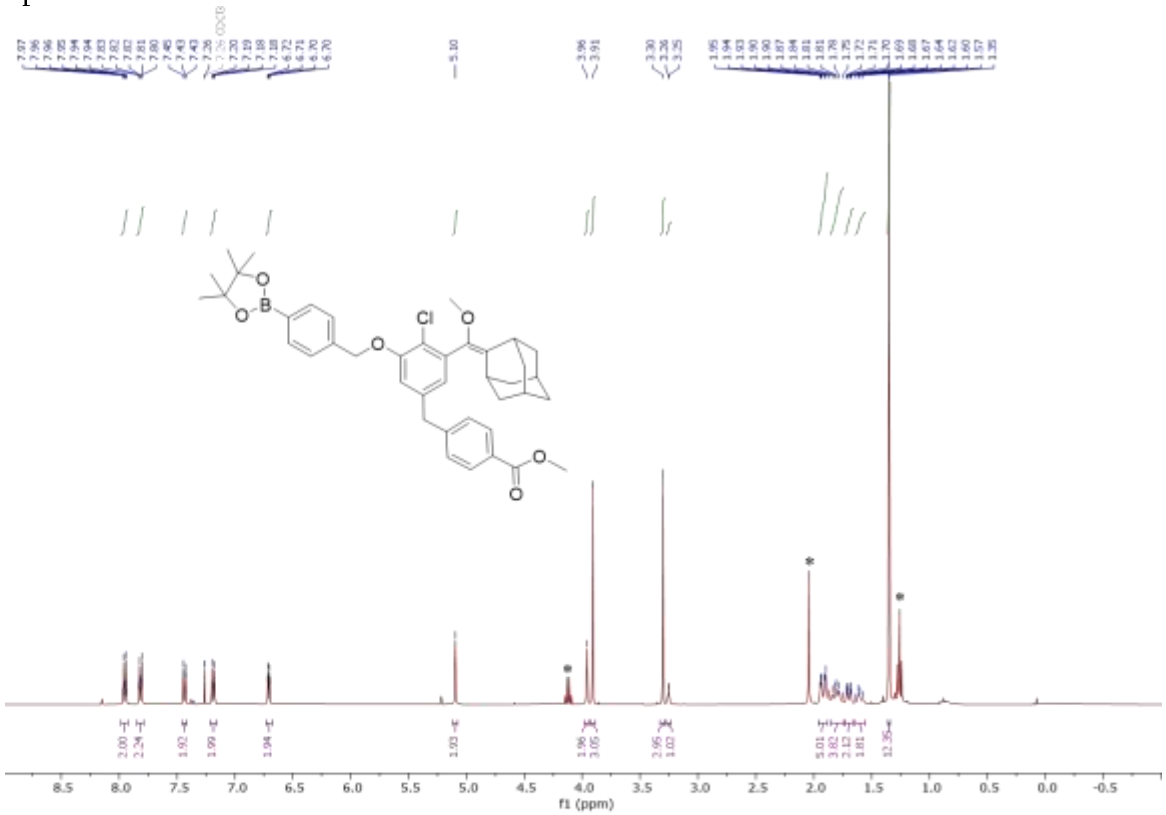

Solvent residual signals: ∗ ethyl acetate

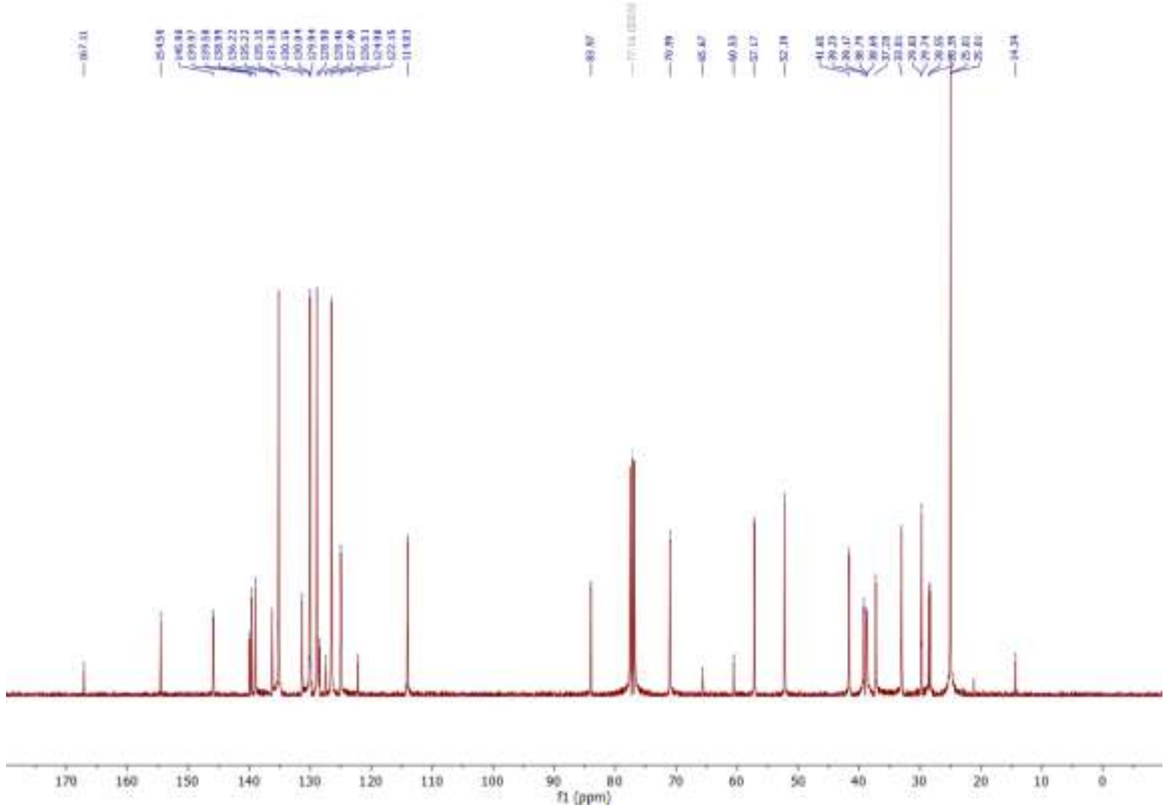

Compound **16**

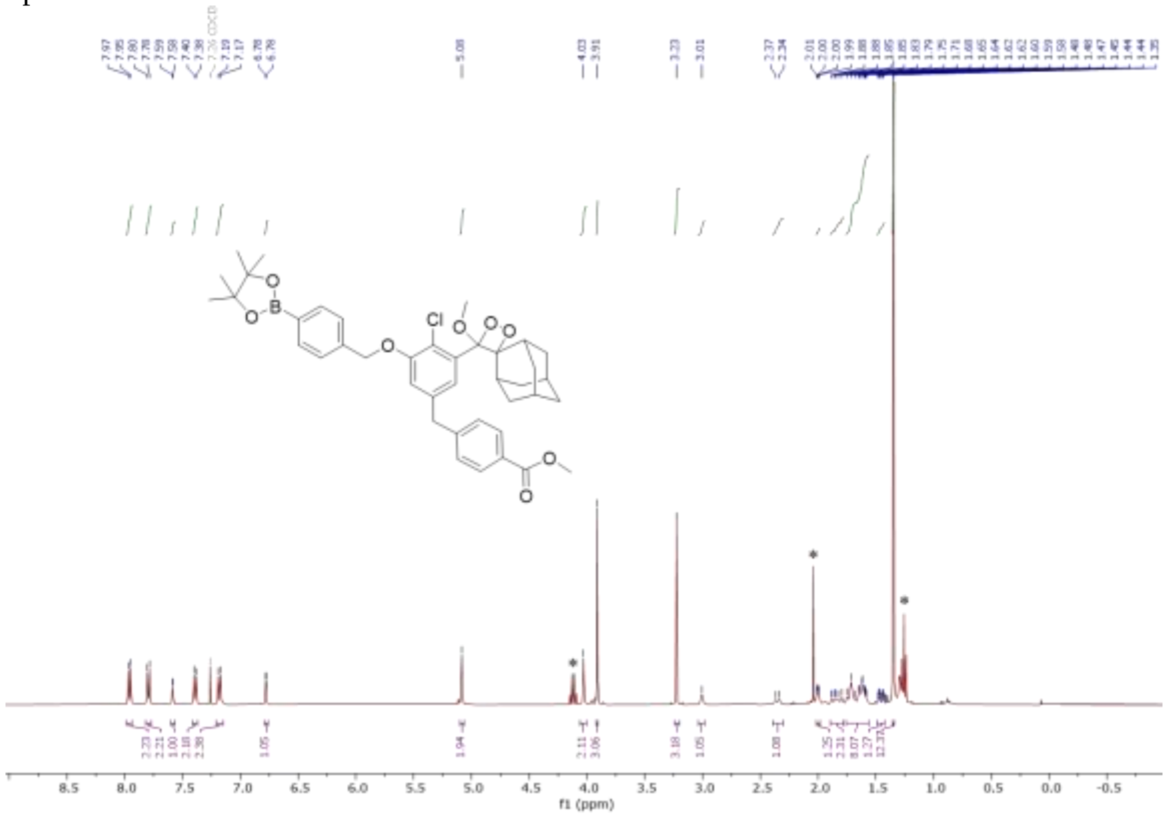

Solvent residual signals: ∗ ethyl acetate

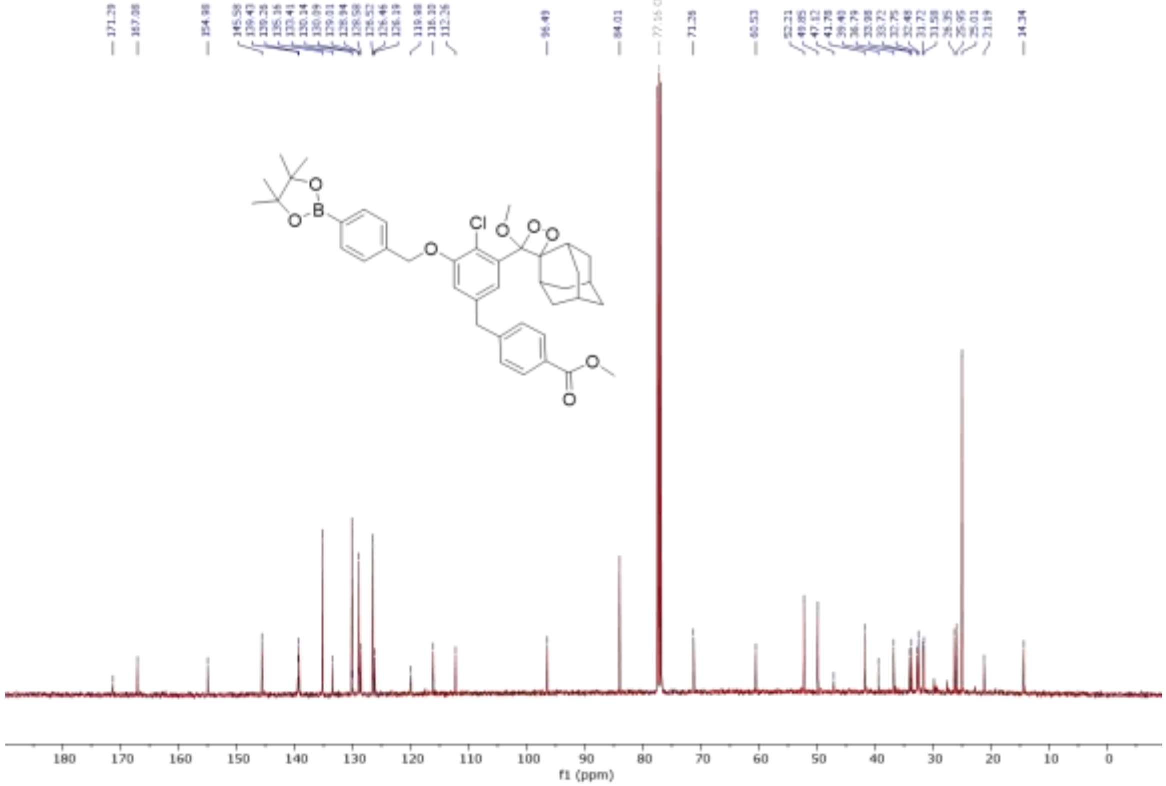

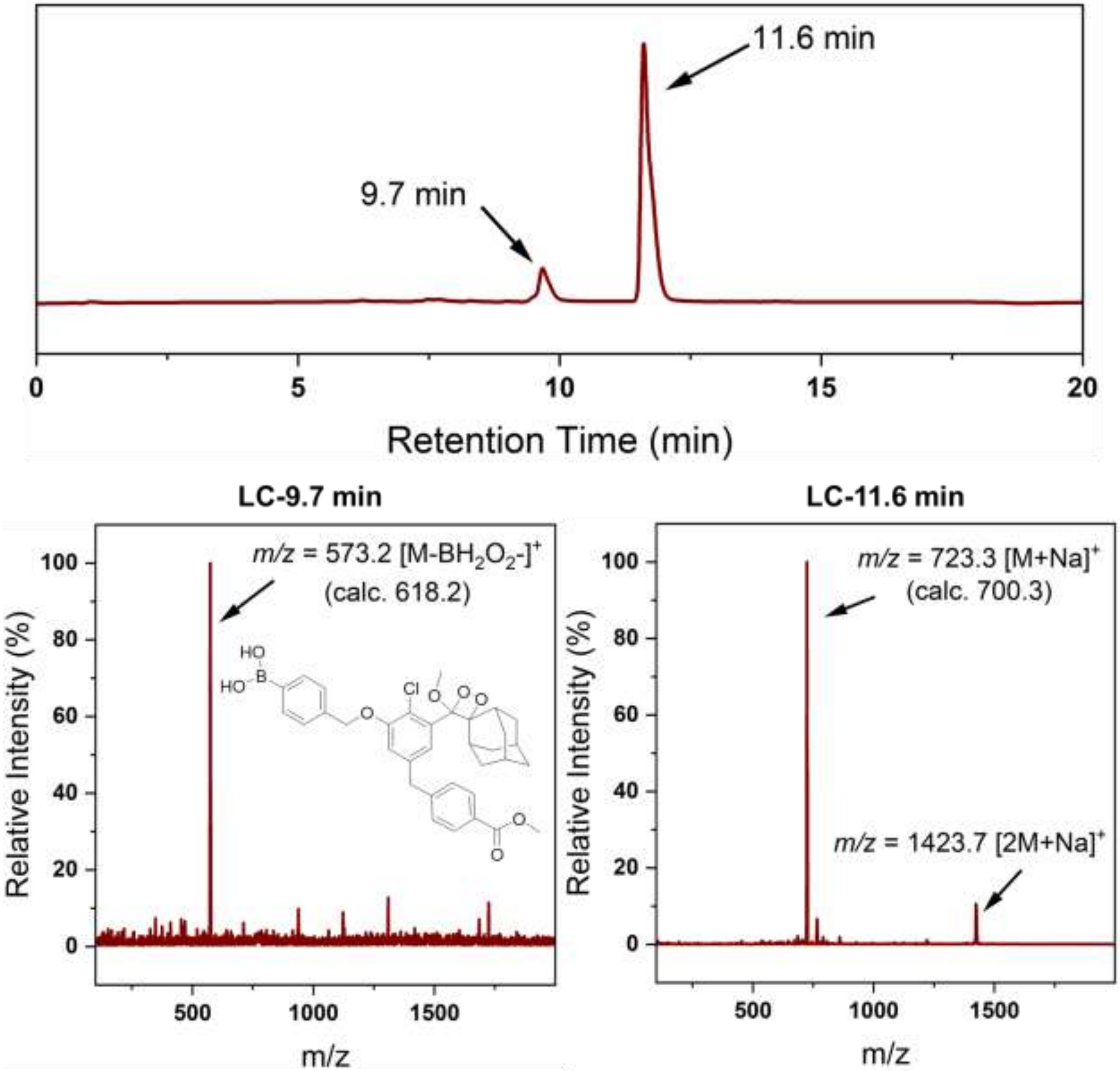

High-performance liquid chromatography - mass spectrometry of compound 16

Compound **17**

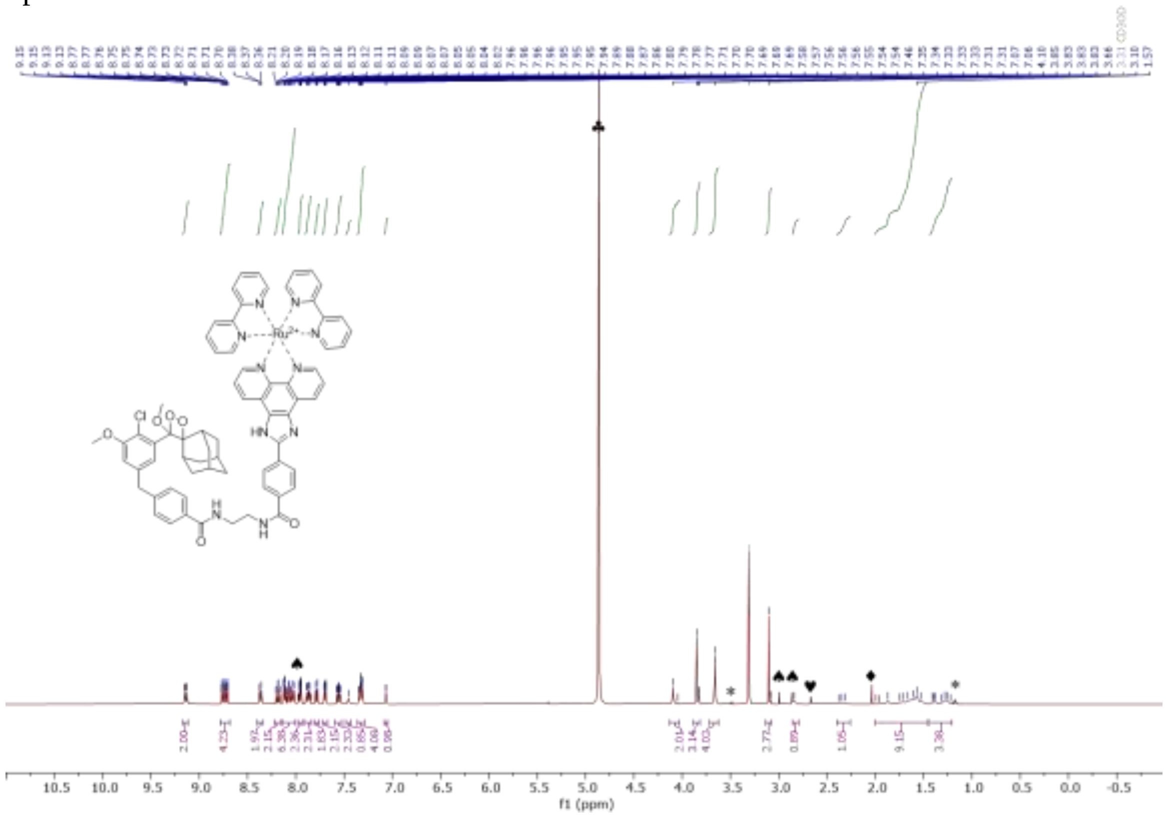

Solvent residual signals: ∗ diethyl ether, ♣ H_2O, ♦ acetonitrile, ♥ dimethyl sulfoxide, ♠ dimethylformamide

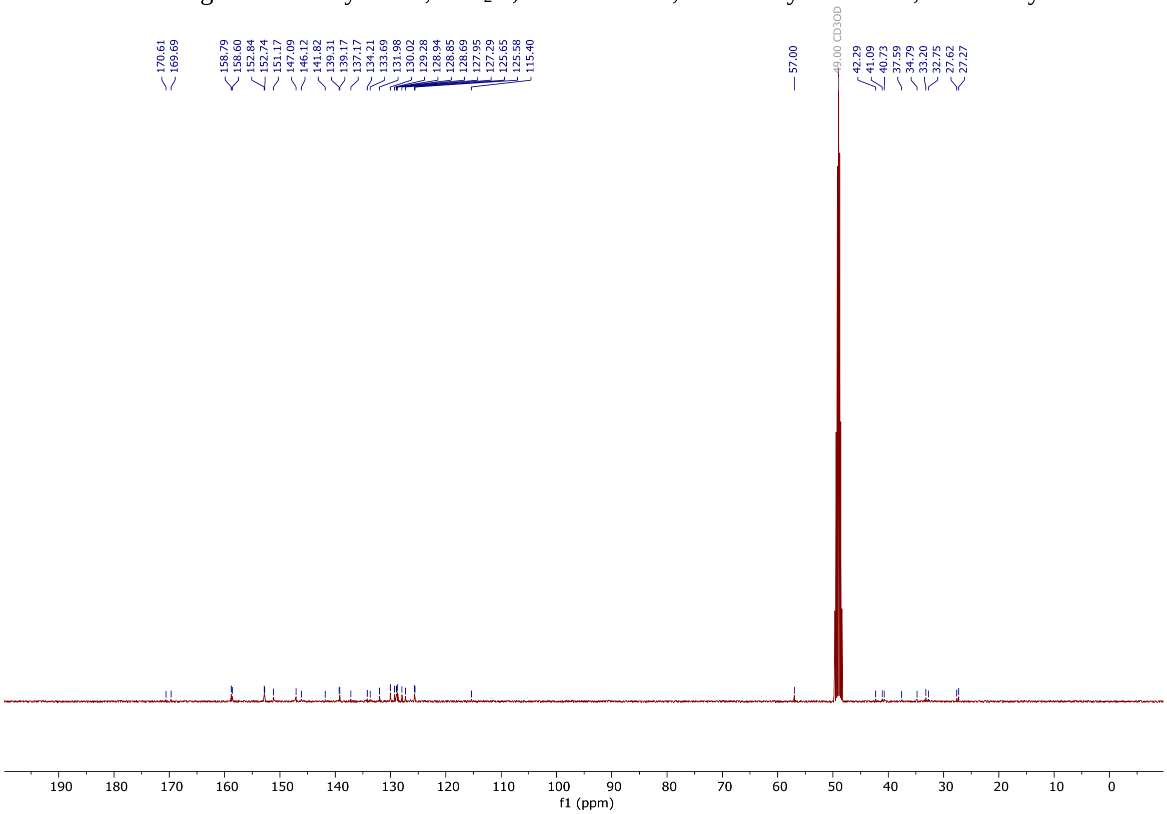

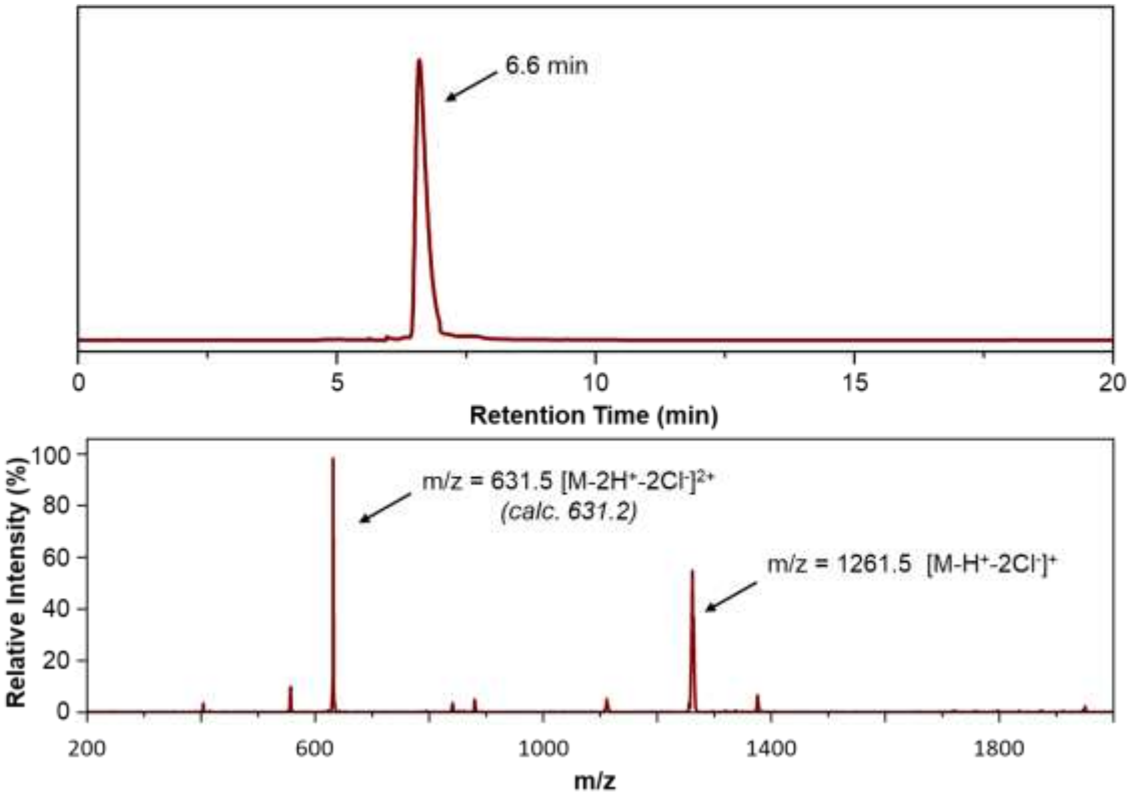

High-performance liquid chromatography - mass spectrometry of compound 17

4. Author contribution

Wenwu Peng: Initiating the project and chemical design. Designing, synthesizing and characterizing the molecules, performing cell experiments, data analysis and processing, drafting the manuscript.

Tianjiao Zhou: Designing, analyzing and processing *in vitro* experiments.

Lifan Hu: Perform experiments and data analysis for confocal uptake, colocalization studies and ROS studies of 3D tumor spheroids

Vivien Vankann: Establishing protocol for HaCaT cells, planning and discussing HaCat experiments with WP, writing the protocol for HaCaT cells.

Toszka Bohn: Providing HaCaT cells and establishing protocol, discussing the results, correcting the manuscript, co-supervision of VV.

Tobias Bopp: Providing protocols for HaCaT, discussing the results, correcting the manuscript, co-supervision of VV, acquired funding for the work.

Seah Ling Kuan: Discussion of the design, concept and results, correcting the manuscript, co-supervision of WP, TZ and LH.

Tanja Weil: Involved in the inception and design concept, discussion of the concept and results, correcting the manuscript, supervision of WP, TZ and LH, acquired funding for the project.

5. References:

(1) Hananya, N.; Eldar Boock, A.; Bauer, C. R.; Satchi-Fainaro, R.; Shabat, D. Remarkable Enhancement of Chemiluminescent Signal by Dioxetane-Fluorophore Conjugates: Turn-ON Chemiluminescence Probes with Color Modulation for Sensing and Imaging. *J. Am. Chem. Soc.* **2016**, *138* (40), 13438-13446.
(2) Gnaim, S.; Shabat, D. Self-Immolative Chemiluminescence Polymers: Innate Assimilation of Chemiexcitation in a Domino-like Depolymerization. *J. Am. Chem. Soc.* **2017**, *139* (29), 10002-10008.
(3) Simaan, A. J.; Mekmouche, Y.; Herrero, C.; Moreno, P.; Aukauloo, A.; Delaire, J. A.; Réglier, M.; Tron, T. Photoinduced Multielectron Transfer to a Multicopper Oxidase Resulting in Dioxygen Reduction into Water. *Chemistry – A European Journal* **2011**, *17* (42), 11743-11746.
(4) Saarbach, J.; Masi, D.; Zambaldo, C.; Winssinger, N. Facile access to modified and functionalized PNAs through Ugi-based solid phase oligomerization. *Bioorganic & Medicinal Chemistry* **2017**, *25* (19), 5171-5177.
(5) Estalayo-Adrián, S.; Blasco, S.; Bright, S. A.; McManus, G. J.; Orellana, G.; Williams, D. C.; Kelly, J. M.; Gunnlaugsson, T. Water-soluble amphiphilic ruthenium(ii) polypyridyl complexes as potential light-activated therapeutic agents. *Chemical Communications* **2020**, *56* (65), 9332-9335.
(6) Bhat, S. S.; Kumbhar, A. S.; Purandare, N.; Khan, A.; Grampp, G.; Lönnecke, P.; Hey-Hawkins, E.; Dixit, R.; Vanka, K. Tris-heteroleptic ruthenium(II) polypyridyl complexes: Synthesis, structural characterization, photophysical, electrochemistry and biological properties. *Journal of Inorganic Biochemistry* **2020**, *203*, 110903.
(7) Lin, C.-Y.; Huang, C.-S.; Hu, M.-L. The use of fetal bovine serum as delivery vehicle to improve the uptake and stability of lycopene in cell culture studies. *British Journal of Nutrition* **2007**, *98* (1), 226-232.
(8) Ng, D. Y. W.; Vill, R.; Wu, Y.; Koynov, K.; Tokura, Y.; Liu, W.; Sihler, S.; Kreyes, A.; Ritz, S.; Barth, H.; Ziener, U.; Weil, T. Directing intracellular supramolecular assembly with N-heteroaromatic quaterthiophene analogues. *Nature Communications* **2017**, *8* (1), 1850.
